# Self-care perspective taking and empathy in a student-faculty book club in the United States

**DOI:** 10.3352/jeehp.2020.17.22

**Published:** 2020-07-31

**Authors:** Rebecca Henderson, Melanie Gross Hagen, Zareen Zaidi, Valentina Dunder, Edlira Maska, Ying Nagoshi

**Affiliations:** 1 University of Florida College of Medicine, Gainesville, FL, USA; 2 Division of General Internal Medicine, Department of Medicine, University of Florida College of Medicine, Gainesville, FL, USA; 3 University of Florida, Gainesville, FL, USA; Hallym University, Korea

**Keywords:** Medical education, Medical/health humanities, Perspective-taking, Burnout, Empathy, Self care, United States

## Abstract

**Purpose:**

We aimed to study the impact of a combined faculty-student book club on education and medical practice as a part of the informal curriculum at the University of Florida College of Medicine in the United States.

**Methods:**

Sixteen medical students and 7 faculties who participated in the book club were interviewed through phone and recorded. The interview was then transcribed and entered into the qualitative data analysis program QSR NVivo (QSR International, Burlington, MA, USA). The transcripts were reviewed, and thematic codes were developed inductively through collaborative iteration. Based on these preliminary codes, a coding dictionary was developed and applied to all interviews within QSR Nvivo to identify themes.

**Results:**

Four main themes were identified from interviews: The first theme, the importance of literature to the development and maintenance of empathy and perspective-taking, and the second theme, the importance of the book club in promoting mentorship, personal relationships and professional development, were important to both student and faculty participants. The third and fourth themes, the need for the book club as a tool for self-care and the book club serving as a reminder about the world outside of school were discussed by student book club members.

**Conclusion:**

Our study demonstrated that an informal book club has a significant positive impact on self-care, perspective-taking, empathy, and developing a “world outside of school” for medical school students and faculty in the United States. It also helps to foster meaningful relationships between students and faculty.

## Introduction

### Background

While medical education is generally focused on the acquisition of biomedical knowledge, health professions educators have long recognized the utility of the humanities for reorienting trainees and practitioners towards the perspectives of their patients [1]. Literature in particular, has been examined as a potential pedagogical tool within health professions education due to its perceived usefulness in helping to foster empathy and professional development [2].

With this in mind, a number of institutions have implemented areas of formal curricula, such as narrative medicine electives, which include the guided reading of fictional or non-fictional literary texts, often centered on medical topics. Assessment of the outcomes of such electives frequently shows that their primary impacts are related to perspective-taking, communication, and empathy. For example, Arntfield et. al. [3] in 2013 explored the impact of a narrative medicine training on the professional development of medical students, finding that such courses may improve communication as well as the capacity for collaboration and empathy. In a 2017 review of literature on narrative medicine electives, Barber and Moreno-Leguizamon [1] found that such courses were frequently linked to improved communication, including perspective-taking and empathy, as well as personal and professional growth.

One alternative model for the inclusion of literature in the health profession’s curricula is the use of the “book club” format, in which a book is pre-selected, read by all students, and discussed in small groups. Book clubs within formal curricula have not only been demonstrated to increase student empathy [4], but have also been used as tools to develop teamwork, professionalism, and social identity through the maintenance of a positive and respectful group dynamic [5] within a medical team or to improve interprofessional communication [6]. The book club model permits small group discussions about non-medical topics, which facilitates students sharing their personal experiences with colleagues [7]. Furthermore, the more informal nature of these discussions can empower students, who develop communication skills by taking the lead in discussions [8].

### Objectives

We aimed to study the impact of a book club which is part of the informal curriculum at the University of Florida College of Medicine in the United States. The specific research question addressed is: How does the student-faculty interaction in an informal book club impact the personal and professional development of participants?

## Methods

### Ethics statement

This study was reviewed and approved by the University of Florida Institutional Review Board (IRB approval no., 201800793) with exempt status because the study poses minimal risk to the subject.

### Study design

It is a qualitative study through semi-structured interviews through phone call and email discussion. Derivation of main themes was based on the content analysis.

### Setting and participants

The University of Florida student-faculty book club was founded in 2011 by a medical student. The book club is voluntary and open to all medical students, residents, and faculty. The book club has met every few months on the weekends as convenient for members over the past 8 years. Each meeting is held at the home of a different hosting faculty member or student. Each meeting is generally attended by 3–7 faculty and 1–10 student members. Faculty members were predominantly Internal Medicine physicians. The books selected were mostly fiction and unrelated to medicine (Supplement 1). The book selected for each meeting is announced on a mailing list along with the time and place for the meeting.

When meetings begin, before starting a discussion of the selected book, students and faculty take time to chat on trips, family, or other topics that emerge. Often, light finger food is provided by the host. Then, participants gather around the hosts’ dining table or couches to discuss the book. Discussions generally open with participants sharing personal opinions on what they enjoyed or did not enjoy about the book. There is no guide to the discussion or questions the book club seeks to answer about the reading; instead, the discussion emerges organically. While the relationship of the book to medicine or medical practice often enters into the conversation, it is not the focus of discussions. It is not intentionally introduced if it does not naturally emerge.

As the discussion continues and feelings related to the book are shared, everyone is given the opportunity to talk regardless of hierarchy. When the discussion begins to wrap up, the group decides on which book to read next. This allows all participants a chance to voice any suggestions based on books they had previously enjoyed or heard about them. The date and time for the following meeting is also set at the convenience of the majority of members (always on a weekend). The effort is made to ensure the time chosen fits best for the majority of the group, including both students and faculty.

### Data collection and analysis

An initial interview script was constructed by author R.H. based on available literature. Two medical education experts (Authors Z.Z. and Y.N.) reviewed the script which covered topics such as book club experiences, memories of book club meetings, discussions, and books, reasons for attendance, and benefits of the book club. As pilot interviews, the interviewers interviewed each other as participants in the book club, then finalized the interview script (Table 1).

A convenience sample of past and current book club members were approached via email for interview. Seven faculty book club members (n=7/11) and 16 current and past student members (n=16/26) agreed to interview. The other 7 potential participants did not respond to the invitation to interview. The semi-structured interviews were conducted by phone by 2 female faculty (authors M.H., Y.N.) and 1 female MD/PhD student (author R.H.) over a 3-month period. Interviews with current student members were conducted by the student interviewer (author R.H.) to avoid coercion and to facilitate candid responses. All interviewers have previously experienced conducting qualitative interviews, and all had likewise participated in the book club and were familiar with its activities. As such, many interviewers had prior relationships with study participants, as peer colleagues (in the case of faculty) or peers (in the case of students). The research team explained their personal goals with regards to the research to all participants before commencement of the interview. As all interviewers had participated in the book club and helped to organize it, they held assumptions and potential biases regarding the utility and enjoyability of the book club. This positive experience with the book club was likewise the motivating force behind this research project.

Interviews lasted an average of 30 minutes each, and were audio-recorded and later transcribed by another student author V.D. Transcripts were not returned to participants for correction. A single interview was conducted for each participant; no field notes were taken as a part of the data gathering process. As the sample was a small known community, demographic details were not collected. All identifying information was removed from transcripts and transcripts were entered into the qualitative data analysis program QSR NVivo (QSR International, Burlington, MA, USA). The research team reviewed transcripts and agreed that thematic saturation had been reached. They then developed thematic codes inductively through collaborative iteration and a modified grounded theory approach. On the basis of these preliminary codes, a coding dictionary was developed and applied to all interviews within QSR NVivo by 1 primary coder (R.H.). Thirty percent of interviews were coded by 2 members (author R.H. and V.D.) of the study team to ensure coding consistency and data fidelity while the other interviews were coded by 1 researcher, followed by a review by the research team. The coding tree included parent codes that roughly approximated the main questions from the interview guide and child codes that encompassed thematically different responses to these questions. A member-check was conducted by presenting the results to a study participant for validation. The analysis used a modified grounded theory approach, drawing from the researcher’s experience with the book club as well as iterative modifications based on initial interviews. Researchers likewise employed a framework of critical reflexivity in the design, theoretical orientation, and implementation of the project. “Critical reflexivity” allows researchers to explore their relative positions on the topic. In keeping with critical research practice, the researchers employed critical reflexivity to explore their positions on the data set self-consciously. Phone calls and email discussions of themes allowed exploration of perspectives regarding the book club and preconceptions that may impact the data analysis.

## Results

Students and faculty interview transcript was available from Dataset 1.

Four main themes were identified from interviews (Table 2). The first theme, the importance of literature to the development and maintenance of empathy and perspective-taking, and the second theme, the importance of the book club in promoting mentorship, personal relationships, and professional development, were important to both student and faculty participants. The third and fourth themes, the need for the book club as a tool for self-care and the book club serving as a reminder about the world outside of school were discussed by student book club members. Each major theme was constituted by several minor thematic variants, as described below. In the quotes below “S” refers to students and “F” refers to faculty. For example, S04 corresponds to student number 4 in the database while F02 refers to faculty number 2. For complete interview transcripts, see Supplement 1.

### Theme 1: the development and maintenance of empathy and perspective-taking

Improved ability to understand others’ perspectives was reported by majority of participants. The improved perspective-taking came from both the books themselves (understanding the perspectives of characters) and from the discussions in group (understanding how other people read and thought about the book). The increased ability for “perspective taking” was noted to have an impact on participants both personally and professionally. Participants in the book club felt that perspective-taking was important in personal lives because it helped them grow as individuals, to become less judgmental, and develop an understanding of how others might be seeing the world differently.

For example, after reading “Hunger: a memoir of (my) body” a book written from the perspective of an obese woman, one student reflected on the ways that the book had made her less willing to pass judgements in her personal life:

“I think that I wouldn’t have said that I was biased against people who were obese in my personal life, like you know, I have friends who are overweight but I think when you’re—especially when you’re in medical school, you tend to be around really fit people who are really hyperaware of their weight, and when somebody isn’t that way, I think there’s a tendency to judge them. And I feel like I’ve become less judgmental. I think I’ve kind of opened my eyes a little bit to how many things might be going on in that personal life, you know, that they don’t necessarily have control over.” <S01>

One participant described the experience of having a discussion about a book they had personally disliked which allowed them to better understand the book and its merits. The student reported reflecting on this experience, which she said helped her to realize the value of such discussion, both in relation to literature and to medicine.

“I think, in a lot of ways, that first book that we discussed together, Buried Giant, had a large impact on me, mainly because I didn’t really enjoy that book all that much when I was reading it on my own, but when we sat there and discussed it I realized how nuanced and complex it was, and I found myself almost wanting to go back and start reading it again because when as I heard what other people got out of it and we started to kind of analyzing and thinking about it I realized that there was a lot of depth to the book and a lot of really interesting concepts to the book that I hadn’t seen before. I think it had an impact on me both in that it showed me personally the value of sitting and kind of talking through things, in and outside of medicine, and that there are other perspectives that can be had and sometimes you can learn a lot from other people and the way they perceive something. I suppose in some ways that may carry over into my professional life as well, in terms of trying to hear patient’s sides of their stories, and not just kind of go into an encounter or interaction with people based on—assuming that I know the answer, assuming that my perspective is always the right one.” <S04>

Many participants reported applying these insights to their professional lives as physicians or physicians-in-training. They felt that for doctors, empathy and perspective-taking are not only personally valuable, but useful in facilitating interactions with, and understandings of diverse patient populations. By engaging with fiction, in part through the book club, members felt that they cultivated an increased ability to understand their patients. As one faculty book club member put it:

“So, I would say those books, and many books I read, they offer me different perspectives to understand people from different backgrounds. You know, people who are older, people who are younger, maybe people who are from different countries, different cultures, speak a different language—unless you read a book and you understand views from people’s perspectives, you may not consider their viewpoint, and you may not understand how it affects their decision-making. So, it’s really huge because in my professional life. I’m an Internal Medicine Physician. I see so many patients from so many backgrounds, and I don’t always understand the context of why they might be choosing to do something or not do something. But, reading about things offers me a little more wealth, it allows me to draw from an experience pool that I don’t have, that others have. And I can kind of glean from that wisdom, from those perspectives, or I can understand sometimes the value of not judging because I’ve read enough to say, okay, I can imagine a character from a book in this situation, why would he make this decision, why would you do this choice—because on the surface it doesn’t make sense, but when I consider it from that perspective that I learned about from fiction even more so than real world people sometimes, it’s definitely—the context changes dramatically, absolutely.” <F02>

### Theme 2: promoting mentorship, personal relationships and professional development

One of the must universally appreciated features of the book club was the opportunity for students and faculty to get to know each other on a more personal level, outside of the structures and hierarchies of the clinic. Students in particular felt that this feature of the book club created an almost unique setting in medical education in which there was no pressure of being evaluated and both faculty and students felt comfortable to express themselves freely on a topic where there was mutual enjoyment and a relatively level playing field of knowledge and opinion. They especially enjoyed talking about topics that would normally not be considered “professional.” A student commented:

“So, I think that’s been one of my more favorite parts because it’s pretty rare to get to have such a personal connection and feel like I can talk about things like politics or religion or sex or things like that with faculty members, because usually those are taboo topics. So, that’s been something I’ve really enjoyed- just getting to know them a little bit more and kind of realizing that it’s okay to sort of discuss our opinions and talk about things completely different than medicine, which is normally all I get to talk to them about.” <S02>

This ability to get to know faculty members as people, rather than simply as physicians, helped students to find their way professionally. The opportunity to talk about books allowed them deeper insight into how professional mentors relate to patients, and how medicine fit into their personal lives. As one student described:

“It’s interesting because, when I was a fourth year medical student going to book club, all of the students that went regularly turned out to be internal medicine residents, so I wonder if that partly could have contributed to my choosing of internal medicine because so many people in book club were internal medicine, and they were people I looked up to and had the type of life that I wanted.” <S03>

Faculty members of the book club also reported enjoying the opportunity to get to know students outside of more formal academic settings, appreciated the lack of hierarchy and reported being surprised and impressed by the insights that students can bring to the book club. As one faculty member described:

“I really like the opportunity to interact with the students on the same level and I think that they often shed a lot of insight about books that—because they come from a different perspective. It’s just really interesting to see how someone who’s not married, doesn’t have children, hasn’t had a career, can really have a lot to contribute into books that are about what I might consider “about life,” about the difficulties of life. I think at first, I thought I might feel I have had to teach them something, but really, I learned more from them at the book club…I think the students have a much stronger, more equal voice and it’s fun to see that. We’ve had some that have had really strong personalities and opinions and have been much more knowledgeable about the authors in the literature than the physicians, so they’re really taking the lead role and it’s fun to see that.” <F03>

Finally, faculty members of the book club valued these meetings because they allowed them to develop friendships and relationships with their own peers who they would otherwise not have interacted with outside of a professional setting.

“I’m so grateful for this opportunity because we’re so busy with our daily grind of seeing patients, so this gives me opportunities to see colleagues outside of work environment. And then we talk about things outside of the book too, like where to buy the best pastry, what are the shows to see. So, you connect more on a personal level, than just a professional level like before.” <F04>

### Theme 3: book club as a tool for self-care

An almost universal theme present in interviews with current and former student members was the role that the book club played in helping them to prioritize reading as an important self-care activity. Students noted that reading had been an important part of their lives prior to beginning medical school, but was difficult to maintain and prioritize in the face of competing responsibilities. They felt that while reading played an important role in their well-being, it was difficult to find time for non-school activities and that even the most crucial self-care activities fell by the wayside due to the demanding nature of the medical curriculum:

“In medical school, I really had trouble making time for reading in during the school year, I just didn’t do it. And it’s something that’s been important to me my whole life. Like, reading fiction is how I self-care you know what I mean? And during medical school, I completely did not have time to do that at all, even when I had vacations and stuff I didn’t have time for that.” <S04>

However, the book club allowed students to make time for reading and thinking about literature in several ways. Students expressed that seeing faculty members actively prioritizing and valuing literature as a part of their free time activities helped them to see it as important. Likewise, students felt that faculty members valued their participation in the book club helped them to see the importance of reading. A student commented:

“I think it’s one thing to preach the whole ‘well-rounded’ and ‘wellness’ thing, but when you’re meeting with faculty members and they are doing these kinds of things…While the book club may not be for everybody, for people who like to read generally it is something that helps them to feel good and to have balance in their life, you know.” <S10>

The book club meetings also set low-pressure deadlines that helped medical students justify fitting reading into their busy schedules. The date of the next meeting provided a “deadline” that helped students to budget time for reading and prevented it from being put off indefinitely. As it was a relatively small group of students and faculty, students said that they did sometimes feel pressure as a part of this community to finish the book. However, this pressure was described as necessary, rather than onerous:

“So, if I don’t finish the book I don’t feel pressure, but it’s just like a little bit more justification, you know. Like I couldn’t justify it just for myself, but because I have the book club, I can justify it, you know what I mean?” <S06>

As the book clubs were optional and geared towards pleasure reading, students knew that there would be no consequences for skipping a meeting, or arriving unprepared. Students said they felt comfortable going to meetings at times when they had not fully completed the book because they knew their contributions and presence would be valued even if they hadn’t finished the book. As one student described:

“I’ve always been someone who enjoyed reading, but it’s so easy in medical school to just tell yourself oh you don’t have time to make yourself a healthy dinner, to exercise, to do activities you might have enjoyed before, like reading. So, having something like the book club not only gave you like a timeline to finish something, but it also made you kind of reflect on that experience, so it felt like a very worthwhile way to spend your time. You know, it didn’t feel like you should’ve been studying for a test instead of reading the book club book. So, I think that was really important. I also think it’s hard to force yourself to take some quiet time during the day. You know, we’re so busy and running around and going from one task to another, the time to just sit and read was really nice.” <S07>

### Theme 4: the book club serving as a reminder about the world outside of school

A final theme that emerged from the interviews was the role that the book club played in helping students to maintain a space in their lives that remained separate from school. Many students commented that during medical school they felt that their lives were consumed by academics, leaving little room for activities, identities, or ideas that were not related to the experience of medical education. Students commented that they rarely talked about non-medical topics or engaged deeply with ideas unrelated to medicine during their time in school. A former student commented:

“I think medicine is a field where it’s really needed for you to jump all in and really lose your sense of balance, if I might. I think there’s a lot that is wonderful in the field, a lot of good from it, but that’s a very real dark side. Even as a student I saw that- as a medical student it was necessary to shed the parts of life that aren’t important for the sake of just trying to be all in. And while I think it’s important to be excellent at your work and to strive to be a really phenomenal physician, I also think it’s really important to be a balanced and real human being.” <S08>

Students commented that they deeply valued the book club as helping them to maintain a “non-medical space” in their lives. For students to whom literature and reading were already important before medical school, this non-medical space was especially important as it allowed them to feel that a vital part of their personality was being maintained and nourished:

“Well, I think I just really love to read and it’s something I try to do myself. I remember starting medical school and feeling like a lot of my creative juices were already dry because it’s a lot of just memorization and a lot of just thinking about science and a lot of about just kind of the same thing, and it was an outlet for me to just kind of explore my humanity, at least in the first couple years of medical school. So that—it was definitely very nourishing to have this time to really read and just enjoy in a very peaceful setting.” <S09>

The choice of non-medical books further validated the presence of a world beyond medicine in the lives of students. Further, book club participants valued the ability to talk to peers and faculty about non-medical topics, and to build relationships that were not built around medical professionalism:

“I think it helps people bond, because I think in medicine people—for the most part, physicians tend to not talk about things other than medicine. They forget to talk about the things they love outside of medicine, there’s almost an unspoken ‘you’re not supposed to talk about’—or ‘you’re not supposed to have a life outside of medicine.’ So, I think that the book club nurtures that and helps people step outside medicine. Although, I do find that sometimes it reverts back to medicine.” <F06>

## Discussion

### Key results and their significance

Our study showed that an extracurricular book club involving faculty and students help develop perspective taking skills and build empathy, dismantle hierarchy while promoting relationship building between faculty and students, help participants focus on self-care and wellbeing, and prevent living strictly within the silo of the profession of medicine.

These results are important in the light of research showing a significant drop in empathy among medical students over the course of training especially at the start of the clinical years. Empathy is an important component of physicians’ competence as it has also been shown to lead to increased patient satisfaction, compliance, and improved clinical outcomes [9,10]. On the other hand, decline in empathy is linked to stress and burnout, with one study documenting that half of medical students suffered from burnout [11]. It is therefore imperative to build programs focusing on stress management, wellbeing, professional development, and identity formation of students. Such programs can either be formal and offered as part of the medical school curriculum, or informal as in our book club. Chisolm et al. [12] in 2018 suggest that the use of an informal book club among physicians may be a way to increase a sense of community, reduce burnout, and return joy to medicine. Our research demonstrates that similar results may be achieved among students through the use of the informal book club format.

Our results further show that reading fiction helped students develop perspective taking skills, and the ability to perceive a situation from an alternative point of view [13]. Perspective taking helps students take into consideration not just the biological aspects of disease but also the socio-cultural and mental elements of health [14].

Additionally, our study shows that creating forums such as this book club outside the formal medical curricula helps dismantle hierarchy between learners and faculty, thus resulting in bi-directional meaningful exchanges. Since the majority of the student-faculty interaction in medical school occurs in busy hospital or clinic setting, students and faculty enjoyed being able to interact outside a professional setting. The book club meetings helped cultivate stronger mentorship and relationships on a professional and personal level. The informal setting encourages students to interact with faculty, allowing them to discuss topics in a “safe-space” which may have otherwise seemed off limits. Students valued the role the book club played in encouraging self-care by taking the time to read, specifically noting that the active participation of faculty members legitimized reading and validated student’s desire to read. Faculty treated reading for pleasure as a valuable activity and modeled reading non-medical books as a positive activity for physicians. Our study confirms the importance of extracurricular activities in breaking the daily routines of medicine and medical school. Bonding with peers, including faculty and students, who share similar professional challenges and have similar lifestyles, is likely to lessen the burden of burnout while at the same time inspiring students and residents to lead a balanced lifestyle. These findings confirm the positive impact that incorporating literature and humanities in medical school curriculum has on increasing empathy towards patients and improving rapports with patients and physicians.

### Limitations and generalizability

One of the limitations of our study is the small sample size. However, the richness of the responses provides a starting point for others to build on this work. Participants of the book club were self-selected, and as avid readers, may have been prejudiced regarding the impact of the activity compared to their peers. However, we suggest that this self-selection is important in building the book club community as a voluntary project composed of students and faculty who valued reading and talking about books. While we did not use a burnout scale to study the impact the book club, the study highlights the importance of informal extracurricular activities leading to positive social interaction, which we suggest may in turn lead to decrease burnout, although further studies are needed to substantiate this connection. Finally, some interviewer and interviewee had prior relationships which might lead to biased interview result. However, we made every effort to reduce this bias by matching interviewer with interviewee whom they are less familiar with. Current students were only interviewed by student interviewer to reduce possibility of coercion and to facilitate candid responses.

### Conclusion

Our study documents the positive impact of an extracurricular book club which deliberately brings students and faculty together. The book club served as an outlet for stress management and enhanced empathy by introducing members to different perspectives, and by fostering relationships in a more relaxed, “non-medical” setting. Furthermore, the book club helped to develop personal and professional relationships and to remind students of the importance of self-care and non-medical activities in their daily lives. While narrative medicine and reflection essays are now a part of formal medical curriculum in most US medical schools, our study invites medical educators to consider the use of literature in an informal setting to foster empathy, reflection as well as personal and professional growth.

## Figures and Tables

**Table 1.** Interview script for both student and faculty interview

	Interview script
1.	How did you get involved with the book club? Do you remember your first meeting, what was it like?
2.	What’s a book that you remember having an impact of you? What impact?
	a. Professional life: Clinical care? Interaction with patients?
	b. Personal life
3.	What are your interactions like with members of the book club?
	a. Interactions with students?
	b. Interactions with physicians?
	c. Interactions with faculty?
4.	What is the benefit of the book club for you? Why do you attend?
	a. Is there an element of self-care?
	b. Does the book club make you read more? Why is that important?
	c. Are the books that the club reads books you’d choose for yourself?
5.	Have you ever been exposed to literature as part of your **formal** medical training? What was that like? How was that experience similar/different than this book club?

**Table 2.** Book club impact themes

Theme	Description	Example
The development and maintenance of empathy and perspective taking	Description of how the book club opened up the participants’ understanding of other personal or cultural backgrounds and the impact of life circumstances, resulting in increased compassion in both the personal and professional lives of participants	“I think reading in general provides empathy in terms of learning about others and I think that when you get to share that with others, especially in our clinical field and to share stories of our experiences, it’s hard to not think that that’s impactful and the care that we provide for our patients and for each other.” (By current medical student)
Promoting mentorship, personal relationships, and professional development	Students described how they have been able to grow and foster relationships with the faculty they interact with at book club outside of the academic setting, allowing them to get to know faculty personally while dismantling hierarchy	“I think it’s important to have situations where faculty and students are getting together in less formal settings and on more equal footing, I think that that is a great part of the aspect of the book club.” (By current faculty member)
Book club as a tool for self-care	The role that the book club played on improving the individual’s overall wellbeing. Such as: allowing students to justify making time for a positive outlet outside of their studies a priority	“I think there’s a lot of talk in medicine now about burn out and stuff, and I think anything you get to do things with other students, in a friendly environment, that kind of takes away from stressors and it was great. For me, I looked forward to them. They were definitely something that helped me avoid burnout.” (By former medical students)
The book club serving as a world outside of school	Book club acts as a reminder for the students that there are other things to engage with that are not medical school related. It allows them to connect with other individuals in a more personal way, as well as to literature in a non-clinical manner	“It just forces you to make plans for other things you enjoy doing that are not work, so being kind of coerced almost into getting your reading done- it was good for that.” (By current medical student)

## Footnotes

**Data availability** Data files are available from Harvard Dataverses: https://doi.org/10.7910/DVN/7HDDDM Dataset 1. Students and faculty interview transcript.

## Supplementary materials

Data files are available from Harvard Dataverses: https://doi.org/10.7910/DVN/7HDDDM

Supplement 1. The books selected for the book club

Supplement 2. Audio recording of the abstract.
