# Supplementary material for: Self-care perspective taking and empathy in a student-faculty book club in the United States
Source: J Educ Eval Health Prof. 2020 Jul 31;17:22. doi: 10.3352/jeehp.2020.17.22 (PMC7577880; doi:10.3352/jeehp.2020.17.22)
Supplement: Supplementary file 1 [file jeehp-17-22-dataset1.pdf]

Faculty 1

Interviewer: How did you get involved in the book club?

Participant: Well, I actually started the book club with a student, NAME. She was assigned to me as an advisee, because she had said she was interested in Internal Medicine when she started medical school, although she ended up becoming a general surgeon. And we were just talking, and she told me she was a literature major in college and we talked about how we like to read and we liked to be in a book club, and that led us to start the book club.

Interviewer: Do you remember your first meeting?

Participant: I actually don't remember the first one. I remember some of the very early ones in her apartment, that I think she shared with one of the other book club members, and I think we read a book of short stories the first time so people could join in even if they didn't have much time and could only read one story. We also read "Cutting for stone," I think, which is a book about a physician.

Interviewer: What's a book that you remember having an impact on you?

Participant: So—

Interviewer: And what impact?

Participant: What impact, yeah. So, I think some of the books that are from the point of view from a person who you might think of as a difficult person, like "Olive Kitteridge" or "The Brief and Wondrous Life of Oscar Wow" or one of the characters in "Imagine Me Gone." I think that they shed some insight into people's behaviors- why they act the way they do. So, those are some of the books that I remember. I also remember coming to book club and not really understanding the book or not really feeling I got much out of it, but after hearing the perspectives of other people in the book club it enriched my experience of the book. For example, with "Life of Pi" or "The Buried Giant."

Interviewer: Professionally, how does it impact you? Well, in terms of patient care.

Participant: In terms of patient care, I think that all books— all fiction that helps you see the perspective of people that are not like you are helpful in increasing your empathy and understanding the kind of experiences that your patients have had when they come to see you in clinic. In terms of poverty, or being victims of interpersonal violence, and that kind of thing.

Interviewer: How— does it impact your personal life?

Participant: Well, again I think seeing other people's perspectives is helpful in seeing your interactions with your family members. But, more so probably with the people I meet with patient care.

Interviewer: And what are your interactions with the members of the book club, such as how do you interact with the students?
Participant: I really like the opportunity to interact with the students on the same level and I think that they often shed a lot of insight about books that— because they come from a different perspective. It's just really interesting to see how someone who's not married, doesn't have children, hasn't had a career, can really have a lot to contribute into books that are about what I might consider "about life," about the difficulties of life you. I think at first, I thought I might feel I had to teach them something, but really I learned more from them at the book club.

Interviewer: How about interactions with other physicians?

Participant: It's a great opportunity, we rarely get to sit and talk about anything— we rarely get to sit around and talk about work, much less non-work issues. So, it's a great way to get to know people better over time and to really connect with coworkers. And, I also really like the opportunity to hear experiences of people from different backgrounds. We mostly have women in the group, but whenever there's a male there we're always really hungry to hear what the male perspective is on the particular book. And it's nice to hear the younger perspective and sometimes we've had faculty members who are older, you know maybe in their late 60s or 70s, people with children, people without children, and it's really interesting to hear their perspectives.

Interviewer: What is the benefit of the book club for you? Why do you attend the book club?

Participant: Well, I like to read, but I don't always have time to read and sometimes I'll start a book and not really be motivated to finish it but the book club makes me read things I might not have otherwise read and it makes me finish the book, and think about the book.

Interviewer: Is there an element of self-care?

Participant: Well, that's what's interesting. I usually find it a little stressful trying to get the book read before the book club, and I feel busy during the week so when the weekend comes there's a lot to do on the weekend and I sometimes, especially in the beginning maybe, felt that "ugh I have to go to the book club." But, then I always leave the book club feeling well, feeling peaceful and relaxed, like after meditation or yoga or something. So, that's what keeps me going.

Interviewer: Does the book club make you read more?

Participant: Yes, it does, because I'll read books that I need to read for the book club, but then I'll say, "well I want to read something on my own" and then it inspires me to finish those books too, so I can recommend them to people in the book club.

Interviewer: Why is that important?

Participant: Well, reading has a lot to offer. It helps you to become more educated, and reading is also a good way to unwind and relax and to disconnect from electronics; it's really hard to

disconnect from phones and computers and reading is an activity that doesn't involve electronics so.

Interviewer: Does the— are the books that the book club reads books that you chose for yourself?

Participant: Sometimes. Yeah, some of the books are and some of the books aren't. So, that's been good.

Interviewer: Do you— have you ever been exposed to literature as part of your formal medical training?

Participant: No, I have not.

Interviewer: Oh, that's funny. It said it was going to stop. I think it's easier to change than completely transcribe.

Participant: Oh, absolutely. Absolutely.

Interviewer: Hopefully it's not total gibberish.

FACULTY 2

Interviewer: Okay, so I'm going to start asking you questions. What did you— how did you get involved with the book club?

Participant: I was invited by Dr. NAME.

Interviewer: What was your— do you remember your first meeting?

Participant: Yes, I do. It was a small, intimate gathering with exhaustive and extensive and thorough discussion about the book, and unfortunately, I do not remember the name of the book.

Interviewer: What's a book that you remember having an impact on you?

Participant: One that I always remember is "Land Remembered." It had an impact personally in terms of relating— I'm sorry, in terms of being compassionate, generous, and understanding to others and always giving, is what I remember about that book and that is the personal aspect of my life. In terms of professional life, the book that did impact me was the book "Hunger" by Roxanne Gay. It definitely put a whole new perspective in terms of relating to obese patients and not chalking up everything to weight as their problem. And also, understanding that they have significant depression issues because of their obesity and their inability to relate to others. And also, in terms of obese patients, not to stereotype them.

Interviewer: So, from a professional stand-point view, you already mentioned, it impacted patient care— you mentioned "Hunger," right?

Participant: Yes.

Interviewer: And how to deal with obese patients and personal life- I think you kind of went through both of them already. What are your interactions like with members of the book club- with students?

Participant: It's been great. It's rejuvenating. It's been— learning to interact with them in terms of their younger minds and their thought process is sometimes different and it opens up the perspective on what the book is about, that I may have missed. So, I thoroughly enjoy interacting with the students.

Interviewer: What about other physicians?

Participant: In terms of physicians, I think I get to see them outside their work environment; I don't see them just as colleagues but also as friends, and I've come to see them as friends because I'm seeing them outside of the work area.

Interviewer: What is the benefit of the book club to you? Why do you attend?

Participant: For me, the book club is intellectual stimulation, great discussion, and a lot of times I read through the book and when we discuss some of the topics from the book, I sometimes realized I completely missed it. Or missed what the book was headed to, and so I think it opens up my mind and it opens up all the information that— what the author is trying to relate to the audience.

Interviewer: Is there an element of self-care?

Participant: Yes, absolutely. I think there's mental preservation; it stimulates my thought process, especially talking to others and discussing the variety of issues that are brought up in the book.

Interviewer: Does the book club make you read more?

Participant: No. Since I'm an avid reader, I don't think it makes me read more.

Interviewer: Are the books that the book club reads that you never would have chosen for yourself?

Participant: Yes, yes- that is the reason I am in the book club because there's no— there are so many books out there that I would have never chosen and the book club chooses and therefore it is— it opens up a whole new venue for me.

Interviewer: Have you ever been exposed to literature as part of your formal medical training?

Participant: No. I've never been exposed to it.

Faculty 3
Interviewer: So, this is Dr. NAME, a pulmonary physician. So, how did you get involved with the book club? Do you remember your first meeting? What was it like?

Participant: Okay, I got involved because NAME mentioned the book club to me a few times. We liked to talk about and share books so, she said "do you want to come?" And it took me a few tries to be able to work it out to come, and I think the first meeting I went to was at her house, and it was a discussion about **[unintelligible 00:00:40]**- I think it was *Hunger*, that was the book.

Interviewer: That's right, I think so. So, what's a book that you remember that had an impact on you? Can you hold on for one second? **[To Someone Else in Room]** Can you take the dog? Please, I'm interviewing; she is making a lot of noise. **[Speaking to Participant]** Sorry, my dog is making a lot of noise. So, let me say again; what's a book that you remember having an impact on you, and what kind of impact?

Participant: Well, the book—

Interviewer: I know sometimes it's hard.

Participant: Out of the books I've read so far, I liked the book *Breakthrough* the best. And it's a—it was, you know, it was on a medical topic. It was the discovery of insulin. But, it was just so interesting how it all came about, and it gave a backdrop on the history of the time. I just found it interesting in terms of comparing to how things—how medicines are discovered and put through the process nowadays, compared to that.

Interviewer: Yeah, very different. Is there any books that you feel impacted your personal life?

Participant: Well, not to change it. It was interesting because the book *Hunger*—first of all I feel like they choose, in general, very depressing books that I would not ordinarily read. But anyway, it was interesting because the book *Hunger*, I didn't say very much. It was all these people who had never been fat discussing the book; it was interesting because I have quite a different perspective on it and have been struggling all of my life and up and down on diets and losing a lot of weight and various things that—I felt like I had a lot more personal connection and a density with the person writing that book.

Interviewer: You didn't feel comfortable talking about—

Participant: Correct.

Interviewer: Wow. I would love to hear sometime maybe again, not with the whole group maybe. So, it did—you understand the book? You understand what she's talking about?

Participant: Yeah, I think I understand it, much differently and much better, I think, if you've had some of the same experiences. Some of the books I didn't finish because, you know, I think it was *Exit West*. It was actually before I was able to go to it. I tried to read that book, but I just couldn't read that book. And, *American Marriage*, I also could not get into that. You know, I think as I've gotten older, it's much more difficult to for me to force myself to read a book that doesn't really catch my interest. I just can't do it, it's just a chore. It's not enjoyable.

Interviewer: And you don't need to, really.

Participant: And I feel like my time is so limited.

Interviewer: Right, right. You want to enjoy—you want this to be an enhancement of—yeah. Of course. That's what the book club is intended to be. It kind of makes you read more, but not intended to be a formal thing kind of like, people have to—

Participant: I like the book *Middlesex* very much, and I never would have read that book if I hadn't been at the book club.

Interviewer: Me too, me too. Yeah, I loved that. I mean, I knew about this book for a long time, and I was just like, oh it's just one of those topics about, you know, it just didn't attract me, until you read the book. It's not just about that at all.

Participant: Yeah, it was a very good story. It was good—

Interviewer: With a lot of history and things like that, yeah. Did you enjoy the Florida one? What is it about—I didn't know if you were there.

Participant: I wasn't there. I had read the book before, years ago, and actually I had given it to NAME—

Interviewer: Oh, you recommended it to her- *Land Remembered*.

Participant: Yeah, I thought that was a great book.

Interviewer: Yeah, I loved it. I have since recommended it to a lot of people. I tell them that you have to—it kind of enhances your understanding of the land.

Participant: Oh yeah, and what life was like before development, it's amazing. The malaria and how long it took to just go from coast to coast, it's just amazing. I mean even—it's just amazing. That book was originally given to me by NAME.

Interviewer: Oh, really?

Participant: [crosstalk 00:06:45] the Department of Medicine. Yeah, one time I was in his office and I can't even remember what we were talking about, and he was reading the book and he goes "this book, you've got to read this book." So, he passed it on to me and I passed it on to NAME.

Interviewer: I see, you're the source of it. I see. So, cool. What are your interactions like with members of the book club? How's your interactions with the students?

Participant: Well, let's see. Sort of, you know, I would say sort of minimal, except for the one time I had it at my house, only three people came- one of them was a student.

Interviewer: Two of them.

Participant: Yeah, two of them were students, okay. So, that was the one time, especially if they came a little early, that I had some interactions with students. But, I guess they don't really know me. I get a lot of- it's a pre-med student I believe—

Interviewer: No, one of them is earlier, like a first year, or second year. A first year. Then the other one is obviously a combined MD/PhD person.

Participant: Oh, and there's another one who's a pre-med student, because I've seen her a couple of times. I can't remember—she works in someone's lab and she got—anyway, she was one of the ones that came to my house. So, I haven't had too much interaction with the students. I guess I don't know them very well.

Interviewer: Do you feel comfortable interacting with the students, being yourself- an attending?

Participant: Oh, yes, in general I do. You know, I have gone on Project Haiti for twenty years, and I spend a week living with the students and you know, I've developed some long-term relationships with some of them that have gone on. You know, **[unintelligible 00:08:58]**. So, yeah. I don't feel uncomfortable with students. It's just that the only time I ever see those students is at the other end of the table, at someone's house or something. I just don't have too much interaction.

Interviewer: One-on-one interaction.

Participant: Yeah.

Interviewer: What about interacting with other physicians?

Participant: Well, I know a lot of people there, you know, that was one of the things that attracted me because right now, you know, being at the VA, and being in a very small group, I don't get to interact very much with other faculty. Basically, the only woman I interact with is NAME.

Interviewer: Really?

Participant: Yeah, it's quite—I'm very, sort of, isolated in a way. You know, we have our offices of the pulmonary people all together on the fifth floor, but when I'm in the ICU, I basically live in there for two weeks, and I don't have so much interaction with, you know, other than the hospital staff and the occasional student we have in the ICU, and the fellows, and of course the pulmonary **[unintelligible 00:10:17]**.

Interviewer: So, this gives you an opportunity to see everyone, is that what you're saying?

Participant: Yeah, the faculty- a lot of I know from before.

Interviewer: Obviously, well you're an idol for all of us. So, what is the benefit of the book club to you? Why do you attend? Some of the questions may be a little redundant.

Participant: I think, well, reading books that I may not have thought about reading, interacting with other people, interesting discussions, you know, socializing.

Interviewer: So, is there an element of self-care?

Participant: Probably, yeah. Certainly something—I guess it's an element of self-care. Something I have to do that's enjoyable.

Interviewer: Does the book club make you read more?

Participant: I would say no.

Interviewer: Okay, because you're already reading a lot? Is that—

Participant: I already read—I like some mysteries and I—a lot of times I like to browse through books at bookstores and find something that catches my interest. And, my daughter has a book club, my daughter in Chicago, and she passes books onto me also and sometimes when I've been in Chicago, I've gone to her book club.

Interviewer: Oh, really?

Participant: Yeah.

Interviewer: What does she do?

Participant: Well, she is a—she works for a nonprofit foundation that teaches property management all over the world, and she was in the Peace Corps, she speaks fluent Russian, so she was recruited because they have a branch in Russia. And now she's in charge of their international division, so she travels—

Interviewer: I see. So, she's not in the medical field.

Participant: No, neither of my daughters—my other daughter is a writer—

Interviewer: Oh, a writer?

Participant: Yeah.

Interviewer: So, kind of runs in your family- so they can recommend books to you too.

Participant: Yeah, so we have a lot of books passing through and then I have some cousins in California that I'm very close with and they mail me books back and forth, so between all of that, I get to read a fair amount.

Interviewer: And then the other question you kind of already answered is are the books that the book clubs some of them you would never have chosen for yourself? I think you said yes, right?

Participant: Yes, I would agree with that.

Interviewer: Yeah, like *The Middlesex* and all of that. Through your medical training, have you ever had some kind of a formal—were you exposed to literature as part of your formal medical training?

Participant: No.

Interviewer: Not at all? Okay.

Participant: We had—well, the only way I can describe is that we did have a whole course on the history of medicine, and there were some fiction related to that. I'm trying to think a book about **[unintelligible 00:14:31]**- I'm trying to remember some of the other—but, not anything where there was ever literature engulfed in a course.

Interviewer: Like a fiction; it was usually—

Participant: I can't remember anything like that.
Interviewer: Okay, so I'm going to stop the recording.

FACULTY 4
Interviewer: Okay, now I'm starting the interview with NAME.

Participant: I don't exactly remember. I think it was through the Narrative Medicine program that NAME led, and I remember we had lunch meetings and I recall we read books and had book discussions as part of that. And I can't for the life of me remember what the first one was because it was so long ago. We did that, and then we moved to students and various houses and I remember going to like student apartments sometimes for it. And again, NAME was much more involved then. And then it seemed like it sort of died out a little bit, or somehow I didn't know what was going on. Then I was hearing that you and Ying and some of y'all were part of it, and then I think finally I just thought, "why don't I ask to get back on the listserv and get back in." So, there was a hiatus there where I wasn't going.

Interviewer: Okay, got it. What is a book that you recall having an impact on you that we read in the book club?

Participant: I recall *When the Spirit Catches You and You Fall Down*. I don't remember if I read it before the book group, but it definitely had an impact on me because it, I thought, did such a nice job really comparing two sides of a complex story and giving each an equal voice where I felt that I could truly understand both perspectives. So, that one I definitely—

Interviewer: Did that have an effect on you in terms of your clinical practice?

Participant: I don't know. I mean I think I'm by nature always a very judgmental, close-minded person, so anything that challenges that and causes me to think of different perspective is good for me. And I think the book we read, *Hunger* by Roxanne Gay, that affected my clinical practice in making me be much more cognizant in my biases about obesity and be much more compassionate I think.

Interviewer: Have any of them affected your personal life?

Participant: I don't think so. Although, I've really liked some of the books. But, I don't think—I've been in several book clubs through my life and I don't recall fiction changing my personal life as much as non-fiction, religious works, and essays, and things like that that get me thinking.

Interviewer: Okay, we'll go to the next question without turning this off, if we can manage it. So, what are your interactions like with members of the book club?

Participant: Does this mean outside the book club?

Interviewer: With members of the book club- I think in the book club or, yeah. In or out.

Participant: What are my interactions? I hope they're collegial and personable. I'm not sure how to—I feel like the people I know in the book club—most of the people I know—

Interviewer: What's the students versus the faculty? Do you feel that your interactions with students is different in the book club than in other settings? I think that's what we're getting at.

Participant: I do think so. I think the students have a much stronger, more equal voice and it's fun to see that. We've had some that have had really strong personalities and opinions and have been much more knowledgeable about the authors in the literature than the physicians, so they're really taking the lead role and it's fun to see that. I like it when they feel like they can do that. I don't think we have anyone right now who's exactly like that—

Interviewer: But like NAME—. And **[unintelligible 00:04:31]** she was sort of that way I think.

Participant: Yeah, and even NAME in his quiet way, which I always felt like we would always stop and really listen to him like what insightful thing he had to say. I feel like the students were often more insightful than the faculty.

Interviewer: They were more insightful.

Participant: And it's nice to have the tables turned like that.

Interviewer: It is. It is, exactly. What is the benefit of the book club for you? Why do you attend?

Participant: I just love to read, and so any excuse to read and get—I like the interaction in context of books, in a quieter setting where you can really to talk. I've never been someone who goes out to bars and sporting events- they're just not a good social thing for me. So, I think it meets this introvert's social needs, and I've also—sometimes there's books that I don't necessarily like, but I get so much more out of the discussion and just come away and I'm just always interested in that and really unpacking ideas and hearing people's views and it's just nice to see people. I just like—the faculty members are just people I like so it's nice to just get a chance to see them and spend time with them.

Interviewer: Great. Does the book club make you read more than you might read anyway?

Participant: Probably not, because I read a lot. Though I forgot, the last question, I would add one other thing I get out of the book club is book recommendations. I always love to hear what people are reading. And I don't always take the recommendation but—

Interviewer: Even if it's not a book that we're going to read again.

Participant: Yeah, I love just that chit chat where we share what we're reading and just fun things that we know wouldn't be as good for the book group. Yeah, I'm not—certainly I've read things that I wouldn't normally have read for the book group, and I like that. And, I'm not like a short story reader, yet we tend to read more short stories, and those are ones that I enjoy talking

about much more than I enjoy reading, so that's an example of how book group changes it, but I read a lot so I'm not sure it changes the amount.

Interviewer: So, other than the short stories are there other types of books or books that you might not have read anyway?

Participant: Well, there's quite a few that I probably would not have read anyways. Not because I was not interested, but just because I have so many piles of books and they just get buried. For example, when NAME recommended *The Land Remembered*, I sort of knew about that book and had it way in the book of my mind and I'm sure I would have never in my life gotten around to reading that book.

Interviewer: Me neither.

Participant: So, there are things like that and then there's ones where sometimes I'll even suggest because I've been meaning to read because that'll push it more to the front. So, I guess in that way it influences the prioritization of some of my reading.

Interviewer: Now when you were in medical school, or any other part of your training, did you ever have any formal discussion of literature?

Participant: I did. I had two very good ones. One is Dr. NAME. NAME, of the **[unintelligible 00:08:14]** group. He offered an elective, I don't think it was for credit, but an elective in literature that was specifically non-medical literature, because Dr. NAME had a master's in literature from Princeton and so he loved discussing literature. So, we read sort of great books.

Interviewer: Cool, I didn't know about that.

Participant: Yeah, we would meet in his—and it was never more than five of us. And we would meet- I don't remember how frequently, if it was monthly- up in his office, just one evening, and his office was in the old part of the hospital and it was one of these, where it had a little lab kind of built in- it was huge, and so it had a little sofa area, his desk, and all these book shelves. And then he had a lab inside it, and he would like make tea and stuff with his little bunsen burner and all that. And then he would always have us for dinner, once a year, and he was just, yeah, he was just a big reader. And he had strong opinions about things and it was a little intimidating, but I absolutely loved it because it was just a chance to read and talk about literature. Back when I was in medical school here I don't think we had nearly the degree of talented, diverse backgrounds we do now; it was much more just a lot of biology majors and sports fans and I felt very 'fish out of water' going to med school here, which I think is very different now. So, that was like a time where it was like, "oh, so there's humanities people, there's a few of us around." So, that was fun. Then, I took a fourth-year elective that NAME ran called "Literature and Dying" and it was a series of—I know we read *The Death of Ivan Ilyich*, I'm sure we read a lot of poetry because NAME loves poetry and I guarantee there was some T. S. Eliot. I'm trying to remember what novels—I found my course guide for that somewhere **[crosstalk 00:10:16]**. So, we would meet at his house and talk about the literature. And then also I remember he and his wife had us all over- it was somehow it fell—it must have been in March because it was a month-long elective,

fell on St. Patrick's Day, and I had never had an actual corn beef and neeps and tatties and the whole Irish meal, and NAME made all that, his wife. And I remember some of my classmates who were in that elective. I pretty much sought out any opportunity—if it said literature, you could sign me up.

Interviewer: Yeah, very interesting. I think that's our last question, but I will double check. Can you compare that experience to our current book club? Those two experiences?

Participant: Our current club is just much less formal and our current club- we don't do as much analysis of the structure and really pulling out the language and things the way those courses did a bit more. And Dr. NAME's course was very specific to not be patient or medicine-related, whereas the Death and Dying was specifically for that. So, the funny thing is I'm not sure it really impacted my views on how I care for dying patients, I just think the time was such that I don't think that really came into mind later on when I was actively learning about those things.

Interviewer: Isn't it interesting that he had that back then? That's so interesting. Anything else you want to say about the book club?

Participant: No.

FACULTY 5

Interviewer: So, just to begin, could you tell me a little bit about your involvement with the book club, how you got involved, and how long you were involved?

Participant: Okay, so I got involved just because my work with medical students and because I had been teaching a class that uses—for medical students, Narrative Medicine for Medical Humanities class since 2002, that focuses on reading and discussing the literature and also watching films and discussing them as well- materials that are related to health and understanding how the humanities enhance health care. And so, and that's basically- I teach and work a lot with literature. So, I was, as you mentioned, I was involved early on and attended a number of the sessions, I also hosted a number of sessions at my house and was involved in selection of books and advising the students who were working on organizing the club for the group.

Interviewer: Do you remember your first book club session? Or any subsequent ones? I mean, do you remember them well enough to talk about them?

Participant: Do I remember what?

Interviewer: Like which first session you attended- what was the first book club session you attended?

Participant: I don't remember.

Interviewer: Do you remember a typical session for you? Could you describe what it was like?

Participant: Well, I—let's see. I don't necessarily know that there was a typical session, I'm just calling to mind some of the sessions that I attended. As I said, there were some at my house, and then I can remember one or two at NAME's house, maybe one at NAME's house, and then a number at different student's houses. I think early on, the number of participants varied widely. So, there were certainly some discussions where they might be maybe two or three or four people—I'm trying to remember what we read. I know *Cutting for Stone* was certainly one of the books that we discussed.

Interviewer: Let me see if I have the book list here. I can pull it up and see if I can jog your memory. Do you remember *Cutting for Stone* because it was a book that particularly stuck out to you as something that was a good discussion or impacted you at all?

Participant: Well, I guess it was a book I had read before—for other purposes and I've actually taught courses on it, so that may be one reason why it stuck out in my mind. Just that I had used the book a lot, kind of maybe independent of the book club.

Interviewer: Okay, and do you remember how the discussions went? What were the discussions like? For example, who took the lead in the discussion? What kind of issues were discussed?

Participant: I would certainly say that it depended on the nature of the book. So, kind of, whether—like I remember *Cutting for Stone* there were some elements of—because it's health-related there were elements of kind of talking about professional identity as well as the material of the book.

Interviewer: So, sometimes the discussions would kind of be both about medicine and about the book itself?

Participant: Mhm. But again, that depends on the book, because obviously not all the books had kind of a health or medicine focus.

Interviewer: I'll read you a couple of items from the books, and I'll see if you remember any of the books that were read- so *Cutting for Stone* obviously, *Major Pettigrew's Last Stand*, *When the Spirit Catches You and You Fall Down*, *Wild*, *Glasshouse, The Brief Wondrous Life of Oscar Wao, Life of Pi, The Boy Who Harnessed the Wind, Devil in the White City, The Room, State of Wonder.*

Participant: [unintelligible 00:04:37]

Interviewer: Yeah, go ahead.

Participant: Well, I remember *The Room, Devil in the White City, Spirit Catches You, Life of Pi.* I just wanted to get those out, because I was losing track of them.

Interviewer: Yeah, sorry. Yeah, I think that that takes us pretty far into the—so, the ones after that are *Animal Vegetable Miracle, Dreams of Joy, Charlatan,* but I think we're kind of a couple years in—do you remember those books?

Participant: *Animal Vegetable Miracle* certainly.

Interviewer: When you think back over those books now, were they all books that you had read before?

Participant: I would say many of them, yes.

Interviewer: And were you ever, as part of the book club, reading stuff that you might not have read on your own or you might not have necessarily chosen for yourself?

Participant: I would say, yes. There was one book- now I forget the author. Could you read the list to me again?

Interviewer: Yeah, sure. I'll go slower this time sorry. *Major Pettigrew's Last Stand*, *When the Spirit Catches You and You Fall Down*, *Wild*, *Glasshouse, The Brief Wondrous Life of Oscar Wao, Life of Pi, The Boy Who Harnessed the Wind, Devil in the White City, The Room, State of Wonder, Animal Vegetable Miracle, Dreams of Joy, Charlatan,* and then *Love in the Time of Cholera, This is How You Lose Her, The Shadow of the Wind*- I don't know if any of those sound familiar.

Participant: *Shadow of the Wind* I remember. *State of Wonder* or *The Boy Who Harnessed the Wind*. And, is *Americanah* on that list?

Interviewer: Yeah, yeah. *Americanah*. Then, there was like a Sherlock Holmes that you'd read. *A Study in Scarlet,* before that was *Ender's Game*, *The Hundred Year Man—The Hundred Year Old Man Who Climbed Out the Window and Disappeared—*

Participant: Oh, yeah. I remember that one.

Interviewer: So, were any of those books, books that stuck out to you as things that impacted you or that you thought about later? I mean I know that some of them you had already read and probably—

Participant: Yeah- I mean certainly—and some them I actually can't remember who suggested them, but *Spirit Catches You and You Fall Down* is one that I've taught, so is *Animal Vegetable Miracle*- I've taught that in several different types of classes. Oh, I guess I was there for the *State of Wonder* discussion. I guess for the most part—and *Americanah* is a book I had read before and really liked, so I guess for me the one's that had the most impact were ones that I had used, or the material was such that I would use them in teaching other classes.

Interviewer: And when you think back to the book club, especially because you're someone who has experience in using literature in fiction and non-fiction in medical education, does

anything stick out to you as ways that the kind of setting of the book club was different that the settings you worked in or had literature in?

Participant: I mean, I guess just it is nice to have an opportunity for students and faculty to get together on more equal footing. I think that's important- to have those opportunities.

Interviewer: And do you remember what the interactions like between students and faculty? I mean, who was leading the discussion? Or if everyone was speaking equally, in terms of amounts of time? Do you remember how that kind of dynamic played out?

Participant: I would say I think everyone was speaking pretty much equally. I think it kind of depended on who had been responsible for suggesting the book. So, on the whole the faculty had probably tended to dominate, but certainly if students had suggested the book or were really enthusiastic about it, then they would be more likely to lead the discussion.

Interviewer: And what was the experience of going to different people's houses for you? So, going to different faculty's houses and going to student's houses and hosting the book club at your house?

Participant: I think it's always fun, always a pleasure to just talk to people in settings that were outside of the more formal and impersonal academic settings.

Interviewer: So, it kind of took it away from the school aspect a little bit.
Participant: Very much so. And again, since I think it's important to have situations where faculty and students are getting together in less formal settings and on more equal footing, I think that that is a great part of the aspect of the project or program.

Interviewer: For you, what do you think the benefit of the book club is? And why did you attend and why did you think it was kind of a good thing to be doing?

Participant: Just, I guess again, because it's- well one, I enjoy discussing books- reading and discussing books and hearing people's perspectives about them. I guess also I enjoy discussing books with colleagues and with students and I really enjoy that informal setting.

Interviewer: What do you think is the main difference for you, if any I guess, between something that is informal like the book club and including literature or fiction or non-fiction as part of a formal curriculum?

Participant: Well, I guess obviously the level of formality and this varies to kind of how much—what specific message I might want a student to get out of a book varies on what the class is, because some of the classes I teach are more formal than others. But, so I guess one thing I like is just the—it's more open-ended which is nice. On the other hand, some books that I teach like *The Spirit Catches You and You Fall Down* or *Cutting for Stone* or *Animal Vegetable Miracle*, we take a whole semester to talk about a book, so obviously in that case you can get more into depth into a particular book, so it's kind of a tradeoff. If you read one book and discuss it for one hour, there's only so much that you have time to say about it or so much time that you

have to explore the details of that book. So, I guess that's kind of a little bit of a drawback for any type of a book club where you're talking about one book just for an hour. But, it's nice also to get an introduction to a range of material. So, I guess I would say obviously there is a tradeoff.

Interviewer: And this is going to be something I know that you feel very passionate about, but can you talk about what you think the benefits of having people who are undergoing medical training be reading literature.

Participant: Well, it helps them understand the human experience- what it means to be human. And obviously it depends on the type of literature that you're reading, I guess the broadest level- just being familiar with literature and the human side of life is important. It also can function as a way of relaxing, as a way of reducing stress. If you're reading and not focusing on studying, so taking a break from reading textbooks and studying is important for student wellness.

Interviewer: And do you feel like, I don't know if this is going to come out as a coherent question or not because I'm just thinking off the top of my head, but do you feel like there's a draw for the book club for students who read for pleasure? Do you find that is a majority of students in medical school are reading for pleasure? Do you feel like the book club functions also kind of for people who really enjoy it? What's your experience with medical students and getting them to read literature? Do you find that they're overall as a group equally receptive, or some more than others?

Participant: So, I would say that most medical students feel guilty about reading for pleasure. They feel like any extra time that they have should be focused on studying, and so for them maybe having permission, i.e. knowing that their faculty will be there and support their activities allows them to take the time to read for pleasure.

Interviewer: Okay, well I really appreciate your time, that is

FACULTY 6

Interviewer: Alright, so we were talking about the first meeting that you went to and how it was at a student's apartment and I asked if—

Participant: Well, I don't know for sure if that was the first meeting.

Interviewer: Well, the first one that you remember.

Participant: The first one could have been at Dr. NAME's or Dr. NAME's. Yeah, I just don't remember. That's just one of the ones that I do remember.

Interviewer: Sure, and what was the composition of the book club like when you started to go? Who all was going, in terms of students and faculty?

Participant: Yeah, there was sort of a mix of students and faculty. I don't really remember exactly how many.

Interviewer: Were the students and faculty students and faculty that you already knew, or did you meet them through book club?

Participant: I would say that most of them I knew already.

Interviewer: So, now we're going to talk a little bit about the books that we've read. Looking at that list, is there a book that you remember having an impact on you on that list? Or a book that you remember really strongly that you read with the book club?

Participant: Yeah, there are several, I would just say I'm actually still reading *The Middlesex*, so I can't comment on that one. *Exit West* was interesting. *Land Remembered.* I mean those were—*The Life of Pi*, *The Brief and Wondrous Life of Oscar Wao, When the Spirit Catches You and You Fall Down, The Boy Who Harnessed the Wind, Devil in the White City, The Room*- that was amazing.

Interviewer: Does anyone really jump out at you as—

Participant: I'll just say for this list, I think *Exit West*, I found particularly interesting, I think because of the cultural aspects and the family, what they gained and what they lost, and a lot of the unknowns. I thought that was a fairly amazing book and, so.

Interviewer: Do you feel like any of the books that you've read have had an impact on your professional life? Perhaps changing the way that you think about patients or interact with patients?

Participant: I think they all leave an impact on me. I think fiction sometimes will do that for you, especially when you have the opportunity to speak with others, because I think it just broadens your perspectives and your dialogue. So, I really think that any book that I read has an impact on how I approach life, people, and patients.

Interviewer: Yeah. So, kind of like just reading fiction in general broadens your mental horizons.

Participant: Yeah. Fiction, non-fiction, music. Yeah, just about anything.

Interviewer: Is there any example that you can think of a specific impact or a specific interaction that you remember thinking of one of the books.

Participant: Not really. I think that—I really do think that they stay with me, but I don't think that there's any specific one that made me change anything drastically. I think it gives me just a better perspective.

Interviewer: And what about on your personal life? What do you think the impact of reading fiction is on your personal life?

Participant: I think it nurtures the part of me that loves the humanities.

Interviewer: And why is that important?

Participant: Because it is—it's something that, since I was probably a child, you know as I've said I've always loved books, and I think I was very fortunate through school, both grade school, high school, and college, but particularly in college, to have a really wonderful liberal arts education. And my Freshman Year Studies course, that was the name of the course, was particularly impactful and remains so even to this day. I still actually have some of the textbooks from that course.

Interviewer: Wow, that sounds like a good class. What was it like?

Participant: What was it like? Yeah, it was pretty phenomenal. Very different than what I've heard a lot of others have had the opportunity to experience. It went through—it was a full year; all the freshmen were required to take the course. It went through the Greek and Roman periods, so basically Western civilization- the Greek and Roman period to current time. And it integrated literature, philosophy, music, architecture, science- gosh what else.

Interviewer: So, like a humanities crash course.

Participant: —history. Yeah, all of the exams were essay, so it really required synthesis of all of that, so it was pretty phenomenal, and I think that it still has an impact on me.

Interviewer: Yeah, that's what I was going to ask. When you read fiction now, you still kind of place it in this larger framework?

Participant: Yeah, yeah. And I think that it's important for me, you know, when I get away from it for a period of time I really miss that part—I mean it's probably the same reason I started taking piano lessons again. So, because it's something that's really important, and I probably don't do as well in life in general unless I have some access to the humanities, the arts, and so forth.

Interviewer: Right. So, now let's talk about your interactions with members of the book club. What are your interactions with them at the book club and also outside of the club?

Participant: I have to say from the past, because I really haven't been able to participate most recently. I think it helps people bond, because I think in medicine people—for the most part, physicians tend to not talk about things other than medicine. They forget to talk about the things they love outside of medicine, there's almost an unspoken "you're not supposed to talk about"—or "you're not supposed to have a life outside of medicine." So, I think that the book club nurtures that and helps people step outside medicine. Although, I do find that sometimes it reverts back to medicine.

Interviewer: So, the books that are more medically-oriented, maybe not, but. What about your interactions with students through the book club? Does anything stick out to you in terms of those interactions?

Participant: I love to see the students having this part of their life. I think it's a good start as a student, I think it helps them probably through the rest of their career. I think there's a bonding, again, both with the faculty and students. But, I do love to see them come to the book club.

Interviewer: And do you feel like that seeing them at the book club and interacting with them at the book club is different from interactions you have with students as part of your role as a teacher, as a—

Participant: No, I don't think so. I try to encourage that whether it's at the book club or not, and I just love interacting with students in general. So, I don't feel like it makes a difference.

Interviewer: And do you feel like any of the relationships you had with students through the book club are relationships that have carried through to outside the book club and beyond or is it just the students you see at the book club are students you see at the book club. I guess I'm thinking in terms of mentorship, relationships or building stronger connections in terms of, you know.

Participant: There might be some of that. It's hard to remember. I just interacted with so many students along the way. So, I mean it's possible there was a kind of interaction and something beyond, but I'm not positive about that.

Interviewer: Okay. How about your interactions with the faculty members or other physicians who attend the book club? What are those relationships like?

Participant: I think it helps you get to know them beyond medicine.

Interviewer: Are the other faculty members people that you socialize with outside of the club, or people that you work with clinically?

Participant: Most of them—a lot of them are internal medicine folks, so there isn't anybody from my division, other than myself, that goes to these. So, I kind of interact with others from other divisions, particularly Internal Medicine. And I certainly have a relationship with them through some of the patients we have and share.

Interviewer: Do you feel like that relationship is strengthened by knowing them through the book club? Do you feel like it's impacted at all or do you feel like they're pretty separate relationships?

Participant: I think it's probably helped along the way. I mean, you just get to know each other better. There are other avenues to do that as well, because a lot of us are in Medical Education, there's probably a strong relationship that is built that way as well.

Interviewer: So, you also see them a lot through Medical Education?

Participant: Some of them.

Interviewer: So, kind of speaking broadly, what do you think the benefit of the book club is to you? Why did you attend? Or do you attend?

Participant: I think it goes back to the fact that I love to read, and I love the humanities and I think it's important to keep that in my life as a physician.

Interviewer: What about kind of why a book club as opposed to just reading independently?

Participant: I think it's good to have an opportunity to discuss books, I think that you get some insight that you may not have thought of and there's a nice dialogue, kind of expands your view. It's good to talk about literature.

Interviewer: If you weren't attending to the book club, do you feel like the book club makes you read more?

Participant: No. Not necessarily.

Interviewer: So, you would be reading anyway?

Participant: I would be reading anyway.

Interviewer: And do you feel like there's an element of self-care to the book club for you?

Participant: Yes.

Interviewer: You kind of talked about that- about how important it is to read fiction. But, apart from the self-care of reading, do you think the book club itself adds to that self-care?

Participant: I think it's the reading and the dialogue.

Interviewer: Are the books that the book club chooses books that you would choose for yourself?

Participant: Some of them. Some of them I wasn't, and you know I was fortunately introduced to. But, a lot of them I knew I would pick up.

Interviewer: For the most part, the books in the book club were books you were interesting in reading anyway. Have you read books through the book club that aren't things that you normally would have read, or you even didn't like?

Participant: Let's see. Well, I actually—I think out of the books I've read along the way I think I've enjoyed, even the ones that I did not, I would not have picked up myself, basically the book club introduced me to them, you know, I've been able to get something out of it. So, I think I've been fortunate with the books that were chosen.

Interviewer: Have you ever been exposed to literature as part of your formal medical training? So, as part of medical school or—

Participant: Yes.

Interviewer: And what was that like?

Participant: When I went to medical school, they actually when we graduated they gave us a book— the tradition was to give a book upon graduation that had nothing to do with medicine. So, but they actually gave us something that was medically-relevant, when I graduated. But, that was sort of the tradition. They encouraged to read something every day that was non-medical. And so, I kind of pass that on to students as well. Even if it's just five minutes, to read something that is not directly medicine.

Interviewer: So, was that experience in medical school similar to book club? I mean, did you guys discuss the book that was given or was it just kind of a gift?

Participant: Well, yeah. Getting back to right at the beginning, I purchased a—I traveled a couple of hours, about an hour and a half, every few months while I was in medical school to join up with some friends of mine in Chapel Hill. It was a formal book club, and we read several books.

Interviewer: Was it a medical school book club, or were these friends that weren't in book club?

Participant: No. Some of them weren't in med—it was kind of inter-professional and then there was one that was a professor in English. It was kind of a mix and it was fabulous. So, we'd usually join up the Carolina coffee shop.

Interviewer: And how did you find that book club differed from the one here? I mean, what was the biggest difference?

Participant: That one did not have— it wasn't all medicine. Most of the **[00:15:12]** that are here are, you know, in medicine.

Interviewer: And how did that change the experience of the club itself, I mean, do you feel like it was—

Participant: I think the one when I was in medical school was—we had some really interesting perspectives, outside of medicine too.

Interviewer: So, maybe a little bit more diverse perspectives.

Participant: Yeah, I think so.

Interviewer: Okay, well I appreciate your time, that was all the questions that I had. I appreciate you going through it with me, we're doing this with everybody we can get our hands—

FACULTY 7

Interviewer: Testing. We're live. So, how did you get involved in the book club?

Participant: I think by talking to one of my colleagues, who is part of the book club and then they got me involved. And I've always liked reading books, so I was excited when I heard about it.

Interviewer: Okay. Do you remember your first meeting?

Participant: No.

Interviewer: Do you remember one of the early meetings?

Participant: One of the earlier meetings? Depending on how early it is. The earliest memory actually was *Life of Pi.* Yeah, it left a deep impression on me.

Interviewer: Why did it leave a deep impression?

Participant: Because it is unlike anything I ever read. It's just such an unusual book, and even after you finish it reading you just keep thinking about it. And then in your daily life, periodically, the analogy kind of comes up. Yeah.

Interviewer: So, that's a book you remember having an impact. Any other books you remember having an impact on you?

Participant: In what ways?

Interviewer: In your professional life?

Participant: In my professional life. Yeah, the one that was *Imagine Me Gone*, that one. One of the characters in the book has severe depression and it talked about how the genetic factor effects—such a strong factor in terms of depression as a disease and also, exactly how it feels like when you're truly depressed. And I've always wondered how people ended up committing suicide. It vividly described his whole process, like how he went from really depressed and then in the end he was in the woods and he was, I can find those specific paragraphs later, I believe he cut his wrist and then the blood comes out, it's almost like a complete relief. He felt so light and he felt so good. That impacted me. And it makes me better understand my depression patients. And the other one that impacted my patient care was the obesity book.

Interviewer: *Hunger.*

Participant: *Hunger*, yeah. That has also, you know, made me realize a lot of the things that I—I can see myself in the shoes of an obese person better than I did before, and I realized I had a lot of misconceptions of obese people, like how they feel, what they like, what do they really need from friends and families or doctors.

Interviewer: What did you find that they—how did it change—

Participant: I think first of all, as a doctor, obese people are first looking for sympathy and understanding, instead of a condescending way to teach them knowledges they already know, it's just how difficult it is to actually really do what you know you're supposed to do. And I learned that it's sometimes not always a good idea to mention their weight every visit they come because clearly the character dreaded going to the doctor, because she knew the doctor is going to bring that up. So, even if she had illness, sometimes she just didn't go. So, that kind of struck me. I feel like I should treat them as patient first, and then as an obese patient. Sometimes we get so overwhelmed with this 300-pound patient sitting in front of us and we see them having diabetes and high cholesterol, heart diseases, and we're so worried that's the only thing like we want to fix. And then we read a number of African-American books that also made me understand. Like, the recent book that we're going to read, *American Marriage*, yeah. It made me understand what a prison life is and what a family was like when somebody goes to prison. I never knew like—there was something that really struck me, when he said there are so many women, they schedule their life around visiting their husband at the prison. They get up at 4 o'clock in the morning, they get on the bus, and they go on for two hours. I mean not every day, maybe once weekly, they would go to the prison to visit their husband. It's like their routine. They work it into their weekly schedule to do that. So, all of these things.

Interviewer: Yeah, that's a lot. What about in terms of your personal life?

Participant: I think it greatly enriched my personal life. I become a better reader because I'm more widely read, and you know like if one of the book club members hadn't mentioned "Good Reads" I wouldn't have known—

Interviewer: The website, "Good Reads."

Participant: Yeah. That I could actually use to track my books and then get recommendations from other people, and it was very useful. Yes. And then personally, it also helped me in making more friends.

Interviewer: What are your interactions like with members of the book club?

Participant: Very positive. And very good.

Interviewer: How about with the students?

Participant: With the students? In the beginning I was a little apprehensive about having students in the book club, because I feel like "oh, I wouldn't be able to say certain things I wanted to say. You know, because there are students there I have to watch myself." But, it ended up I didn't feel that way at all. I don't feel restricted, like there—I feel like when we're in the book club even though professionally they are not as mature as I am, but in terms of reading books and understanding books I think they are completely equal, sometimes if not better than me. So, I felt that I have a lot to learn from the medical students.

Interviewer: How about the other physicians and faculty?

Participant: The other physicians, I'm so grateful for this opportunity because we're so busy with our daily grind of seeing patients, so this gives me opportunities to see colleagues outside of work environment. And then we talk about things outside of the book too, like where to buy the best pastry, what are the shows to see. So, you connect more on a personal level, than just a professional level like before.

Interviewer: Well, what are the benefits of the book club- you've spoken about some of them. Why do you attend? Is there an element of self-care?

Participant: Yes. Yeah, I have to say I have never been to a book club that I don't walk out feeling happier and better than I was before I walked in.

Interviewer: Why do you think that is?

Participant: I think it's just—I think everybody wants—I don't want to say **[unintelligible 00:09:36]**. You know like if you have a hobby, like if you're a stamp collector, when you talk to other stamp collectors you feel truly gratified, like they truly understand you. And this is like you're talking to people who have similar interests as you. Also, I always feel very gratified. Whenever, I feel like I learn something. So, yeah. That's probably mainly…

Interviewer: Does the book club make you read more? I think you said that's one of the things you liked about it.

Participant: Definitely.

Interviewer: Why do you think that's important?

Participant: I think it's important because, number one, I want to be a well-read person. Number two, I think reading is such a human existence that is different from any other animals and species. It's a big part of human existence. You know, with books you can basically talk to people who already died, you know. You read these books and you can never talk to like—

Interviewer: [unintelligible 00:11:10]

Participant: Exactly, or the Bronte sisters. You know, they're all in the past generation, but reading their books and you actually—it's like you're having a conversation with them and you understand them better, yeah.

Interviewer: Interesting. Are the books that the book club reads books that you'd choose for yourself?

Participant: Some are, but mostly probably not.

Interviewer: Are they different in some particular way or?

Participant: I tend to read books—I tend to pick books, like some books in the book club, like *The Wondrous Life of Oscar Wao*, I wouldn't have picked that one book. I think I tend to pick just certain books that I usually read, but the book club really has broadened my horizon of reading different books. Like, there's another book, the short story about the Soviet.

Interviewer: Yeah, yeah. I think I—

Participant: We can fill that in. Yeah, like I would never have picked that book. I would have picked *Leila* and those types of books, Or *Kitteridge*, or what was the other one.

Interviewer: You're saying, you would have, or you wouldn't have?

Participant: I would have picked some of those anyways, but the *Oscar Wao*, that I wouldn't have picked. I certainly wouldn't have picked *Buried Giant*. You know, there are some genres I never would have picked.

Interviewer: Did you have any—were you exposed to literature as part of your formal medical training?

Participant: No. Nothing.

Interviewer: Okay. We need a better last question.

Student 1
Interviewer: Great. So first I'll just say how are you?

Participant: I'm doing well, yeah.

Interviewer: Good, good. I'm so happy that you're still part of UF.

Participant: Yeah, I know. It's weird, my position at the **[unintelligible 00:00:17]** is actually UF- Gainesville, and then I do one day a week at UF- Jacksonville on their consult service, so I work with both departments even.

Interviewer: Oh, interesting. Interesting. Okay, so the first question is how did you get involved with the book club?

Participant: Yes, I don't remember. I just remember— I think there was maybe an e-mail to see if people were interested or my friend NAME might have invited me.

Interviewer: NAME- we were trying to dig out students and I don't think we dug her out. You don't happen to have her contact information, do you?

Participant: I do, yeah. I can send it to you, I'll have to pull it up but yeah. I'm pretty sure she invited me because **[unintelligible name 00:01:09]** girlfriend had a book club and we tried that and it fizzled out and I think this one branched off from that maybe.

Interviewer: Oh, that's interesting. So, there was another medical student book club? So, you were a part of that? Or she was?

Participant: Well, it was a non— there were some students and some non-students. Unless that was this book club, we met at somebody's apartment and there weren't any faculty at that one.

Interviewer: Interesting. Do you remember your first meeting?

Participant: It was at a faculty's house, and I can't remember if it was your house or Dr. NAME's house.

Interviewer: I remember you being at both. So, I don't remember which came first either.

Participant: I think it might have been Dr. NAME's house because I had never met her.

Interviewer: Didn't you bring your baby, or was that later?

Participant: I might have, yeah. I definitely did at some of them. I'm looking because— yeah she was born in 2009 and I think the first one— that first book was what I went to first.

Interviewer: Okay, would you remember what book that was?

Participant: Well, I guess I'm looking— I missed these bottom ones.

Interviewer: Yeah, and we've thought of some other ones we had forgotten.

Participant: Yeah, and I had one that I was like "oh that one's not on the list"

Interviewer: What was that?

Participant: It's at my house but I can't remember—

Interviewer: Okay—

Participant: I'll try to dig it up.

Interviewer: That's okay. So, what's a book that you read through the book club that you remember having an impact on you?

Participant: I liked them all— an impact on me, looking at the list. I mean definitely "The Glass House." I think that's really relevant to my work too.

Interviewer: Okay, yeah. So that was the one— because she was a—

Participant: Her parents were homeless—

Interviewer: They were homeless, and her parents were mentally ill in some way.

Participant: Mhm, yeah.

Interviewer: Yeah. You know I still have your copy of that book I think.

Participant: Really?

Interviewer: Yes!

Participant: Oh, that's funny I didn't know—

Interviewer: Do you remember? I think you left it at my house and I kept trying to give it to you. I should send it to you! When you— if you think of the Jamie Johnson's e-mail if you wouldn't mind also e-mailing me your address I'll send it to you.

Participant: Okay!

Interviewer: It's the least I can do for you participating in this interview.

Participant: Okay, cool.

Interviewer: Okay, interesting so that had an effect on your professional— did it have an effect on your professional life or how you interact with patients?

Participant: Yeah, I think more my personal life— that kind of opened up my views that not everyone, you know— hat people can be average and be homeless.

Interviewer: Oh, okay.

Participant: And then, good things can still come out of homelessness.

Interviewer: There can be some hope.

Participant: Yeah, and so it's not that everyone— you know that like all the children were experiencing homelessness and they didn't particularly have mental illness or addiction or anything.

Interviewer: Right, right. Now were you working with the homeless at that time?

Participant: I probably would have rotated through **[unintelligible 00:04:52]** by then. Yeah, I don't know— do you think this started in 2010 or do you think it started earlier than that?

Interviewer: Oh, the book club— I think it was earlier. Was that when we had—

Participant: Yeah, because I would have rotated in 2008 I think.

Interviewer: Okay, yeah. Did you have your baby in your fourth year?

Participant: Yeah, in 2009.

Interviewer: That was your first child— I'm trying to remember.

Participant: Yeah, yeah.

Interviewer: And now how many do you have?

Participant: Now I have four.

Interviewer: That's wonderful. Okay, and so any other books you remember having an impact on you?

Participant: Oh gosh, a lot of them, let's see— I remember "The Devil in the Light City" was really good and I was surprised because I don't like, you know, scary things and I just remember it being really interesting. And that that could really happen in real life. Let's see, what else is— the one I, the one I can't remember the name of, I think that one impacted me a lot. I'll think of the name and send it to you. But, it was basically one girl— it was like sisters and one was like really— she had some sort of, maybe some sort of growth hormone thing where she was very large and not very beautiful and just kind of what her life was like with that. And I think her sister was pretty and got married and something like that.

Interviewer: Oh, I don't think I read that, that doesn't sound familiar, wow. But, I might have missed that. Yeah, interesting.

Participant: It was less about the growth hormone thing, I just remember because we were learning about it at the time.

Participant's Child: Who is that?

Participant: Someone from work.

Participant's Child: Can I say hi?

Participant: You want to say hi? Can my daughter NAME say hello?

Interviewer: Oh, please! Hi, NAME!

Participant's Child: Hi.

Interviewer: Thanks for letting your mom help out

Participant: Yeah, we're talking about books that I read. Okay, go play. Okay, alright.

Interviewer: What do you remember about your interactions with members of the book club?

Participant: Oh, I just thought it was really cool to have attendings there reading books too, and people from all different years. I got to meet people I never would have met before, which is important in med school because you can kind of get isolated in med school. And just to see— just to have something that was outside of studying all the time, that was also really cool.

Interviewer: So, yeah, you mentioned interactions with other students and with with the faculty— yeah. So, why do you think you continued to attend? What were the benefits that you got from it?

Participant: I think having an outlet outside of studying, and just having kind of— it wasn't really accountability but it kind of was. Like, "I'm allowed to read this book because everyone else is," you know? "I'm going to take time to do this because other people are doing it too." **[To Child]** Put them on the ground.

Interviewer: I seem to remember you once told me that you read a lot of the books even if you didn't come.

Participant: Yes, yeah I did. I tried to read a lot of them, but when I look at the list I don't remember all of them. I don't think I read all of them, but it did— I like to read so it gave me— I have a hard time picking books, so I would just use that as my choice for books.

Interviewer: Yeah. Do you think that the books that you read in the book club were books you would have chosen yourself?

Participant: No, no.

Interviewer: How were they different?

Participant: They were just a wider range, a much wider range. You know, like the one that was scary, I never would have picked that. I usually like shorter stories, so some of the longer ones I might not have chosen right away.

Interviewer: Do you think it made you read more books than you might have read otherwise? Or would you have read something else, do you think?

Participant: Yeah, I think it made me read more than I would have otherwise. Because a lot of the times I would get the list and be like "oh, I got to get that book!" You know, I wasn't looking for books to read. It was just that when that list would show up, I would get it.

Interviewer: Was there an element of self-care in your being a part of the book club or reading the books?

Participant: Yeah, yeah I think so. Just, like I said, getting outside of— taking a break from other things, taking a break from studying. I think it was interesting because I guess I assumed the book club would be all— you know, I took Narrative Medicine, so I kind of assumed the books would be related to medicine, and so I found it surprising but good when they were not about medicine.

Interviewer: So, you did take the Narrative Medicine class?

Participant: Yeah.

Interviewer: And what was that like?

Participant: That was really good— that was my second year, and again I got an excuse to do something fun and enjoyable.

Interviewer: So, that was a little different than the book club, you were saying, because it was more related to medicine?

Participant: Yeah, yeah.

Interviewer: Were you exposed to literature as part of your medical training at any other time?

Participant: I would— I mean, during medical school or after?

Interviewer: Yeah, or residency or fellowship or whatever.

Participant: Yeah, I would ask— I think in my second year we tried to compile a list, our program, of recommended reading books, and that was more related to psychiatry, which I tried to organize the department to have a list of things related, but they could also be fiction.

Interviewer: Was that something people were able to keep up?

Participant: Say that again.

Interviewer: Was that something people were able to keep up in your department?

Participant: No, I think that was a one time— we made a list, and we just kind of passed that along.

Interviewer: I see, so you were kind of like inspiring others, it sounds like.

Participant: Yeah, and then once I did find some really good books I actually got the author to come give grand rounds, and that was really cool.

Interviewer: Now, what was that about— who was that?

Participant: That was NAME— actually she spoke at a Gold Humanism conference that I went to and then she's a psychiatrist and wrote a short little book about interesting cases and then when I read her book, after her talk, then I was really inspired and that's when we got her to come down— that's at the time that I was an attending though.

Interviewer: Yeah, interesting. Interesting. Gosh, is there anything else you recall about the book club or would like to share?

Participant: No, just that I don't get the e-mails anymore, do they still do it?

Interviewer: Yes! So, you're not on the list anymore? And you still have your same address, right?

Participant: Yeah.

Interviewer: I will definitely make sure you're back on the list.

Participant: Okay, cool.

Interviewer: NAME does the list and I will make sure that you get back on there. Yeah, I love thinking of you out there, wherever you are, still reading the books even if you're not there.

Participant: Yeah, and actually I would— you know at some point Jamie Johnson, when we were in residency, she lost her UF e-mail and so I would tell her "oh, this is the next book."

Interviewer: Oh, so you were sharing with her too. Oh, that's cool— we should definitely try and get in touch with her. Thank you. Hey, thank you so much for taking time out of your busy life to talk to me Ana.

Participant: Of course, and NAME, she'll remember way more details. She'll probably tell you the whole list of books even, so yeah.

Interviewer: Oh good! Good! Okay, well we're looking forward to getting in touch with her.

Participant: Okay.

Interviewer: Okay, take care and thanks again Ana.

Participant: Yeah, thank you.

Interviewer: Bye-bye.

Participant: Okay, bye-bye.

STUDENT 2

Interviewer: Alright, NAME, I'm going to ask you questions— okay NAME I'm going to ask you questions. First question: how did you get involved with the book club? Do you remember your first meeting? What was it like?

Participant: I remember that I got an e-mail in medical school that was sent out to our class, about the next book club meeting and I just thought, "what a nice thing it is to do in med school- to be a part of a book club." I'd never been a part of a book club before and I really haven't read much for pleasure since college, so I thought it'd be a good excuse to pick up a book and see how it goes. So, I remember when I showed up for the first one that it was just really chill, and it was fun, and people were nice. There was food and we just sat around, and we just said whatever we wanted, and I felt like I had good ideas sometimes to contribute, so it was just a really fun and overall good feeling type of thing to be involved with.

Interviewer: What's a book that you remember having an impact on you? What impact?

Participant: Do I go by A and B? So, I can just—

Interviewer: Yeah, sure- and professionally.

Participant: So, a book that I would say has impacted my professional life is "The Vegetarian," which at first, to me, I thought it was a pretty strange book. It was just a kind of wacky fiction about this person who has this really terrible relationship and really strange eating habits, but then at the end I felt like it was such a twist to find out that all of this was a— not only an eating disorder, but a psychological disorder that all stem from these things that she witnessed when she was little and it really just reminded me that sometimes when patients **[unintelligible 00:02:16]** do things I can't understand or behave in ways I can't understand, that I really— I do not know what they've been through and what they've seen and what they've done in their lives that have led them up to this, and it just really reminds that I really don't know the whole picture and really to not be so quick to judge.

Interviewer: How about your personal life?

Participant: So, I'd say a book that affected my personal life is "A Land Remembered," which was about Florida. So, after that I got pretty interested in Florida history, especially local Florida history, and I've been reading up a little bit about kind of the history and story of this area. So, for example, Payne's Prairie, which I always assumed was named after some white settler, is actually named after King Payne, who is one of the chiefs of the Seminoles during the Second Seminole War, and that the Seminoles were based out there on Payne's Prairie in Micanopy, during that there were some significant battles and I just thought that was really cool and it's one of the things that I consider a hobby and something to take my mind off of work and medicine and just to learn a little bit about the history of this area and to appreciate what we have here.

Interviewer: What is the benefit of book club for you?

Participant: The benefit of book club, one: is to really encourage me to read. Like I said before, I really haven't read very much until book club, since college, that was not related to work. I just already felt like I was already doing so much reading that I couldn't get a lot more in. But, that turned out to be completely untrue, and since I've joined book club I've not only read these books but just really sparked reading so many more, and it helped that I discovered audiobooks when I first joined book club because I just can't— would not have imagined the number of books I read since then, if I was looking from before I joined book club. And another one is also to really make a lot of good friends and just have a good time during the meetings. And it's interesting because, my year, all of the students, when I was a fourth year medical student going to book club, all of the students that went regularly turned out to be internal medicine residents, so I wonder if that partly could have contributed to my choosing of internal medicine because so many people in book club were internal medicine, and they were people I looked up to and had the type of life that I wanted.

Interviewer: Is there an element of self-care?

Participant: Definitely. So, besides just the relaxed atmosphere and the fun atmosphere of the book club meetings themselves, this has really gotten me back into reading and along with that, back into listening more to the news and to podcasts and I think it's really helped me relax. It's just something that is both, I feel like, informative and educational for me, but also fun and relaxing to do when not at work.

Interviewer: I don't know what happened. It kind of got stuck. It left. Does this book club make you read more?

Participant: Definitely. I think I answered in some of the previous questions, but besides reading the books that were suggested each time, I ended up discovering so many more books, not only through recommendations of book club members but because just, in general, now I'm more interested in reading and I'm able to make more time for reading and audiobooks.

Interviewer: Why is it important?

Participant: Well, it's important because I feel like it's one good way to get perspective on things I otherwise wouldn't have gotten a perspective on and also just to have some common

ground and common language with other people I meet, including patients. And also, it is relaxing just to do as a hobby.

Interviewer: Are the books that the book club picks, you wouldn't have done so yourself?

Participant: I do not think I would have read any of these books that I read with the book club if I had picked them myself, so I'm really glad that these were assigned to me because I loved all these books. Well, I would say I found all of these books really interesting and I'm glad I read them, and I wouldn't have read them otherwise.

Interviewer: Have you ever been exposed to literature as part of your formal medical training?

Participant: So, I've never been assigned a book to read during medical school, that I can think of. We have certainly have been given lots of articles and excerpts and things like that, that were interesting. I did end up, by myself, reading a lot of medically themed books during medical school, quite a few things by **[unintelligible 00:07:58]** and also things like "Emperor of all Maladies" and "Biography of Cancer" and "When Breath Becomes Air" and that type of thing, which I think all contributed to my sense of the profession and also to get some good perspectives and sometimes even clinical pearls, which may or may not be the intention of those books, but I wouldn't say that any of those were part of my professional medical school training. They weren't assigned to me.

Interviewer: How is that experience similar or different than this book club?

Participant: So, those were books that I read on my own and I found interesting, but then I have less of a format to discuss them. Sometimes I meet other people who have read similar books and are willing to discuss them, but it's nice that book club has a formal setting where we can all sit down and talk about a book that we all read together.

Interviewer: Okay, thank you.

Participant: Thank you.

STUDENT 3

Interviewer: So, the first question is, how did you get involved with the book club? Do you remember your first meeting and what was it like?

Participant: I do remember my first meeting. I got involved because an e-mail went out to the listserv, and so I RSVP'd yes, and it was at Dr. NAME's house and I think the book was— I thought it was "Animal Vegetable Mineral," but I see that listed here as May 2012, a lot longer after. But, I do remember it was at Dr. NAME's house. I remember it was mostly women, and I remember it was people from all different classes, and some of them brought their babies.

Interviewer: Oh, okay. Alright. And, what was it like? What word would you use for— to describe the meeting?

Participant: Yeah, I would describe it as welcoming and inviting.

Interviewer: Okay, and what is the book that you remember having an impact on you, and what impact? First from a professional standpoint, like on patient care and that kind of thing.

Participant: For the patient standpoint, I think one of the books that spoke to me the most was **[unintelligible 00:01:35]**. I think just because I don't have much connection with old married couples and I don't have anything personal to relate to them and I feel like this book so intricately illustrated the inner-workings of a marriage and a relationship between an older couple, not that I would give you any insight to any particular couple's relationship, but I just thought maybe it put some of their issues into light.

Interviewer: Wow, yeah so true. I didn't think about that. Anything else from a professional life standpoint? Any books?

Participant: I think that's probably the one that is the most—

Interviewer: Alright, that's good. I was just wondering. What about personal life?

Participant: Personal life, I think that "The Hundred Year Old Man" was probably one of my favorite books I've ever read because it was just so funny so—

Interviewer: I read it after you recommended it to me.

Participant: It introduced me to this book that I don't think I would have ever picked up otherwise, but it became one of my personal favorites. So that was—

Interviewer: But, how does it improve your personal life? How does it impact your personal life?

Participant: It just makes me happy to think about it, it just brought joy to me. Probably "Animal Vegetable Miracle," I think of her and now have a very large garden in my backyard.

Interviewer: Animal— hold on, hold on. Animal Vegetable—

Participant: Miracle.

Interviewer: Miracle, okay.

Participant: I think it's **[unintelligible 00:03:23]** as well.

Interviewer: Okay, is that one on there, or no? Oh, that one is.

Participant: We had listed wrong, it's called on the list—

Interviewer: Mineral— yeah, yeah. Okay, got it. Okay, and because of that you ended up having a vegetable garden, did you say?

Participant: [unintelligible 00:03:46]

Interviewer: Somehow, you're breaking up a little bit.

Participant: Is this better?

Interviewer: Yeah, now it's better

Participant: Okay, no I was saying I have a vegetable garden and when I'm out there I **[unintelligible 00:04:05]**.

Interviewer: Okay. Okay. What are your interactions with the members with the book club? How was your interaction with fellow students?

Participant: I think it was probably one of my favorite interactions because it brought together students from different classes. So, it introduced me to people, like as a first year medical student I'd seen these people walk down the halls but I had no idea who they are or any reason to, then I was like "oh, this is NAME" or "oh, this is NAME" and then you go on rotations, or like I was on my Peds rotation and was like "oh, now NAME is my attending," but we went to book club together as medical students. But, I think just bridging connections across different classes was what I valued most in those interactions.

Interviewer: What about interactions with physicians and other faculty?

Participant: Again, I think it allowed us to interact in a non-clinical way that kind of strengthened our relationship inside the hospitals, I mean obviously like working with you and then working with you in clinic and if I had never done book club then I would have just showed up to your clinic as a bright-eyed third you who you couldn't differentiate from anyone else. Same with Dr. NAME, she was my portfolio director when I was a third year on the medicine clerkship and having, you know, knowing that. So, just being able to go into these rotations already having some sort of relationship with the faculty I thought enriched my clinical rotations then.

Interviewer: Great. Any other thoughts on this particular question before we move on to the next?

Participant: I valued having the pharmacists there also since you generally almost never interact with the pharmacists in clinic.

Interviewer: What's the benefit of the book club for you? Why do you attend? It's kind of a little repetitious of the previous questions.

Participant: Yeah, I think, I mean book club— well I don't think it's repetitious— in general it's just like another way of keeping your sanity and focusing on your wellness and the things that make you happy. Obviously if you hated reading then book club would be a terrible idea, but if you enjoy reading, it is a pleasant activity, and it gets you out of the house, and it has you interacting with people you wouldn't interact with normally and I think, especially as a med student where your time is so busy and often spoken for, then having a kind of structured way to get in your hobbies was very helpful.

Interviewer: So, you think it improved your wellness? Is that what you're saying?

Participant: For sure.

Interviewer: Okay. Is there an element of self-care?

Participant: Yes, in that- I mean I think wellness in general is all about self-care and I think without kind of being forced to put it on your calendar and think about it and schedule it, you might let it go to the wayside.

Interviewer: Does the book club make you read more?

Participant: Yes. It does make you read more. I can attest to that because my reading has gone dramatically after becoming an intern despite the fact that I work less hours— well I guess not as a second year. But, I work less hours in residency than as a third-year medical student and I read more as a third-year medical student.

Interviewer: Wow. Why is reading more important?

Participant: For one, I think, like I said it just forces you to make plans for other things you enjoy doing that are not work, so being kind of coerced almost into getting your reading done- it was good for that. Also, the books that you read in book club— I read on my own, I'm just reading books I'm personally drawn to, but book club forces you to read books you might not read otherwise and expands your horizon and kind of opens your eyes.

Interviewer: Are the books that book club recommends something that you wouldn't have chosen for yourself?

Participant: So, obviously I was, for a couple of years, I was picking the books, so that's an unfair question. But, certainly at the beginning, like "Animal Vegetable Miracle," I never would have read a book about some lady farming in the mountains of somewhere in the Appalachian. And so, yes, it definitely made me read books that I never would have read, and I enjoyed them. So, that's where I think the value of book club comes from.

Interviewer: So, have you ever been exposed to literature as part of your formal medical training?

Participant: So, not in medical school but like in school, leading up to medical school I took a Medical Anthropology class and we read the book “When the Spirit Catches You and You Fall Down” or whatever it’s called. I did not take the Humanities or whatever it was that you could get credit for at med school, I didn’t do that one. But, I think the difference is that when you have to read as part of your formal training, people are being forced to read and some of them don’t want to be reading and it’s generally painful for everyone involved because half of the people don’t read it, half the people have no idea what’s going on, and it’s just like this painful forced discussion.

Interviewer: Right, right. Yeah, there are readers and then there are non-readers. People who love it, and people who hate it. So, I guess you didn’t really have a formal curriculum in medical training because— like some schools they have a formal curriculum where they— like Colombia or George Washington— no yeah, GW— so they have a curriculum where the medical student can choose a literature path, and they have a curriculum, and they meet once a month and things like that.

Participant: Yeah, I think there was an elective that you could take at like lunch time, and if you did it you got like a week of vacation as a fourth-year med student or something like that.

Interviewer: Oh really, I wasn’t aware of it.

Participant: With NAME. Or— I think it was with NAME. But, either way I didn’t do it.

Interviewer: I was wondering if NAME was focusing more on the medical literature rather than the fiction in general.

Participant: Yeah, I have no idea.

Interviewer: Oh, okay. Alright. I didn’t know that. I’ll put that in— okay. Well, that’s it.

Participant: Well good, hopefully I was helpful.

Interviewer: You were very helpful. And, you were so eloquent. I was like you’re already talking like a lawyer.

Participant: I went to school for this, I paid a lot of money for this.

Interviewer: Yeah, alright. Well, so nice to talk to you. **[crosstalk 00:11:15]** Excuse me?

Participant: I said, I hope everything is well in Gainesville. But, yeah, I will certainly get you NAME’s and NAME’s contact information.

Interviewer: Great, thank you so much. Thank you so much.

Participant: Yeah, no problem.

Interviewer: Take care. Bye-bye.

Participant: Thanks, have a good weekend, bye.

STUDENT 4

Interviewer: —start asking you questions, okay?

Participant: Okay, yeah sure.

Interviewer: Okay. So, how did you get involved with the book club? Do you remember your first meeting? What was it like?

Participant: So, I got involved with the book club like literally, it was August before my first year of medical school started. I remember it was like the week before medical school started maybe and I got an e-mail about the book club and it just so happened that you guys were reading the latest book by my favorite author and I was so excited that I clicked reply to like say yes that I wanted to be a part of the book club, and I accidentally clicked 'all' so everyone in the medical school got my e-mail about how much I wanted to be in the book club. It was a little embarrassing. And then I came to the first meeting, it was maybe September of my first year of medical school and it was lucky because I had already read the book that you guys were reading before medical school had even started and I'd even like, because this was one of my favorite authors, I'd even gone and gotten it signed by him and seen him speak about it when he came to my city like over the summer before medical school, so it happened to be a book that I really really cared about. So, that was why I was able to come, but then I didn't come back for like, I don't know, two years because I didn't have time to read books anymore, I started reading the books for the book club several times through those two years, but I never finished them, so I didn't come back until I was on my PhD.

Interviewer: And what was the first— do you remember what was the first meeting like?

Participant: Oh yeah, I totally remember. So, it was at— I think it was at Dr. NAME's house, and like I said I was a first-year medical student, so I didn't know anybody, like at all. I didn't know people in my class, I didn't know attendings. And I went to her house and she has a really nice house, and there were a couple of upper year students there. I can't remember who they were, I don't know that I ever met them again because like I said I never came back. And she was there. I don't remember who else was there among the attendings. I knew she was there because she interviewed me for med school, so I knew who she was. And then, it was funny because everybody had like hated this book. Nobody understood it, everyone was like what's going on in this book, it's such a weird book, and I loved the book. I thought it was brilliant, and like I said I had just seen the author speak so, I was like kind of— I wouldn't say I was fighting with people about the book, but I was defending it because I really loved the book and I also hadn't been in medical school for very long, and so I guess I hadn't internalized that it was a very hierarchical culture, where when attendings and people are saying things you don't like, you don't necessarily argue with them as a medical student, especially a first-year medical student. And so, if it hadn't been my very first— if it hadn't been my first day of medical school I might

not have done that. And in retrospect, I was really embarrassed when I kind of realized how it is in medical school, that it's unusual for a first-year medical student to be arguing with attendings on a book. I was really embarrassed about it, but at the time, I don't know, I thought it was fine, so I did. We had this long long discussion about this book, which was, what was it—*The Buried Giant.*

Interviewer: It turned out the best discussion ever and you know, we'll touch that point in terms of the relationship between attendings and students later, and that the book club is trying to create the atmosphere of no hierarchies you know. What's a book you remember having an impact on you?

Participant: Oh gosh, what was the name of that book that we read recently about the woman who was obese?

Interviewer: Oh, *Hunger*?

Participant: *Hunger*, yeah. *Hunger* was the first book that I read after I came back to the book club, and at first, I was like—first of all I really didn't like it. I didn't like the way it was read. It was too journal-y for me, you know what I mean. I didn't think it was very well written. But, I have thought about that book so many times since we have read it, and I never would have read it or made it through it if we hadn't read it for the book club because like I said I didn't really like it, but between the book itself and the discussion that we had afterwards, I think about it all the time. Like, I think about it all the time, because I had never— I don't think I realized how much I had internalized this kind of, I don't know, maybe like scorned is too big of a word—like too strong, but certainly I think I was falling into the trap of blaming people who are obese for their obesity, and I think it's something that medicine does a lot and I had thought about that but I hadn't really self-reflected, but since then it has totally changed the way I think about obesity, it has totally changed the way I approach obese patients.

Interviewer: So, you already answered the question of what impact, right? That's what you were trying to elaborate?

Participant: Yeah, I think with that book I realized—the first thing that I realized that I was really embarrassed I didn't realize was like, nobody wants to be obese, you know what I mean. And when you tell an obese patient "you're obese" they're like yeah, of course I know that. Like, I live with this every day; it impacts every single thing that I do, and I had never really thought about how hard it is to change that and how much is probably involved in becoming obese, and it's not just a matter of laziness or letting it go, that there's often like trauma and you know, the person has tried to lose weight over a really long period of time without success. So, when you approach them with "have you thought about losing weight," that's actually really terrible and condescending and not at all going to have the impact on your patients that you want it to have. Yeah, so I—

Interviewer: So, how has that impacted your personal life—your professional life, sorry?

Participant: My professional life? So, I really took that to heart. I think when I approach patients for whom their weight is the major thing that is impacting their health, I haven't really figured out exactly the best way to approach it. I mean one of the things that really stuck out to me from reading that book is the amount of shame that is involved with obesity, and how doctors can really, kind of, make their patients feel alienating by focusing too much on their weight. So, I think I've tried to approach it in a way that is more sensitive and also, that doesn't just like assume that the person needs weight loss counseling, and that'll help them. But, that it's actually a really hard and long-term thing to lose weight when you're obese and that sometimes what the person needs is just compassion from the doctor, rather than just being focused on the person's weight, you know.

Interviewer: Yeah. And what about your personal life?

Participant: I think, I think that I wouldn't have said that I was biased against people who were obese in my personal life, like you know, I have friends who are overweight but I think when you're—especially when you're in medical school, you tend to be around really fit people who are really hyperaware of their weight, and when somebody isn't that way, I think there's a tendency to judge them. And I feel like I've become less judgmental. I think I've kind of opened my eyes a little bit to how many things might be going on in that personal life, you know, that they don't necessarily have control over.

Interviewer: Yeah. And what are your interactions with—what are your interactions like with members of the book club, such as interactions with your fellow students?

Participant: The students that I know who are in the book club are mostly acquaintances of mine, like they tend to be people I know for other reasons as well. I'm not sure I'd call them 'friends' exactly because we don't, you know, like hang out. But, I tend to overlap with them in more than one way, you know. A couple of students I know because we go on the same service trip. A couple of students like—one student I know because we're in the same program. So, I think with the students, I don't know that I interact with them so much outside of the book club. I don't know how—I mean I guess we talk about the book club sometimes, but mostly just to remind each other when the meetings are. With the faculty members, I would say I probably would not have ever interacted with the faculty in the book club, you know as a student. And so, in that way I feel like I have relationships with people that I wouldn't have otherwise, that are closer than I would have otherwise, you know, that are more personal. You know, the only person—I mean, like I said, I knew Dr. NAME because she interviewed me, but I had never—I hadn't seen her since. And Dr. NAME, she taught some of the first-year curriculum to me, but also I hadn't ever seen her since, or outside of classroom, you know.

Interviewer: So, do you think this is one of the benefits of book club that you get to know the faculty members better?

Participant: Yeah, I think that that's part of the benefit of the book club. I think that's part of it. I think maybe the personal relationships are more important with the faculty than they are than with the students, both because the faculty come more frequently, you know like they're more consistent, and because the students, I kind of already interact with in other ways. But, the

faculty, I would never have met, like I don't think that you and I would have ever met outside of the book club.

Interviewer: Sure, yeah. What is the benefit of the book club for you? Why do you attend?

Participant: Well, I attend both for that personal relationship with the faculty, and also because I don't always make time for reading in my life. In medical school, I really had trouble making time for reading in during the school year, I just didn't do it. And it's something that's been important to me my whole life. Like, reading fiction is how I self-care you know what I mean, and during medical school I completely did not have time to do that at all, even when I had vacations and stuff I didn't have time for that. And so, with the book club, it's just like I get an e-mail that reminds me I'm supposed to read something, and then I download it on my Kindle or I buy it, and then I at least know I have to—it's how I justify sometimes, making the time to read something.

Interviewer: Some pressure, right? Some pressure.

Participant: And like a reminder that I have to get it done, and not like I have to. If I don't finish the book I don't feel pressure, but it's just like a little bit more justification, you know. Like I couldn't justify it just for myself, but because I have the book club, I can justify it, you know what I mean.

Interviewer: Yeah, yeah. And then so, you touched on—so is there an element of self-care?

Participant: Yeah, I think that there is. I think there is. I definitely think there is. Like, especially, I can think like this last month, sometimes I don't enjoy the books that much, like I don't love the books. Like that book about obesity, as I was reading it, I was like "this book is annoying." But, then when I actually got to the discussion, I really liked the discussion. So, it helps me push through books that I maybe wouldn't normally read. Another example, is that book that we read about diabetes. I'm not sure that I would've read that whole book if it was just me because I'm kind of would have been like "I don't know really care about this woman and her childhood diabetes."

Interviewer: You're talking about *Breakthrough: Elizabeth Hughes, the Discovery of Insulin*.

Participant: Yeah, that's it.

Interviewer: Okay.

Participant: I started it and I was like "I don't really want to read this book," but I pushed through it and actually really enjoyed the book and I also really enjoyed the discussion. So, a lot of times I will—it will be a book that I normally wouldn't want to read. But, yeah.

Interviewer: Does the book club make you read more?

Participant: Yeah, it definitely does.

Interviewer: Why is that important?

Participant: I think it's important because—I think it's important for a bunch of reasons. It's important for my mental health to make sure that I'm reading, and I don't always make time for that in my own life. I tend to not think that's important and I can just tough it out, and that's not super good to do to yourself long-term, so it's important for that reason. I also find that the ideas in the books that we read often, I'm thinking about them a lot after the book is done, and they change the way I see things. So, I think it's important for that reason.

Interviewer: Are the books at book club reads you would have chosen for yourself? I think you've answered that question, right?

Participant: I mean sometimes they are, like I said *The Buried Giant* was like- I love Kazuo Ishiguro and the book that we're reading this month, although I won't make it to the book club this month I just realized.

Interviewer: Oh, you won't, shoot.

Participant: No. Yeah, that Sunday I'm coming back from a conference, so I won't be back in town, but I am actually really enjoying that book and it was a book that I wanted to read anyway so, sometimes they are. But, I would say about half the time they're not things I would pick for myself, and they're not things that I would keep reading, you know. Like, I start them and I'm like "oh, I don't want to read this," but then I keep reading and I'm like "oh, actually this is okay," you know, actually I kind of like this.

Interviewer: Have you ever been exposed to literature as part of your formal medical training?

Participant: I don't think so. I mean, I think maybe we were all supposed to read a book when we got to medical school, about med students experiences or something like that. I kind of remember that but I don't think that we ever talked about it as a class, like I don't think we ever actually used it. And then I started to do the Narrative Medicine, whatever that is- track. But, I just ended up not having time for it. It was too much, too much like busy work, so I didn't do it.

Interviewer: So, how was that experience similar or different than the book club- the Narrative medicine?

Participant: The problem with Narrative Medicine was that everybody was doing it to fulfill a requirement, and most people didn't actually take it seriously. So, you would go to the meetings and I felt like I was the only one talking all the time.

Interviewer: [crosstalk 00:15:30] But, what about the book? Were they interesting?

Participant: What?

Interviewer: Do you feel like the books that they pick were interesting to you?

Participant: At Narrative Medicine?

Interviewer: Mhm.

Participant: A lot of the readings were short, and I didn't really like them. I think the way it is structured was you had to do a bunch of smaller readings and go to these meetings about them, and then you could pick a book to read. And I read this book called *Mendel's Dwarf* or something like that, which I didn't like very much, and then we never discussed it or anything like that, so—

Interviewer: Oh, so that—part of it is assigned reading, part of it you pick your own? Is that what you're saying?

Participant: It was like you can pick a book from a list, like there were a list of options. So, I picked that one. I don't know why, maybe I just missed the meeting, or maybe I was the only one who read that book, but we never talked about it and I kind of felt like it was a waste of time, because I didn't like the book and there was no discussion.

Interviewer: Sure, okay. Well, sounds good. Thank you very much. I'm going to turn off and then I have some other questions for you, okay?

Participant: Yeah.

STUDENT 5
Interviewer: Okay, I'm going to start recording okay?

Participant: Okay.

Interviewer: Okay. So, first question is, how did you get involved with the book club? Do you remember your first meeting? What was it like?

Participant: So, I first got involved with the book club at the end of my second year of med school, beginning of my third year. I remember having seen e-mails in the past, but never knowing anyone personally who had gone so I was kind of hesitant to go. Then, a couple of friends in my class asked and NAME invited me, and said they were going to go to this meeting, and so I decided to read the book and join. The book I remember the book was *Buried Giant* by Kazuo Ishiguro and there was—it was just very interesting to be at a professor's and a teacher's-an attending's house, and not be doing anything related to medicine. It was actually very freeing in a lot of ways, and kind of exciting to see kind of behind the curtain of people in academia and within the context of medicine, and to share an enjoyment and an interest in discussing the book together, with people of all different levels. So, with my classmates and then also with people who were usually teaching me and grading me. So, I think it was fascinating and really beneficial to me from that point on to kind of form friendships with the people at the top of the spectrum of trainings, and to talk about things not related to medicine. It was kind of interesting.

Interviewer: So, what books—what's a book that you remember having an impact on you? And if there is, what impact?

Participant: I think, in a lot of ways, that first book that we discussed together, *Buried Giant*, had a large of impact on me, mainly because I didn't really enjoy that book all that much when I was reading it on my own, but when we sat there and discussed it I realized how nuanced and complex it was, and I found myself almost wanting to go back and start reading it again because when as I heard what other people got out of it and we started to kind of analyzing and thinking about it I realized that there was a lot of depth to the book and a lot of really interesting concepts to the book that I hadn't seen before. I think it had an impact on me both in that it showed me personally the value of sitting and kind of talking through things, in and outside of medicine, and that there are other perspectives that can be had and sometimes you can learn a lot from other people and the way they perceive something. I suppose in some ways that may carry over into my professional life as well, in terms of trying to hear patient's sides of their stories, and not just kind of go into an encounter or interaction with people based on—assuming that I know the answer, assuming that my perspective is always the right one. Another book that I think professionally was really interesting for me was *Imagine Me Gone* which is a book that deals with depression and anxiety and kind of its impact on family members and how they try to cope with the main character since they are struggling with this disease and I think, for me, it was really helpful to hear this very honest portrayal of what it's like to be a family member struggling with this disease, with anxiety and depression, because that's not something that I personally or really any of my close family or friends have had to struggle with. So, I think kind of stepping into the shoes of someone who is walking alongside a person or a patient who is struggling with this is helpful for me, as I care for my own patients who have those problems and also care for people whose family members are struggling with depression or anxiety.

Interviewer: Okay, so what are your interactions like with members of the book club? What about your interactions with other students?

Participant: So, for other students in the book club, a couple of the ones who were there regularly became some of my close friends over the last several years of med school, as we **[unintelligible 00:04:12]** and we dispersed, I found that I wasn't always necessarily around the same people I had been before, but this is kind of a meet-and-go happened every couple of months or so, and even when we were on rotations there was a core group that always tried to make it. I just found that as I got to know these people outside of the world of medicine, I realized that we had this shared love of reading and trying to read kind of deeply and broadly. Yeah had some really good friendships with those people and it helped me meet all of them.

Interviewer: What about your interactions with physicians?

Participant: So, my interactions with the physicians in the book club was one of the highlights about it actually. I think I ended up going into Internal Medicine, and so many of the internal medicine faculty were core members of this group and spoke a lot about the field and the fact that it's for people who are interested in broad things, which I found to be true. They can be your **[unintelligible 00:05:14]**. I feel like I got a lot of mentorship out of the position. Even though discussions were times we, for the most part, would steer away from medicine, there would be

times as I was moving through clerkships, and then moving to applying to residency, I kind of valued the opinions of these physicians a lot because I spent a lot of time with them, and I feel like I was able to get a lot of really good guidance and advice. It also felt like I had a connection with them that was more than just the classroom, which again was very enjoyable and a fun experience. Whenever I saw them in the hallway, we would sometimes talk about medicine or we would talk about what we were reading for the book for club or for otherwise, and I really liked that.

Interviewer: Great. What is the benefit of the book club for you? I think it's a little redundant. And why do you attend?

Participant: Yeah, so I think medicine is a field where it's really needed for you to jump all in and really lose your sense of balance, if I might. I think there's a lot of wonderful in the field, a lot of good from it, but that's a very real dark side. Even as a student I saw that- as a medical student it was necessary to shed the parts of life that aren't important for the sake of just trying to be all in. And while I think it's important to be excellent at your work and to strive to be a really phenomenal physician, I also think it's really important to be a balanced and real human being. And one of the things that is really important to me, is both reading for the sake of enjoyment, in terms of jumping into stories and the worlds that other people create. I think that that can be a really nice contrast to the world we live in where things aren't always **[unintelligible 00:07:10]** as can be in fiction. I think both fiction and non-fiction books provide you opportunities to experience and see world views that you might not have been able to see that closely, to see how other people interact with the world and experience the world and to learn off of that. I think as a human that's important, for anyone, but I think for a physician that's particularly important because we're called to kind of step into the lives of our patients and try to understand the things that are important and understand their values and their goals, and kind of practice experiences- looking through people's eyes that you get from reading literature, it prepares me to do that well.

Interviewer: Yeah. I think you sort of touched on the next question. Is there an element of self-care?

Participant: Yeah. I would definitely say there is an element of self-care in that. I think it's vitally important to have things that interest you and liven you and ones that also can give you a little break from medicine. I think, for me, reading is one of those things and so being able to set aside to read and then sit down with a group and discuss the book over snacks and over a kind of light environment, was definitely rejuvenating.

Interviewer: Does the book club make you read more?

Participant: I think the book club did make me read more and does make me read more. I think to this day, if I look at kind of where my reading patterns where at the end of college and beginning of med school, and I look at how they have expanded over the course of the years I was involved in the book club and I continue—I think I'm reading a lot more than I was four, five years ago. I think I'm reading a lot more proudly. If you can't tell I have a little bit of a bias against modern contemporary fiction, things written in the last few years, tend to be focused more towards more classical writing, and so those are the things that I know, and I think that's

changed, I look at the lists of books that came out in the last year or two, I look to, even though I'm not in book club anymore, I look to the things that people in it are reading, and I think that contemporary just made a push to my boundaries in the things that I'm reading and the amount of time I spend reading.

Interviewer: So, why is reading more important? Why is that important?

Participant: Trying reading more is important for the reasons I already mentioned, but for the sake of getting more perspective, but honestly at this point a huge extent of it is just to have balance within a crazy residency schedule to—with the limited time that I have off when I'm not in the hospital, to have things that I am really excited about spending time on and reading is something to me that is—it is a break and it is relaxing but at the same time is—I think that it's helpful. So, having those hobbies or things that meet those goals of being a break but also being good in and of themselves is when your free time shrinks, as it tends to do at the beginning of residency, being able to kind of make your free time have extra bang for your back of with being both **[unintelligible 00:10:38]** and beneficial, is really important.

Interviewer: Are the other books that the book club read—are some of the books that the book club chosen you never would have chosen them for yourself?

Participant: Oh, absolutely. There are books we read for book club that I never would have chosen myself. I think as I discussed I had, for whatever reason I'm not really sure, a bias against like truly contempary fiction. We read a lot of things that were national book award winners or were nominated—written in the last twelve or eighteen months, we were reading them, and I really enjoyed them. And I learned there are a lot of intelligent, and bright writers today and it made me kind of excited to look for their next book/ Some of the books we read, I ended up enjoying, and pursuing others written by the same author. I think some of those are things that I would read for myself, so it was a nice mix. Some things I never would read, but I'm glad I did read. Some things I never would have read before and now I enjoy. And some things that I probably would have read on my own.

Interviewer: Great, so last question. Have you ever been exposed to literature as part of your formal medical training? What was that like? How is that experience similar or different than the book club?

Participant: So, I think that the only literature I can think of that I was exposed to as part of my medical training was the movie **[unintelligible 00:12:15]** which we looked at in the context of our first year medical school we were learning about the general orientation to cancer and thinking about breaking bad news and thinking about the **[unintelligible 00:12:28]** of oncology-I thought that was particularly literature. It was to look at this kind of fictionalized aspect of medicine—

Interviewer: Those were movies you said? You said those were movies, not books?

Participant: It was a movie that was based on a play, and then we were encouraged to read the play and so they kind of offered the **[unintelligible 00:12:54]** and studying that was interesting, I

think it was very useful and I think there are a lot of aspects of literature that would be really helpful in formal medical training. There are several books I think would be really benefical for medical students to read and to think about over a period of weeks or months, over the first year or two of medical school. But, I don't think as a whole that that happens very often.

Interviewer: Did you address the question how is that experience similar or different than the book club?

Participant: I think it's similar in a lot of ways. I think it—as we sat down either watching or having read it, and we talked about kind of how we—how the experiences that happened within the context of the fictional world related to the ones that we, at that point as first year students, really were expecting to see **[unintelligible 00:13:55]** and we kind of tried to prcoess through how characters reacted, and how we saw ourselves within some characters and this kind of experience of there's no worries- I think that's very similar to the experience we had in the book club of we sat there and kind of talked about, in some of the cases, how we could draw parallels between the characters we were reading about and the themes we were reading about in our own practice. I think it was also similar in that while it was medical and **[unintelligible 00:14:27]** it's not the same kind of knowledge you normally talk about and kind of testable medical knowledge. It was much more the psycho-social experience, which I think is the sort of thing that gets captured really well within literature. So, I think in a lot of ways the lecture was really similar, the difference is it happened once and we didn't really have this as an ongoing experience and so it doesn't have the continuity that we have with the book club.

Interviewer: Did you do Narrative Medicine?

Participant: I did not, no.

Interviewer: Okay. Some students have contrasted this with Narrative Medicine, so I was just curious. Okay. Alright. Okay, thank you, I'm going to turn it off.

Participant: Okay.

STUDENT 6

Interviewer: So, how did you get involved in the book club? Do you remember your first meeting? What was it like?

Participant: Yeah, so I was in med school at the time, and we have e-mail listservs, and someone, one of the med students, someone in my class I think, I forget her name, I think she may have been an MD/JD student; she had sent the e-mail out about the book club. I can't remember if she had started it or she was just continuing it, and it seemed really interesting. I always enjoyed reading and I thought it would be fun to have a book discussion with others.

Interviewer: Which year of medical school were you? Were you in undergrad you said?

Participant: No, I was in medical school. I was a—I think I was a third year at the time. Third or fourth year. Yeah, third year because the first year you're already reading too much. **[unintelligible 00:01:52]**.

Interviewer: So, do you remember your first meeting?

Participant: Hold on one second.

Interviewer: Sure, I'll pause.

Participant: Sorry, go ahead.

Interviewer: Do you remember your first meeting?

Participant: You know, I remember going to several meetings, I'm not sure which one was the first one. I think the first one might have been in one of the other med student's apartment. I don't know if it was just students at the time or if there were faculty as well, but I remember that. Then, I remember, of course, once we met at Dr. NAME's house- that was fun.

Interviewer: Yeah. And then what was the book that you remember having an impact on you?

Participant: Oh gosh. So, I think they were all quite interesting and I enjoyed discussing them all.

Interviewer: Is there anything that impacted you professionally?

Participant: I would say all of them, in some ways. I think it wasn't just the books, but the books combined with having other faculty and mentors there to discuss it and share their views and things like that. You know, because it's one thing to read a book, like let's say you read **[unintelligible 00:02:11]**. You read about this young man's immigrant experience, his life, him values, and things like that, and you may form your own opinion's and—if I have patient's that come from the same demographic or cultural background, how might I interpret those actions and behaviors as opposed to someone who grew up in my background, who's very different? And then having other faculty members there **[unintelligible 00:02:38]** share their own opinions, who may have their own views and agree or disagree, but then validate your views and encourage you to interpret it that way to be more broad minded about, you know—those economic, demographic and social impacts and disparities, and how that influences health care. It's just really rewarding because a lot of the time as a student all you're hearing is critical information, you know, science and things like that, and any of the aspects of medicine are so important but sometimes, not because people are lazy, but time constraints, sometimes because of exams, it kind of doesn't get pushed or promoted as much. So, to have an environment where you have faculty speaking about these things and encouraging thoughtful analysis, and kind of showing how medicine is really rewarding.

Interviewer: I see. So, that contrast from the classes you're taking. So, there's more humanity, is what you're saying, with this. The classroom is more science oriented—is that what you're saying?

Participant: Yeah, I think so. Even your privilege. I had the opportunity to work with you, Dr. NAME, both as a third-year student and also getting to interact with you through book club. And it was wonderful to work with during third-year, I thought how you approached patient care was really good.

Interviewer: But, I think that's more the third question. I think that's more the third question. I think the third question is more like how did you interact with other people, but the second question that we're still on is about, how those books affected your professional life. So, that kind of is a different angle.

Participant: So, I would say those books, and many books I read, the most important—it offers me different perspectives to understand people from different backgrounds. You know, people who are older, people who are younger, maybe people who are from different countries, different cultures, speak a different language—unless you read a book and you understand views from people's perspectives, you may not consider their viewpoint, and you may not understand how it affects their decision-making. So, it's really huge because in my professional life I'm a **[unintelligible 00:05:04]**. I see so many patients from so many backgrounds, and I don't always understand the context of why they might be choosing to do something or not do something. But, reading about things offers me a little more wealth, it allows me to draw form an experience pool that I don't have, that others have. And I can kind of glean from that wisdom, from those perspectives, or I can understand sometimes the value of not **[unintelligible 00:05:28]** because I've read enough to say, okay, I can imagine a character from a book in this situation, why would he make this decision, why would you do this choice—because on the surface it doesn't make sense, but when I consider it from that perspective that I learned about **[unintelligible 00:05:43]**, even more so than real world people sometimes, it's definitely—the context changes dramatically, absolutely.

Interviewer: Yeah, that's interesting. So, it kind of allows you to meet more people that you never would have otherwise met in your real life, and then see their perspectives, okay. Yeah, actually even in real life, even if you meet people, you may still not read into their inner world as deeply as when you read in a book. That's a good point, and I never thought about that. What about personal life? Has—are there any books, I mean books in general, that affected your personal life?

Participant: In some ways, it's always hard because, on then one hand I would say yes it has, but on the other hand has a book really influenced me, or did I already have the view and the book confirmed it. It's like in medicine, why do we do RCPs? Because it's sometimes hard to sort out positive inference from different interventions. But, I do really believe it has impacted my personal life. For me, reading books probably has made me more of a patient person, you know. Because if I'm reading these books—take *Life of Pi*, for example. The whole book is an allegory, but a lot of the decisions that are made that are made harmful in error, sometimes are done out of haste, or they're done because people, you know you're hot and you're just thinking

about my needs now, not my needs in five or ten years- not always the moral pros and cons of each action and it kind of serves as allegory, but then you incorporate it in your life and think okay, what are the lessons, what are the morals here? How do I gain understanding, so I don't make similar mistakes in my personal life? To some degree I think that's also our upbringing. When I was a kid and read all these morals and stories and tried to incorporate that to your life and your decision making.

Interviewer: When you look at this list, which are the ones that you've read so far? I know we're digressing a little.

Participant: I think the ones I've read—

Interviewer: With the book club.

Participant: So, basically, most of the ones before 2013. I think between 2012 and 2013, I think that's when I finished my residency and I wasn't attending quite as frequently.

Interviewer: So, you read *Animal Vegetable Miracle*?

Participant: No, I think I missed that one.

Interviewer: Oh, okay. *State of Wonder*?

Participant: Yeah, *State of Wonder* I did read.

Interviewer: Yeah, did you like it?

Participant: I did.

Interviewer: That's nice. Okay, so now we're going to the third question, which I think you already started discussing, so if you don't mind—

Participant: I'm sorry.

Interviewer: No, that's fine. What are your interactions like with members of the book club? First interaction with maybe a general statement, and then we can divide it into the students and the physicians. So, how was your—

Participant: It was great. It was the best part of book club. I mean, reading the books was fun, but getting to attend, hear different people thoughts being shared, and to be doing it with other people who are in the medical community, so you know a little bit of that shared perspective. And you get the perspective of folks who have been doing things for a long time, and they'll validate good views, or they'll kind of offer nudging corrections of views that kind of conflict with **[unintelligible 00:09:28]** fantastic.

Interviewer: And then what about with the other students?

Participant: That was good too. I think it was really nice because as a student you're also able to commiserate a little bit. It gives you an escape and shared interest beyond just studying and exams. That was really nice; I think there's a lot of talk in medicine now about burn out and stuff, and I think anything you have, or you get to do things with other students, in a friendly environment, that kind of takes away from stressors and it was great. For me, I looked forward to them. They were definitely something that helped me avoid burnout.

Interviewer: Interactions with the physicians? I think you said all these, but can you brief—can you actually say them again, because you said them very well.

Participant: It's really—you know, your interactions with physicians is a lot of time driven by your context of how you interact with them. When I'm working with you, Dr. NAME, as a third-year student, it's around patient care, medication, things like that. There's a lot of aspects of compassion, and so forth, that comes through, but it's all focused around medicine. Now, when I'm interacting with you in book club and we're discussing a book, I get to have discussions and see your perspective on things in a different environment and it's because we're in a different context and it's really neat to do that. You know it's neat to see that aspect, and be validated, say "okay, I saw Dr. NAME working in a clinical environment, and I really appreciate how she does stuff, but now I have other aspects of here I get to see, and I can see similarities I have and I can share. So, sometimes if I'm seeing things and I'm like, "I don't know if I'm like all these clinicians- they seem super professional and always focused on only medicine," now, I've realized that there's more to them and it's okay for me to have other interests.

Interviewer: What is the benefit of the book club to you? Some of these questions may be a little—you touched on already. What is the benefit of the book club to you and why do you attend?

Participant: I think the benefits for me was- it was enjoyable, but it also kind of motivated me a little bit. Sometimes you get so caught up in medical stuff it was like, "okay, I have a deadline; by this point I have to have read this book otherwise I won't get to discuss it." It kind of pushed you a little bit. Sometimes, I think we all have more books on our bookshelf we want to read than we are actually reading. So, that was good.

Interviewer: Yeah. Is there an element of self-care?

Participant: Yeah, I think so. I mean there's always a selection bias. We were always—we aimed for self-care, for things that would help us. But, yeah. I think, you know, as a student especially, you need opportunities to do interests and to just have breathing space from just the overload of medicine. So, that's—

Interviewer: Yeah, you also mentioned earlier about the reducing burnout, that would fit here too kind of **[crosstalk 00:12:40]**.

Participant: And it's not just about doing those things but sharing those experiences and interests with others. It like reinforces the good things, like positive reinforcement for self-care.

Interviewer: Does the book club make you read more? Why is that important?

Participant: You know, if I'm honest, I've always read a lot. You know, even before I came to medical school. I think what the book club did do was it kind of helped shake me out of my stupor of reading only medical stuff a little bit, and transitioning me back to the reading patterns I had before I came to med school.

Interviewer: Why is it important?

Participant: Why is it important to be reading? Oh, I mean, gosh, it's like asking why it's important to grow in life. I think, if we don't want to be static and we want to challenge ourselves, we have to push ourselves to go beyond our boundaries to learn things, you know. I mean I guess you could just watch Netflix all the time now and do it, but I think reading still offers the best way to do that.

Interviewer: Are the books that the book club read—some of the books that you never would have chosen for yourself?

Participant: Oh, absolutely. It was actually kind of funny because I remember when I was at the book club sometimes I'd be the only guy there, and there was a question of whether we should have more male-friendly books to attract more male members. Sometimes, **[00:14:17]** was like that all the books were maybe gender-imbalanced. But, no I think—looking at the list I can't believe I missed that—when you guys read *Ender's Game*, that would have been fantastic. That's like pure science fiction, you know.

Interviewer: Yeah. But, you know that actually, in the current book club, there are no—everyone dislikes science fiction, I'm the only one who likes it. Yeah, we can talk more—yeah. We'll read everything except for science fiction. Okay, so there are some that you would not have chosen.

Participant: Oh, absolutely. I would have never read **[unintelligible 00:14:59]** and I loved it, you know.

Interviewer: I see. Have you ever been exposed to literature as part of your formal medical training, like through your medical school? Have you had any formal exposure?

Participant: Yeah, we had Narrative Medicine classes- NAME did that. I hope she's still doing them, that was really wonderful. So, we had a little bit of that. It was an elective, but it was still there.

Interviewer: How do you contrast this with the book club experience?

Participant: It was often- it was a little bit different. It's a little bit more structured and also—

Interviewer: The Narrative medicine is more structured?

Participant: Yeah, it's just like because you know, you have learning objectives and things you're trying to get across and stuff, but you're not **[unintelligible 00:15:48]** to an academic environment, right. So, NAME is great, I think she would love to just have people at her house to discuss stuff, but you know you have to have structure and you're showing that your students are getting something meaningful out of it. So, book club is a little bit more informal and in some ways that makes it nice as well because, you know, it changes the barriers and it changes a little bit of the role of discussion and things like that.

Interviewer: Yeah, so that would—the Narrative Medicine is the one thing you can think of. Well, thank you. I'm going to turn it off.

STUDENT 7

Interviewer: Okay, okay. We'll start all over again. So, you were a medical student in the book club. What are you doing right now?

Participant: Now I'm an OBGYN resident in Boston. It's my second year of residency.

Interviewer: Excellent. So, how did you get involved with the book club? Do you remember your first meeting? What was it like?

Participant: Yeah, one of my good friends mentioned that she was in a book club, and as someone who has always been a reader and who read recreationally on my own, I thought that it would be fun to join. I didn't really know what to expect because I had never been in a book club before, though I had taken, literature classes and things like that in college. So, at my first meeting, I remember showing up and I remember I was a little nervous because I didn't know how well read people would be and what the discussion would really center around and, you know, the kind of normal set of expectations and worries that you have before you have a new experience and I really liked it because it was—everyone sort of interacted in a normal, friendly way; I felt like it really broke down barriers between myself and attendings that I normally worked with. We acted as more peers and less like, you know, an attending and a medical student. And I also really felt like it was a good way for people to articulate their responses to books and it made me feel like I got to know a more extensive and deeper side to a lot of people that I worked with in medical school.

Interviewer: So, what's a book that you remember that had an impact on you?

Participant: It's funny that you brought up *Fates and Furies* not being a very popular one that we read because that's actually one of my favorite books.

Interviewer: You mean *Fates and Furies*? *Fates and Furies*?

Participant: *Fates and Furies*, yes. And you know, I think the reason why it really stuck around was because it had these two dueling perspectives, of like the wife and husband, and this same story was told through both of their eyes and I feel like, not only was that something that applies

personally so frequently, but also something that we see as physicians all the time. You know, you constantly hear the perspectives from the patient, how you see the issue or illness, how the family member might be perceiving what's going on. So, it just kind of is a reminder—really set the tone of the last experiences I had moving forward with my training.

Interviewer: So, you are referring to how it impacts on your professional life?

Participant: Yeah. Yeah, and I think I enjoyed it at the time because everyone has had those arguments, or someone just sees something completely different than you do in their personal lives as well. I just like that book a lot.

Interviewer: And is there anything that impacted your personal life?

Participant: I think—I mean, not, you know, explicitly. When I joined the book club it was about a year and a half after my father passed away, and so it was actually really nice to have—to sit around and talk about feelings that you had that weren't necessarily your personal feelings but just your reflections on a book and the thoughts and attitudes of other people, and I think it helped me kind of just like be a little bit more comfortable opening up to people and feeling a little bit more vulnerable, when I felt very vulnerable in medical school. So, I wouldn't say there was a specific book that did that, but book club in general.

Interviewer: Yeah, so there wasn't a book you read, that you would be doing things in your personal life and you'd be like, you know, it would remind you of certain scenes or certain plots in the book.

Participant: Not right now; not necessarily, no.

Interviewer: Okay. What are your interactions like with members of the book club? How's your interactions with students? Fellow students?

Participant: Well, I was friends—pretty good friends with two other members of the book club, so that was nice. They made me feel really comfortable, and then I made a lot of friends through the book club. So, I think, you know, it was a good way to make friends and establish people you knew around campus, and people that you knew you had this connection to because sometimes in medical school it's difficult to do that when everything is just like what you're studying for your science curriculums.

Interviewer: Yeah, how's your interactions with physicians? I think you alluded to it already. Can you—

Participant: Yeah, definitely. I felt like what happened through book club was that I started to see attendings less as these really intimidating figures, and more as kind of normal people, who did the same sort of things that I did and it made me less intimidated in general by presenting when I was on different rotations or asking questions of attendings I was working with in the hospital, so in that way I felt like it really broke down a lot of barriers. And that it made my relationship with them a lot less hierarchical.

Interviewer: So, what are the benefits of book club for you? Why do you attend? Is there an element of self-care?

Participant: Yeah, absolutely. I mean, like I mentioned earlier, I've always been someone who enjoyed reading, but it's so easy in medical school to just tell yourself oh you don't have time to make yourself a healthy dinner, to exercise, to do activities you might have enjoyed before, like reading. So, having something like the book club not only gave you like a timeline to finish something, but it also made you kind of reflect on that experience, so it felt like a very worthwhile way to spend your time. You know, it didn't feel like you should've been studying for a test instead of reading the book club book. So, I think that was really important. I also think it's hard to force yourself to take some quiet time during the day. You know, we're so busy and running around and going from one task to another, the time to just sit and read was really nice.

Interviewer: And does the book club make you read more?

Participant: I think so, yeah. I mean since I joined the book club now, I read—I always read before medical school, and then I stopped for a few years, and then started book club, got into the habit of reading, and now I read, even since starting residency, have started reading a bunch.

Interviewer: Wow. So, why is that important?

Participant: I think it's important because, what I took away from intern year, is it's so easy to try to be so selfless and so invested in caring for your patients, that you forget that you have to take care of yourself. And you know, you go through so much emotionally and physically throughout residency that having something that you do to kind of calm down or reflect or that just like takes you out of your day-to-day experience and puts you into the storyline of someone else is a really nice escape. And I think the lessons that we take from learning, or at least that I always did when I was a kid, like the way to deal with problems, interpersonal conflict, emotional health, or just like reading something that's a fun and exciting story, you know that you enjoy. I think that's important because this teaches you how to handle different situations, it also just lets you disconnect from your drama, and the daily grind of what you're going through in residency or your daily life.

Interviewer: Are the books that the book club reads, some of them you would never have chosen for yourself?

Participant: Yeah, definitely. I actually remember when we read *The Buried Giant*, that's not something I would have ever picked up off the shelf, and then that was one of my favorite meetings, not because I particularly enjoyed the book, but because of the views other people had, and they had really interesting comments to say about the book, and it was a really fruitful discussion. And that I found enjoyment in, even though I didn't love the book itself.

Interviewer: Yeah, a lot of people remember that book for some reason. Just leaves a really deep impression.

Participant: Well, it's kind of a weird one.

Interviewer: And then have you ever been exposed to literature as part of your formal medical training? What was it like?

Participant: Not really part of my formal medical training, I don't think. I mean maybe occasionally we would read—I actually don't even remember reading any formal literature in medical school.

Interviewer: Some people did Narrative Medicine- did you do that?

Participant: No, I didn't.

Interviewer: Okay, so the next question is how do you contrast with this, but I guess since you didn't that question would be irrelevant. Anything else you would like to add before we wrap it up?

Participant: No, I don't think so. It's definitely one of the things that I remember most form medical school and something I enjoyed a lot, so I'm excited—I hope that you guys can get something useful out of the interviews and can keep it going.

Interviewer: Okay, thank you so much. Hold on, let me stop this.

STUDENT 8

Interviewer: Where is the speaker? Okay, are you there? Hey, NAME, you were a medical student at UF, right? My first question is—so you recently became an attending in endocrine surgery. So, the first question is how did you get involved with the book club? Do you remember your first meeting? What was it like?

Participant: Well, I first became involved with the book club just accidentally, you know I can't remember to be honest who I spoke to or when I spoke to them, but Dr. NAME was my preceptor and you know, amongst our interactions we really loved reading, we'd like to start a book club, and I remember we started it at what I believe was my third—my second or third year of medical school we started the book club, and the—I don't remember actually the first specific meeting, that was ten years ago now, I just remember that all the attendings were so generous and I remember going to different attendings homes and recruiting all these different medical students to come and join the book club and the beauty in joining because we actually got—I remembered everyone enjoyed being in a home, or an apartment, and it was just such a wonderful time where we'd gather together and have some food and discuss the book in a very enlightening way. And I remember feeling, as a medical student, it was so wonderful to have this interesting shared with your attendings who normally you only interact with at the hospital, and it was so different to interact with people on more of a personal level and you can understand kind of how they think about your book or the world and what their perspectives were. So, I think it was a kind of unique time that we had together.

Interviewer: What's a book that you remember having an impact on you, and what impact—first from a professional standpoint, and second from a personal standpoint?

Participant: I think that might vary between the books that I read during the first years of medical school and then book club was *Olive Kitteridge* by Elizabeth Strout. That book has just stood with me through all these years and I think what really impacted me about it was the perspective of the author and her understanding of characters in this kind of small Maine town, I think that it was something that I feel like kind of relates to our patients because in Gainesville a lot of our patients are also kind of from rural areas and little communities and it kind of made me feel and understand that people can sometimes have very quiet desperation or very quiet anxiety or trouble, that you don't necessarily see when interacting with people on a more superficial basis, or even when we're just treating ourselves on our medical basis, we're more focused on that. I think a very great perspective on people's internal lives and—but kind of just in my personal I just really connected with the book and really loved it and enjoyed the character development.

Interviewer: Yeah. What are your interactions like with members of the book club? You kind of alluded to it already. What about your interactions with other students?

Participant: The other students were just fantastic. I do remember—I don't exactly remember how we got in contact with people. I think we sent e-mails over the medical student e-mail list and tried to recruit people. I just remember being so excited when students were wanting to come. We would talk about books excitedly. We would be really **[unintelligible 00:04:30]** just to go to these meetings. I mean I just remember there was a lot of enthusiasm about it and that was something that really thrilled me because I actually just love reading and it was such an important part of my life, again, especially in medical school when you're so young and trying to learn about the medical profession and how to understand patients, and I feel like books and the way that authors have this really unique connection with their characters has helped me gain so much more perspective on our patient's lives. But, I just remember it was just a wonderful time. **[unintelligible 00:05:07]** I just remember we had a great time.

Interviewer: What about your interactions with the physicians and faculty? I think you already kind of said something to this.

Participant: Yeah, I did allude to that. You know, I think that I just felt very grateful that the attendings who started this book club with us, they were so interested in doing it. I was actually a little bit surprised, I thought it would be something more like medical students really gathering to have a discussion, but I was really surprised and grateful that they really took an interest because, you know, it was something outside of the hospital that we could do together. I mean, they invited us into their home, they provided us with food, everything so that we could talk about books together. I mean, I felt like that was just a step beyond what a lot of attendings and physicians would do. So, I really loved that. And I just sort of remember kind of talking—there were some moments someone would talk about things that were very personal and more personal than you would ever hear when you were just on the ward, and I got really humanized, and I think that—I'll never forget that. There were just some things that were said that I'll never

forget. It really made me think differently often about your bosses, your attendings; it makes you realize that they're also very human too.

Interviewer: And, what is the benefit of the book club for you? Why do you attend? Is it an element of self-care?

Participant: Well, I think I just really love to read and it's something I try to do myself. I remember starting medical school and feeling like a lot of my creative juices were already dry because it's a lot of just memorization and a lot of just thinking about science and a lot of about just kind of the same thing, and it was an outlet for me to just kind of explore my humanity, at least in the first couple years of medical school. So that—it was definitely very nourishing to have this time to really read and just enjoy in a very peaceful setting.

Interviewer: And then does the book club make you read more?

Participant: Yes, it definitely made me very disciplined about reading. And again, the books we chose were just fantastic and I just really—I really appreciate that, especially now, when I'm not in a book club currently, I see that it's harder for me to find books that interest me and it's harder for me to stay engaged, and sometimes I'll pick up books and put them down again. But, with book club, we were just—you know that you have to read it because you know that you were going to **[unintelligible 00:08:11]**.

Interviewer: Why is that important? Why is the reading more important?

Participant: You know, I feel like when I'm in a state where I'm reading regularly, I feel a little bit more connected to the world. It makes me feel that I'm thinking in ways beyond myself. I know that they're only characters, but I feel like they give me a better understanding of people who are not in my immediate circle **[unintelligible 00:08:49]** but people beyond, even though they're only fictional characters, a lot of characters are still very much based in reality, so I feel like it gives me a very engaging—it opens up my mind to a lot of different things. And then, of course, there are a lot of books that are not fiction and **[unintelligible 00:09:13]** can really kind of give you a wider perspective of what is in your immediate circle.

Interviewer: So, are there books that the read that you never would have picked for yourself?

Participant: You know, I can't remember. I don't think so but looking over some of the book list that has, you know, books you've read since I have left medical school- I think that there are definitely some books that I wouldn't have read on my own. So, I'm looking over the list and I see such a mix of contemporary, more classics, non-fiction, and some things that I think are just a wonderful mix of books.

Interviewer: And then some of them you would never have read if you haven't attended book club? Or no?

Participant: I think that some of these books I would not have picked up on my own. Yeah, I think that the list is very diverse and very interesting. I would have been interesting in reading it,

but I gravitate more towards just plain fiction. I think this is a very diverse kind of book **[unintelligible 00:10:31]**.

Interviewer: Have you ever been exposed to literature as part of your formal medical training?

Participant: We did a little bit. I remember in medical school we did have a part of our classes that were like the humanities. I knew that we had some readings, some excerpts that we did. But, I don't remember it being super prominent. But, I remember doing some of that.

Interviewer: Okay. So, how is that experience different from the book club?

Participant: You know, it's actually different because—

Interviewer: Different or similar?

Participant: It was similar in that there were discussions about feelings and about thoughts. It was different in that it was very medically oriented. You knew that what you were trying to learn is based on your medical—from a physician-patient relationship, and how to understand your patient better. The book club is different because you're reading not just about you being a doctor and the **[unintelligible 00:11:48]**. It was kind of part of a larger world view, and I think that helped me a lot because I think sometimes you want to get out of just thinking about medicine, just thinking about your job. And although it helped you relate, it also kind of helped you move in a world beyond what you're doing, because I remember medical school being very consuming, you were always just thinking about your work and it was great to have half a moment to kind of engage and **[unintelligible 00:12:23]**.

Interviewer: Do you think a more formal medical, like a non-fiction curriculum in medical school would be better than this casual setting?

Participant: You know, I actually don't know. I mean I think, one thing I enjoyed a lot about it was it was one activity I felt like I could do that was outside medical school, because there were very few activities I did outside of medical school. It was kind of like clubs with school, mission trips, studying, and you know seeing friends. There wasn't that much outside of it. So, this became something I really enjoyed getting outside of medical school, and you know it's nice that people who wanted to be there and were and everyone was very enthusiastic about it. So, that's what I enjoyed about it.

Interviewer: Okay, and you know, the last question—are you familiar with professional identity formation? I know it's kind of a complex concept. An experience that helped you become—facilitates your learning to become a doctor. So, okay, well thank you very much. And I'm going to turn this off.

STUDENT 9

Participant: Sounds good.

File Name: Audio_08_18_2018_19_43_05NAME.mp3

Interviewer: Okay. With all those disclaimers out of the way, do you have questions about what we're doing or why?

Participant: Nope!

Interviewer: Alright, so we just like to start the interview by asking you some general questions about your memories of the book club. How did you get involved?

Participant: So, I got involved— there was a medical student the year above me, NAME, who had been at the book club and told me about it, so I joined as a result of that. I'm trying— I don't think I quite remember what year of medical school that I joined, but after NAME left I actually took over as coordinator for the book club with Dr. NAME and I have very positive memories and its really— one thing that I think is really nice about this book club was the fact that— two things. One: we didn't just pick books that were topically related to medicine, although we did discuss some books that had overlaps. But two: what I really think made it special was the fact that it was for faculty and students. I don't- I can't remember if there were residents. I mostly remember medical students and faculty as the structure.

Interviewer: Okay, and do you remember your first meeting? Do you remember the first meeting you attended?

Participant: Oh gosh, now it's been so long that I don't. But, I— you know I remember we read "Cutting for Stone" by Abraham Verghese. I remember **[unintelligible 00:04:12]** hosted one of them at her place. I think Dr. NAME had hosted at her place as well. But I would **[unintelligible 00:04:21]** if I tried to remember the exact first book club.

Interviewer: Do you know about how many meetings you attended? Or could you estimate?

Participant: I think I attended them all for at least two if not three years. I don't actually remember what year it was started. Do you know that?

Interviewer: No, I don't. I don't know that.

Participant: I don't think it was that old when I started. So, I graduated in 2011 and so I don't know if it was that much older than that at this point.

Interviewer: Yeah, that's about right. That sounds about right to me as well. So, do you remember any books that had an impact on you in particular? Or anything that stuck out to you in terms of what you read?

Participant: Yes, I remember "Cutting for Stone" by Abraham Verghese and then I remember **[unintelligible 00:05:13]** was another book that we read and I actually ended up seeing— I live in San Francisco now and I ended up seeing **[unintelligible 00:05:24]** who wrote **[unintelligible 00:05:25]** give a talk and it reminded me of that— that book club that we talked about **[unintelligible 00:05:32]**. One random fact that I remember is that Dr. NAME's father is actually married to NAME. I don't know if you know that. I think that that had happened during

that time. Like it was somewhat recent that he had gotten remarried at that point. Those were the main memories retained from our book club and the fact that we called it "Strabookmus" and that it's apparently no longer called that.

Interviewer: Yeah, we still call it that. We're still calling it "Strabookmus."

Participant: Oh, that's good to know. It wasn't my name, I think NAME might have come up with it. But I like a good pun.

Interviewer: It's a good one. Do you feel like any of the books that you read or the discussions that you had had an impact on your professional life at all, in clinical care, interactions with patients, anything that you remember?

Participant: I don't know if it was specifically any one book. I think it's more so— I think reading in general provides empathy in terms of learning about others and I think that when you get to share that with others, especially in our clinical field and to share stories of our experiences, it's hard to not think that that's impactful and the care that we provide for our patients and for each other. So, I don't know if it was specific to any one book, but I do think that there's something to be said about having a shared space to really discuss these things and trying to get into the minds of these characters and other people.

Interviewer: And what about an impact on your personal life? Do you feel like reading at that time had an impact on your personal life?

Participant: Yeah, I think it's great for a medical student to be able to actually interact with faculty, not in a clinical setting, and to feel like they are invested in you as people and as human beings. Also, it's nice to just do something outside of medical school. I love to read; I'm actually involved in several book clubs here in the city, one of which is actually residents and faculty as well. So I think it's really nice to go to someone's home and get to see them outside of the walls of the hospital or the clinic.

Interviewer: And kind of along those lines, do you remember what your interactions were like at the book club? Interactions with students, with faculty, with physicians? What were the meetings like?

Participant: I think very laid back. I think that it was nice to sort of see your attendings and your faculty members just be in a relaxed setting when they're sharing personal stories, or when you're just drinking tea or wine or what not. I think we tended to do them during the day during the weekends, so it tended to be more tea and coffee. I think that it was— I'm a fairly informal person who doesn't get intimidated often but I can imagine that maybe for some it may be intimidating at first but then as you get to know the group it gets a little less intimidating over time. To be able to feel like you can share your thoughts and feelings and not be judged for it when your attendings are around.

Interviewer: Do you feel like these interactions kind of extended beyond the realm of the book club. Do you feel like the relationships you built or the interactions you had changed interactions

you had outside of the book club? Or did you feel like it was **[crosstalk 00:08:57]**. Yeah, go ahead.

Participant: I definitely think so. I definitely think that once you open that door it feels like it extends naturally into all settings. So, even though you maintain a sense of being professional, there's a new added layer of warmth in terms of your interactions where you're not just attending and student and faculty member etcetera. There's like a shared common interest that like lends itself to warmer interactions in the clinical settings.

Interviewer: Thinking back on that time, what do you think the benefit of the book club was to you? Why did you attend it?

Participant: I think that a lot of us like to read and it's nice to have a space to be able to read and discuss and also to just have something that's not medicine- another layer of life. I think that's why I've been in book clubs since medical school onwards. I think that it's also really nice for us to gain insight into different narratives, and once again I think that the more stories you read and the more stories you hear inevitably makes you a better human being and clinician as you learn more about the world.

Interviewer: Did you feel like there was an element of self-care for you with the book club in terms of—

Participant: Yeah, definitely. It's funny, as a medical student I've got to remember my roommate would always make fun of me because I would be like frantically finishing the book the day of the book club, so in all honesty it was a little stressful because I would find that I would be finishing it last minute. But the actual book club itself was always laid back and it wouldn't have mattered if I hadn't finished it. It's just, once again, a nice time to just get to access a different part of yourself.

Interviewer: Do you feel like being a part of the book club made you read more than you otherwise would have?

Participant: Probably. Yeah, I mean it's hard to read as a med student.

Interviewer: And do you think that's important or why would you think that that's a good thing?

Participant: I do think it's important. I mean I think that a lot— a lot of people tend to want **[unintelligible 00:11:27]** in medical school and residency but it's nice to keep in touch with people and maybe **[unintelligible 00:11:32]**. But reading has always been a comfort for me and have found such enjoyment and I did a lot of it growing up and so it was nice to be able to reclaim some of that in times of busyness and in stress.

Interviewer: Definitely. What about the books that you read? Were they books that you would have chosen for yourself or maybe things that you wouldn't have been exposed to?

Participant: The ones that I remember I think they probably would be books that I would have read, but at this point I only remember a handful of them. I get them confused at different time periods in my life.

Interviewer: And you probably remember the ones you liked the best anyway.

Participant: Yeah.

Interviewer: What about—

Participant: They were usually pretty— I feel like they were on best seller novel lists and they weren't necessarily super **[unintelligible 00:12:24]** that I would never have heard of otherwise.

Interviewer: Do you remember the process of choosing books? Or how you guys decided what you were going to read?

Participant: Oh, good question. I feel like we would decide collectively as a group, but maybe that's a lie. Don't quote me on that one- that one I don't remember as well.

Interviewer: Okay. Either at UF or afterwards or before, had you ever been exposed to literature as part of your formal medical training?

Participant: I want to say that we did have some narrative medicine. I don't know if NAME is still at UF—

Interviewer: Yeah, she is.

Participant: Yeah, she did lot of that stuff. Oh NAME. And so, I know there were like narrative medicine components. I remember as a fourth year I talked with a course **[sic.]** we did with the pre-med students that were coming in **[unintelligible 00:13:18]** program and I remember we did some narrative medicine exercise as well. So, there was some incorporation of literature, etcetera, from that stand point. I don't— so right now I'm at UCSF and I'm involved in some medical education and part of— for one of the courses that I help with for the first year medical students we do have a book club that they can participate in where they get to choose one of four novels, and then they can have a book club amongst themselves during the period of time. So, I do think there's an importance to have literature incorporated into the world of medicine.

Interviewer: I wonder if you can talk a little bit about what you think the difference is, if any, between the more formal use of literature in a medical curriculum and kind of the more informal book club.

Participant: So, I think when it's more formal inevitably it becomes mandatory and I think there's a difference between opting-in and electing to do to something like this instead of having it be forced upon you, or an expectation that's forced upon you as another thing that you have to do, even if want to do it. So, I think that the informal nature of the book club allows for this "I chose to do this. This is something that I'm getting to spend my time on. This is important to

me." Also, it's like a smaller, more intimate setting as opposed to when it tends to be in a more formal, large group setting not everyone really has a chance to participate in that. So, off the top of my head those are things that I would think.

Interviewer: Sure, that makes total sense. Any other memories you'd like to share from your time in the book club or any stories that stand out to you as something that sticks with you that you think of when you think of the book club?

Participant: Yeah, like I mentioned I still remember that Dr. NAME **[unintelligible 00:15:16]**—

Interviewer: That is kind of incredible.

Participant: It is. And then, you know what's funny? I still have one of Dr. NAME's books— I think I borrowed the **[unintelligible 00:15:31]** and then never returned it— I still have that book [laughter]. So, if she wants it back, she can have it back. I wonder if she's ever wondered where that book went. I thought about that when I moved and was putting up books I was like "I don't think that book was ever mine." And I'm trying to think if there's anything— anything else. I think it was more just being in attendings' homes and just having the informality around it.

Interviewer: And what about fellow students who are in the book club with you? Did you feel that you formed relationships with them outside of the book club? Or was it just kind of hit or miss?

Participant: I feel like— I think they either already people that I hung out with or— actually, my roommate was one of them, so we obviously lived together. I don't think any new friendships were formed, but I do once again— it's the same thing like you start to say "hi" when you see each other in the hall, especially for those who were in different classes.

Interviewer: And, as you kind of have continued with your career, have you continued to participate in book clubs or continue to kind of be more attentive to literature?

Participant: Yeah, I'm in two book clubs actually. I'm in a book club with people not in medicine and then I just joined a book club with residents and other faculty members and I actually just hosted the last one. For me, I love to read and, once again, I think literature is important in terms of keeping me sane and self-care but then also offering viewpoints that are different from my own. It's nice, I admit having more time now to do those things, but it's also nice just to get to know residents in a different setting.

Interviewer: Okay, well I really appreciate your time. That's all the questions I have for you as a part of the interview.

STUDENT 10
Interviewer: So, to begin with, could you tell me how you got involved with the book club?

Participant: Sure. On the UF College of Medicine Class of 2022 page, somebody named- I had it right here in my e-mail. I sent an e-mail to Dr. NAME on the recommendation of a **[unintelligible 00:00:21]**. It said like "join the book club listserv." And I have been a big proponent of book clubs in the past. You know, when I was working for a while, I had a book club with some friends and it was just always such a joy and I kind of used it as a way of meeting people. So, I was like "okay, I don't know anybody in Gainesville and I really want to join into this and I love reading." So, I jumped on that Facebook post and that was, if you can believe it, August 19th that I sent that message. So, way early in school.

Interviewer: Wow, yeah. So, you were like "yes, book club! Sign me up!"

Participant: I was like, "sign me up." I mean, I did fully acknowledge "I don't know when I'm going to have time to read." But, I guess I had wishful thinking. Hoping it would be okay, and that I would be able—it was really important to me to kind of keep, I suppose, my sanity through reading and having that little bit of an outlet.

Interviewer: And what was the—I mean were you actually able to read a book for the first meeting? Were you actually able to come?

Participant: Yeah. So, the first meeting that I went to was October, and we read *Disgrace* and it was thankfully a really short read. And I read it in like a long weekend, I think in a single day. It was only 200 pages. But, it was nice to kind of take that break.

Interviewer: So, it was like a break between courses; you sat down and were like "let's read this."

Participant: Yeah.

Interviewer: And do you remember the first meeting? Where was it and what was it like?

Participant: Yes, the first meeting was at, I want to say…

Interviewer: You can just say one of the attendings, that's fine.

Participant: Yeah, one of the attendings- I want to say her name is NAME. Does that sound familiar? Anyway, yeah so it was a little nerve-racking going the first time. I went with a fellow MS1 and that was good to kind of not go alone. But, I would have been happy to go alone anyway. But, it was good. Everyone was very welcoming- a very diverse crowd. There was a fourth-year med student, several attendings, there was an undergrad, so just like really interesting perspectives. What else do I remember? I remember that I brought cookies because I thought I should bring something to somebody's house, but then I—yeah, so.

Interviewer: What did you think of the book and how did the discussion go?

Participant: The book was interesting. I always love when book clubs pick books that I wouldn't have picked for myself. And this one was—the topic was like kind of a man that got

into a little bit of a relationship scandal in South Africa, and then was a little bit ostracized from society and it was just a really interesting, interesting read and not something that I might have like picked up if I was going to read on the beach or something, so I always love discussions that stem from that. Do I remember what the discussion was about? Not particularly. I think we talked a lot about him as a character and whether we liked him or not, which is always interesting because sometimes I feel like characters aren't supposed to be liked, and I think that that came up as well. He's not a very likable person, but he's still the protagonist of the book, so where does that leave you and so, it was interesting. And then also just discussion stemming from what does disgrace mean in his context, in other contexts. Then, later in the book he becomes slightly disfigured and then so that kind of stems to a different kind of—like how does any sort of physical ailment lead to any sort of feeling of disgrace, and things like that. So, I really enjoyed it enough to go back a second time. So, yeah.

Interviewer: So, the last one you went to was the most recent one?

Participant: Yes. So, the most recent one was January 6th. I think also, what I like about book club is that you kind of talk about the next book at the previous session, so if you don't going very regularly, it's hard to kind of feel that connection with oh we picked this next book and now we're all committing a little bit of time to reading it so we that we can have a nice discussion. So, I do feel that little bit of accountability and I'm hoping that I can continue that. So, we read a very fun book, a little more of one that I would have picked up just on my own this most recent session and wonderfully, we had the holiday break where I could really just read for fun. So, this one wasn't a struggle to get through, and it was slightly longer. It was called *How to Stop* Time. It was about a man that ages at a different rate than the rest of the world and how that influences his ability to interact with others and what he's doing with his life, since he has more time than one normally would. And the discussion was really interesting, especially because I've read a lot of books similar that are sort of fantasy, and I've enjoyed them. So, hearing kind of attending physicians talking about this book and liking it or not liking it, it was interesting to be on the same page with people that are later in their career and seeing that we can all kind of enjoy the same types of books and things like that. So, I love that kind of connection with people and seeing that, you know, you have connection with many, many people and that you should always get together and share opinions.

Interviewer: And do you always feel comfortable sharing opinions, in the context of the book club? Did it take a while?

Participant: Yeah. I mean, I'm not—I don't know I think it would depend if I had some sort of controversial opinion, but I think it's all very in their nature and you kind of go with the flow of the conversation. So, I enjoyed the book, some people didn't enjoy it as much, so they were talking about reasoning for that, so it's generally—I don't feel that the conversation is in anyway impacted by the array of levels of your professional career that you're in, of whether or not you can share your opinions, in anyway. I think it's a really easy conversation to join.

Interview: And, what are your interactions like with other students, are they confined to the book club? Do you see students outside of the book club? Do you talk about non-book club things?

Participant: Yeah, so I—the other students in the book club, that I've met- so there's like a huge listserv, but I've only met the same group of people the two times I've been. There's a fellow first-year that I think you're going to talk to later tonight that went with me to the first one, went with me to the second one, and we're really good friends. So, yeah. I see and talk to him all the time. There's an undergrad that's in the book club. And, I don't see her around really but if I did I'd say, "hi, we're in book club." It's actually been interesting because I've run into some of the attending faculty members at other functions and been like, "hi, nice to see you again. I'm from book club." And so that's nice to have that pre-connection, and it makes it easier to approach these people in other settings.

Interviewer: Have you worked with any of them clinically or in class?

Participant: I have not. No, because I know Dr. NAME is a CLG leader, but she's not mine. But, I ran into her and…

Interviewer: Dr. NAME?

Participant: Yeah, Dr. NAME- at an open house that they were having for internal medicine at the College of Medicine. So, I went to that and then I ran into her and we got to talking and that was outside of book club, so that was nice. And it was nice to see that she remembered me, and it was transferable and all of that.

Interviewer: What is the benefit of the book club for you? Why do you go?

Participant: I think I like having something outside of school, and I love to read and I love that it's kind of introducing me to a different group of people than the people that I see every day. So, yeah, I think kind of broadening the people I'm exposed to, the opinions I'm exposed to, and kind of being able to use my brain to talk and think about something that's non-school related. It's really—I enjoy it and I think it still adds to my education to be reading books like this, as I go through the medical school process.

Interviewer: Is there an element of self-care for you?

Participant: Yeah, because I love to read and it definitely—being in a book club holds me more accountable I think to keep up with that element because when you're choosing self-care options, reading can sometimes be like "oh, well I need to get extra sleep tonight so I can't read." But, this is nice because I'll read a couple of pages or I actually just signed up—so, I just got a library card, and this has stemmed from the book club too because one of the members of the book club recommended getting a library card because they have e-books and audio books and things available, just through the Alachua County Library. So, I'm signed up to get the audio book for our next session, so I can listen to it on the bus on my way to school, because I've got to try and fit it in and I already kind of use that time just as "me time," and I listen to music. So, I think it'd be a perfect time to be reading something.

Interviewer: Do you think you read more because of the book club?

Participant: Yeah, because as much as I want to, I think that it's nice to have a certain time by which to read it. It just keeps you from putting it on the back burner constantly and it's good.

Interviewer: This is going to be a weird question, because I know you've only read the two books for the club so far, but do you ever find yourself thinking about the books you've read, either for the book club or kind of literature in general, in terms of your professional life? I mean, the two books you read weren't directly medical books, but do you think they impact you as a physician or do you ever think about them in connection to your patients or your practice?

Participant: My practice is very minimal right now. I've basically had one preceptorship, so I feel very, very clinically—

Interviewer: Well, I guess kind of like your identity as a future physician. Do you think about books in general as being related to that and if so, how?

Participant: Yeah, no I mean I definitely think that being exposed to different ways of thinking and life through books affects how I relate with different people and things like that. And also book club is really interesting because, as I mentioned, with the book *Disgraced*, we were talking about kind of like a theme of disgrace and I think there is kind of like a feeling of vulnerability and sometimes even shame that comes from being sick. So, I think being able to hear a bunch of people's understandings of what that term means, is something that resonated with me probably more than if I had heard it just in a lecture, through kind of coming to that in this avenue.

Interviewer: And have you read any literature as part of medical school?

Participant: No, but they keep recommending books that we should read like *On Being Mortal* and things like that that I've like already to read but that I loved—I think books like that are also interesting. It's kind of the same line of like hearing different people's experiences and hearing about the trajectory that they had through medicine and learning to resonate with people and how they shaped themselves. Anything else…in terms…I've read a lot of cookbooks, because I've had to cook a lot. So, not through medical school. I would say definitely book club has been literature, medical school—I've read a lot of up-to-dates.

Interviewer: So, this has been like your main focus for reading.

Participant: Yeah.

Interviewer: Okay, well I really appreciate that and that's all the questions—

STUDENT 11
Interviewer: This interview is just to understand your experiences with the book club. So, if you could just start by telling me how you got involved with the book club.

Participant: It was like four years ago now. I think it was an e-mail that was sent out to the listserv. I responded because I enjoy reading books and I thought it was a nice way to get to

know some faculty members better. And I do think NAME might have mentioned it during "Narrative Medicine."

Interviewer: Okay, so you did Narrative Medicine as well?

Participant: Yes, I did do Narrative Medicine as well.

Interviewer: And do you remember the first meeting of the book club you attended?

Participant: I think the actual first meeting I attended was during either second year or the first year of med school. It took a lot for me to actually go and I think the book was "The Vegetarian."

Interviewer: Okay. What was the meeting like?

Participant: So, I hadn't actually finished reading the book. So, it was kind of trying to contribute at the same time— I don't know saying something like meaningful without disclosing that I didn't completely finish the book.

Interviewer: Right right. So, it was like you wanted to be there, be involved with the community, but also the book hadn't gotten finished so. What about who was at that meeting? Do you remember having any thoughts about who was attending the meeting?

Participant: So, I remember NAME**[unintelligible last name 00:01:57]**, she was there with NAME **[unintelligible last name 00:02:03]**, they were both in my class and that was nice to see familiar faces. I think there were fourth years then actually, **[unintelligible 00:02:14]**. I remember there was an intern, she just graduated, and I think she still had some time to go back to the book club. I haven't seen her since. I think there were a couple of first year students. I remember there was one guy, and he was the only guy there, **[unintelligible 00:02:40]**.

Interviewer: Do you remember what the discussion was like, or how it evolved—

Participant: About the discussion?

Interviewer: Yeah, like who was leading the discussion and how it felt?

Participant: So, the first meeting was at Dr. NAME's house. So, I guess she kind of mediated the discussion. I can't remember— I know for the later ones they used discussion questions from Oprah, apparently Oprah has a book club; she recommends books and gives discussion questions. But it was— the nature of it was very free-flowing and people would talk about what they found interesting in the book and sort of build off of other people's comments.

Interviewer: Sure. As you continue to participate in the book club, do you remember a book that you really liked or you feel like had an impact on you?

Participant: I would say not really. **[crosstalk 00:03:49]**

Interviewer: Were there any that were particularly memorable?

Participant: You're talking about the book now, or the experience?

Interviewer: Yeah the book, or the experience. I mean if there was a memorable experience, but if there was a book that you particularly remember like sticking out to you.

Participant: So, the books I've read, there's only been like three, which includes the one that we're currently reading, but the first one, "The Vegetarian," it was, I guess it was novel to them, but I've already read stories where they were discussing something kind of truthfully, but it was all about mental illness. So, it didn't really strike me as "wow this book is something really novel and intriguing." The second book— oh wait no actually, there was another one. There was a book about the author and her experiences with being raped, it was the one by— it was a really short book and a quick read. But, it almost felt like they were just journal writing and I understand repetition as a literary technique, but in this context, she was— well as a reader I thought she was over-doing the repetition to the point where I almost wanted to put down the book. In the third book— why am I blanking all of the time. Is it helpful for you to have the titles?

Interviewer: No, it doesn't matter. I mean we can refer back to the list.

Participant: The third book was about this young couple where the man gets accused— wrongly accused of raping this other woman who just happened to be at the hotel. And it's this story of how their relationship sort of like falls apart and disintegrates, but they're still friendly with each other kind of thing. I guess that book, I would say of the three, that book had the most impact because it got me thinking about, kind of like, **[unintelligible 00:06:27]** but also within the arena of dating and classes— dating between classes. So, the girl came from a family where her father was a self-made millionaire and a couple of African Americans in the South— African Americans in the South so that gives it some context. Whereas the guy, he comes from a very blue-collar job, tries to find himself in the world and it's interesting that the relationship doesn't work out and they both end up with family friends from the same kind of class.

Interviewer: It sounds like the books that are picked not necessarily books that you would pick for yourself. Does that sound right?

Participant: Uh, yes. That I think is actually a plus for being part of the book club, because I wouldn't have picked these books by myself.

Interviewer: So that's why you continued— **[crosstalk 00:07:28]**. Go ahead.

Participant: It was nice to have people to discuss books. I was actually kind of disappointed with the second one that I couldn't make the book club because one: I actually read the book and two: I hated that book, so I wanted to see what other people thought of it.

Interviewer: Was it— oh gosh, what was the name of that book? I think her last name was Gay, she was talking about how she got—she became obese.

Participant: Yeah.

Interviewer: Yeah, I know I read that book too and I didn't like it either.

Participant: [unintelligible 00:07:56]. I was like "she's just telling me stuff, she's not showing me stuff" and I understand that it was a very traumatic experience and time, but if you're going to write it down it's better to show than to tell.

Interviewer: Yeah, I had problems with that book as well. I mean we had a really interesting discussion at the meeting about discussion. I mean I agree I had a really big problem with the way that book was written, I didn't really like it either, but we ended up talking about what makes people obese and how to deal with obesity in clinic.

Participant: Oh yeah, I think that would be really helpful. And that's how you can really translate that into your practice and with patients.

Interviewer: Yeah, I guess that transitions to the next question, which is to kind of describe your interactions with the members of the book club. So, with other students who attended the book club, did these tend to be people who you were friendly with outside of the book club?

Participant: I mean, aside from NAME and NAME, they were all people that I didn't really know— faculty members aside. Dr. NAME interviewed me for med school. NAME— she taught a few classes, so I'd know them like by face. Dr. NAME **[unintelligible 00:09:29]**. I mean, everyone's really friendly.

Interviewer: So, if you would describe the interactions with faculty members at the book club, what were they like?

Participant: I'd say it's a very open environment where you are welcome to voice disagreement with interpretations of the text and I think that was really nice because it really stimulates discussion.

Interviewer: Do you feel like— **[crosstalk 00:10:05]**. Go ahead.

Participant: At the last meeting, it was interesting when— because the book dealt with infidelity. The guy **[unintelligible 00:10:19]**, the girl's out in, I guess the civilian world, and she ends up cheating on him with her childhood friend who was there at their wedding. But, there was the question of if someone had to be at fault who would it be. So, it's kind of a— even within the personal realm of like intimate relationships and the blame game. It did feel comfortable discussing that.

Interviewer: So, it was like a topic that you would never normally discuss with a faculty member at school. Like who's to blame in an intimate relationship, but in that context, it was okay to discuss with them.

Participant: Yeah, like I can't really think of an opportunity where I would be talking about someone's infidelity. I mean, unless it was a patient I guess, but even then I don't think it's very professional to talk about— like if you had to place blame, which is a very human thing to do, like who is more at fault.

Interviewer: Yeah. Yeah, I mean that's a good point. That's the kind of thing you would just never— it would never enter into the professional world, so this was like an opportunity to have a personal relationship with them.

Participant: Yes.

Interviewer: Have you either worked with any of the faculty members or physicians that come to the book club? Or encountered them outside of the book club and do you feel like your relationship with them has changed?

Participant: I think, yeah, the relationship has grown more, and I feel closer with the faculty members, especially those I wouldn't normally interact with. For example, Dr. NAME, who you hear stories all the time about how she's a hardass. Like, if I wasn't part of the book club I would just be relying on that one class where she taught, and basically all the stories that my friends are telling me. So, it's a good opportunity to get to know a faculty member.

Interviewer: Right, as a person as opposed to a course director.

Participant: Yeah, and you learn about their personal lives too. I think Dr. NAME was talking about doing something **[unintelligible 00:12:49]**, making her little brother do it. I thought it was really funny. Or Dr. NAME talking about her kids being off to college and she started like "Mothers Against Gun Violence" or this group in her **[unintelligible 00:13:07]**.

Interviewer: Yeah. Yeah, that makes sense. So, to think about the book club kind of overall, what do you feel like the benefit of the book club is to you? Why do you go?

Participant: I guess it would kind of be like that hobby you have that keeps things more grounded and remind you that there's more to life than just tests and doing well on rounds or getting papers published. And it's kind of like a reminder of like "hey, this is what normal people do; they read books and talk about it."

Interviewer: Yeah, that totally makes sense. So, it's kind of like— there's an element of self-care in it for you?

Participant: Yeah.

Interviewer: And do you feeling like the book club makes you read more than you otherwise would read?

Participant: In a sense that it lets me read more non-academic, like books and papers not related to like—

Interviewer: Yeah, what you're doing.

Participant: [unintelligible 00:14:24].

Interviewer: Why do you think it's important to build fiction, or to build this kind of reading into your life?

Participant: So, I think fiction is a way for people to discuss through the over-arching themes in, I guess, the art of living. So, fiction allows you to explore all these concepts without them being judgmental and allows you to think abstractly about concepts. So, like this latest book by **[unintelligible author name 00:15:08]**, the South African writer. I've only gotten through the first half or first quarter, and it's about a professor who ends up sleeping with a student who doesn't fully consent, and it's kind of interesting in the sense that I think it's going to generate some interesting discussion. But, it's also written from his point of view and not hers and I think if it was a real-life case it would be a lot easier to demonize the guy, especially within this liberal college setting. Whereas, because we know it's fictional, we're better able to take a step back and say like "hey, what was going through his mind?"

Interviewer: That makes total sense. Yeah, I haven't started that one yet, I have to because I'd like to go to the next meeting.

Participant: Yeah, that'd be fun. They were like "this book is disturbing," and I was like "huh, how?" and then I started reading it.

Interviewer: Do you find it disturbing too?

Participant: So, I understand how it's disturbing, but it's also one of those things where I know it happens and even in the field of anthropology it happens. So, it's not disturbing in the sense of "oh this could never happen, and I could never think of this happening."

Interviewer: Great, that makes sense.

Participant: It is a little uncomfortable that the author decided to take his point of view. So, you're like reading it from the perspective of a sexual predator.

Interviewer: We kind of already talked about this question a little bit, but just to think through it again: are the books the club reads books you'd choose for yourself? And how do they differ?

Participant: So, lately, I've been reading more non-fiction books and much of it is for personal development, trying to understand the stock market, real estate, or how to financially invest kind

of books. So, it's nice to have these fictional books that the book club chooses, and it kind of takes the pressure out of trying to choose that good book that's worth your time. Whereas, within the setting the book club chooses and you just kind of go along, and you're just reading it without thinking— or questioning whether you're wasting your time. At the end, you're still going to be discussing it with the book club.

Interviewer: Yeah, that's a good point.

Participant: So that experience, I think, basically gets rid of that fear of "is this book— did I choose the right book and is this worth my time?"

Interviewer: Right, that makes total sense. Again, we talked about this for a second, but have you ever been exposed to literature as a part of your formal medical training? So, have you ever been in a class setting, as you were trained in medicine, where literature was a part of it?

Participant: So, I don't know if Narrative Medicine counts because it doesn't— it's not like it's actually a part of the curriculum, it's more like a special period you can do this too and get some free time during fourth year. And I have other thoughts about how Narrative Medicine is run, but I think my appreciation of the literature really comes from high school where I had really good teachers and I took AP Lit and AP Language and that ability to read a book and think about concepts and themes and what the author is trying to do, or maybe inadvertently trying to do, there's a whole question of did the author intend to talk about this or not. That, I think, is essential to stimulate. So, I think that's mostly why I'm continuing to go to the book club.

Interviewer: And what do you think are the biggest differences between your experience with Narrative Medicine and your experience with the book club?

Participant: So, I think those who go to book club they actually do try to finish and read the book and they're actually really interested in talking about the book, while those who do Narrative Medicine on the whole are just there to check off a list so they can get free time off during fourth year.

Interviewer: So, the biggest difference is the level of interest and the other people.

Participant: Yeah, and the quality of discussion too.

Interviewer: Yeah, that makes sense.

Participant: I do believe most people didn't read the books.

Interviewer: In Narrative Medicine?

Participant: Yeah, because I know I was one of them.

Interviewer: Yeah, right well, that was a really— I tried to go to Narrative Medicine, but I just could not fit it into my schedule.

Participant: Yeah.

Interviewer: Okay, so that's definitely a difference. If you're going to show up to the book club it's totally totally voluntary, there's no incentive, so the only reason to go is if you've read the book and you really care about the book.

Participant: No, I think part— well that's most of it, but part of it is you also enjoy being with the people at that book club and talking about all these ideas and perspectives.

Interviewer: So, you value— **[crosstalk 00:21:03]**. No, go ahead.

Participant: Oh, so like the talk about fidelity, it was very interesting that all the faculty members who were older would have blamed the girl for cheating, while the other student and I who were younger— oh wait sorry, they blamed the guy, the "side hoe," whereas the other student and I we blamed the girl because we thought there was a contract between the two of them that she broke, and the other person couldn't because he wasn't a part of that contract, kind of thing. So, and then there was another part in that discussion where we discussed the race of the women who accused the husband, because the author was very specific in not describing the race and so some of us assumed the women was black, some of us assumed the women was white, so it was a very interesting discussion.

Interviewer: Yeah, that sounds like a good one. Yeah, I think that was the "American Marriage." I remember when they read it I just didn't have time for it.

Participant: Yeah, the "American Marriage," okay.

Interviewer: Yeah, okay well that's all the questions I have for you and I really appreciate your time. I'm going to stop the recording.

STUDENT 12
Interviewer: Okay so to start with could you tell me how you got involved with the book club?

Participant: Sure. I actually got an e-mail to the med student class that they were having it. Didn't go to the first few during first and second year because I was too busy and finally ended up going to one, just from e-mail.

Interviewer: Do you remember your first meeting?

Participant: I'm not sure actually, which one it was. It was a couple years ago.

Interviewer: Was it in second year? Third year?

Participant: I think it was the beginning of third year, but I'm not sure if I remember— oh, actually it might have been "The Vegetarian."

Interviewer: Okay.

Participant: It might have been that book.

Interviewer: Do you remember that meeting? Do you remember what the meeting was like?

Participant: Do I remember what?

Interviewer: What the meeting was like? The first meeting that you went to?

Participant: Let's see. So, I actually think the first meeting was at the same doctor's house that the one I just went to was at and there were— it was me and one other med student and maybe three physicians. I remember I was kind of nervous because I hadn't met any of them and it was at their house and I'd never been there. But, I don't know, it was nice. It turned out really well.

Interviewer: Do you remember what the discussion was like?

Participant: For the first one? Not really. I think I remember that it just started off with us discussing "oh, well did everyone like the book?" Then, people started asking more specific questions about like certain sections of the book and then I remember it always like— it tends to always go off topic and start, like people start talking about this general **[unintelligible 00:02:18]**. So, I don't know if that's specific to the first meeting, but I feel like that's how all of them sort of go.

Interviewer: Do you remember if there was a book that you particularly— that particularly had an impact on you?

Participant: I would say— I would say the one "Hunger" by I think Roxane Gay was one of the most impactful. I think just because it's a voice that's not heard, like it discusses— it's an obese woman discussing her life and society and the struggles she goes with through daily and I felt a little weird almost at book club discussing it because everyone at the book club was of a normal BMI and we were all discussing how this woman sort of gets through day to day life in the body she's in. But, at the same time I feel like it was a perspective that we all kind of needed to hear to make us more aware of how we treat people struggling with weight in society. So, I think that probably was the one.

Interviewer: Do you think that's impacted the way you care for patients at all? Or your kind of experience with patients?

Participant: So, I think it has because I since them have actually read a few more articles on kind of obesity and being overweight in society and I think that both the book and the discussion and these articles have kind of made me a lot more accepting of people because I think I've come to this conclusion that as a doctor I'm going to be a lot less likely to be able to convince all of my patients to go from obesity to a normal BMI. But, I think I've figured out well what's more important is kind of trying to get them to be healthier, even within the body they're already in. So, I've been like thinking more about like not just saying "you need to completely change

yourself and lose 200 pounds," but more like "okay, how can I get you to start eating healthier, feeling happier about yourself, and getting an exercise plan that works but is still reasonable within what you want to do with your life."

Interviewer: So, you feel like it's made you meet patients where they're at a little more, on that issue?

Participant: Say that again.

Interviewer: So, it's kind of helped you to maybe meet patients more where they're at when it comes to obesity.

Participant: Yeah, exactly. Because I realized like— I was reading this other article that was talking about how you can still be healthy even when you're overweight and it's not always a matter of like "okay you need to lose all of the pounds and go back to being skinny." It's just a matter of "okay how can I help you be healthy, even without you getting to that." So, that was kind of a perspective change for me.

Interviewer: Do you feel like it had an impact on your personal life at all?

Participant: No, I mean I don't think that it changed— I don't think it necessarily changed what I do when it comes to health or eating or anything like that. I would say it changed some of the interactions I have and kind of the judgments I have. I try to be just less judgmental and more compassionate, because I feel like I understand a little bit more **[unintelligible 00:06:25]** it really is for those people and how little— how little people give them credit in day-to-day life.

Interviewer: Yeah, no I had the same reaction with that book. That was a big one for me as well.

Participant: Yeah.

Interviewer: Do you feel like there were any books you read as part of the book club that had an impact on your personal life?

Participant: That have impacted?

Interviewer: Yeah. Or any that you— that stick out to you as being important for you as a person or personal growth?

Participant: I'm trying to remember the different ones we've read.

Interviewer: I can send you a list as well, I probably should have done that. I can pull it up and read you the list.

Participant: Yeah.

Interviewer: Great, hang on.

Participant: I mean I guess— in what way do you mean impact personal life?

Interviewer: I mean it's really up to you. Here I'll read you the last couple: so "American Marriage" was the most recent one, and then before that was "Middlesex," and then we read "Breakthrough: The Discovery of Insulin," "Hunger," "Between the World and Me," "The Vegetarian," and then I don't know these other ones before I don't remember that well: "Exit West," "Land Remembered," "My Name is Lucy Barton," "Fortune Smiles," anyway.

Participant: Yeah, I didn't go back that far. Let's see. I'm having a hard time even remembering what "Between the World and Me" was about.

Interviewer: Yeah, I didn't read that one. So maybe not, that's okay.

Participant: Yeah, not that I can think of.

Interviewer: Yeah, okay. So, let's talk now a little bit about your interactions with other members of the book club. This is a little bit of a weird question coming from me, but what have your interactions been like with other students in the book club?

Participant: Yeah, so for my first three there was a person who's in the class ahead of me who went to all three so I felt like that was kind of nice because we were always the students at the club and so we had that shared connection. And we would always talk about how school was going, or what we were each doing in our respective courses. But, for like the last four clubs or so it's been— it's always been a different student that goes so a lot of times it's someone that I don't know. So, the interaction is usually pretty limited to discussing the book and then I think afterwards we usually tend to just kind of ask "oh what are you— where are you at within medical school? How is it going?" And, I don't know, it's kind of limited to that to be honest.

Interviewer: And what about the faculty or the physicians that go?

Participant: So, I think that's been one of my more favorite parts because it's pretty rare to get to have such a personal connection and feel like I can talk about things like politics or religion or sex or things like that with faculty members, because usually those are taboo topics. So, that's been something I've really enjoyed- just getting to know them a little bit more and kind of realizing that it's okay to sort of discuss our opinions and talk about things completely different than medicine, which is normally all I get to talk to them about.

Interviewer: Do you have any interaction with these faculty members outside of the book club, or do you only see them at the book club?

Participant: Usually it's only at book club. There's one doctor who I've worked with, who taught a bunch of our classes first and second year and I've seen her a few times within the hospital, but that's the only one.

Interviewer: So, for you, what is the benefit of the book club? Why do you go?

Participant: So, a few reasons. One: when I was busier during like third year, it in some ways forced me to read a book every month, and at that point I was so busy that it was the only book I was reading a month. And so, I liked that it was always a good way to find a book and I genuinely just liked the discussions that we would have and the sort of chance to interact with people I wouldn't normally hang out with outside of school.

Interviewer: And is there an element of self-care for you with the book club? Do you feel like that's a part of it?

Participant: Yeah, I do. I think— I think that it's easy in medical school to get trapped up into thinking everything is about medicine and work and patients and so it's nice to have time to be by myself, read a book, and then talk with people from different backgrounds who can kind of share— bring different things to the table. I do find it to be— and it is in some ways self-care because I'm doing something just for me.

Interviewer: Do you think that the book club makes you read more?

Participant: Yes, I think one thing that we always do at the end is people will talk about the books they've read outside of book club, and so I always make a list on my phone of those books and now during fourth year I actually have so much more time and have started reading a lot— a lot more.

Interviewer: That's awesome. We should talk about what you've read because I like your taste in books so I'm going to pick your brain.

Participant: Yeah!

Interviewer: So, why do you think it's important to read during medical school or for medicine in general?

Participant: So, I think we're lucky in going to UF because it has such a focus on humanism and how humanism is important to medicine. But, I think that reading always does that— it does a way better job than any lecture on humanism because you are constantly exposed to new characters from different backgrounds, who are completely different than you, and it reminds you the importance of just meeting people where they are and knowing their background. So, definitely.

Interviewer: Are the books that we read in the book club books that you'd choose for yourself?

Participant: Sometimes yes, and sometimes no. We did one historical fiction which I never would have read, and then we also did one called "The Vegetarian," which was strange. I don't think I ever would have found it on my own.

Interviewer: Interesting.

Participant: But, the other ones that we've read, tend to be more my style. So, yes and no.

Interviewer: Do you like the books that you— that we've read that haven't been your style or do you find them to be kind of harder to get through?

Participant: I think there was only one that I found it hard to get through- the historical fiction one. But, besides that, none of them have been too hard to read.

Interviewer: As part of your formal medical training, so as part of school, have you ever been exposed to literature, like in a class or at all through your formal medical training?

Participant: So, I know when we first started medical school we had to read a book and then we discussed in a small group and in my geriatrics course, which I'm taking right now, we also have to read a book which I actually just started and it's also really good. It's actually a graphic novel. But I think those are the only two times I've had to read books.

Interviewer: So, what were those two experiences like for you?

Participant: They were— well the first one was like "What I Learned in Medical School," I think that was the title, and that was, I thought, was a good book because it sort of helped us all— I don't know talking about it in a small group was just a nice thing to talk about since none of us knew each other so it was already kind of awkward. The second book that I'm reading for geriatrics I really really like, and so I'm actually really glad the course director is having students read it because I don't think any— well, okay, I lied. I don't think most of the people in that class tend to read for fun.

Interviewer: And how are those experiences different than the book club experience?

Participant: Well, the book club has, I think, a better element of discussion. I also think the book club books are not related to medicine, while the other two are somewhat. So, I think I kind of like that the book club is not related to medicine, it's sort of outside of that.

Interviewer: That makes sense. Okay, well those are all the questions. Do you have anything else that you think that we missed, that was important to you or the experience of the book club or anything else that you liked or didn't like about it?

Participant: No, I think it's cool that you're even doing it— I mean I know you said you got roped into it so I'm not sure how voluntary it is but I think it's cool that they're doing something on it.

STUDENT 13
Interviewer: So, if you could just start by telling me a little bit about how you got involved with the book club- what brought you in?

Participant: Sure, you know, as every medical student starts the new year, I've kind of always enjoyed reading and wanted to get involved in something extracurricular, other than the large

amounts of reading we have to do for school. So, as I was sitting in class one day I noticed some people just talking about this book club and I asked them what is was about and they said they got an e-mail or maybe it was a Facebook post, I can't remember exactly, from Dr. NAME. And I asked them about it and joined the e-mail listserv. So, that was how I heard about it.

Interviewer: Okay, and what was the first book that you read?

Participant: The first book that I read—sorry I should know this since I enjoyed that. I think it was *How to Stop Time*.

Interviewer: Okay.

Participant: Yeah, Matt—let me check for you, sorry.

Interviewer: So, the first one you went to was the most recent one?

Participant: Yes, it was *How to Stop Time* by Matt Haig.

Interviewer: Okay, and could you tell me about the first meeting that you attended?

Participant: Yes, the first meeting that I attended—well, what would you like to know specifically?

Interviewer: Just like walk me through what happened and how you found it?

Participant: Sure, oh god I can't remember the physician's house that was hosting it. I went with a friend, from class, and she and I drove there, and we were both nervous, obviously, because we didn't know who was going to be there, if we'd know anyone. So, we just walked in and again, everyone was friendly, and everyone was super nice. In the kitchen, they had lots of food. And it was just a very welcoming experience for me and I felt at ease as soon as I met everyone. Everyone was friendly, so it wasn't that sort of physician-student relationship. It was just comfortable and other students were there, and—

Interviewer: Did you know any of the other students? Or any of the attendings?

Participant: Yes, coincidentally I knew one of the fourth-year medical students, actually, from undergraduate. So, I just knew her from past courses we took together in Undergrad here—took some before medical school. And so that just made it easier, but everything was just comfortable, and we sat down around the table there and we started discussing what we were thinking about the book.

Interviewer: And what did you think about the book? Did you like it?

Participant: I really liked it. Actually, I'm sorry I don't think it was that one—I'm trying to remember actually which one we read.

Interviewer: Was it the one in January or the one before that?

Participant: It was the one before that.

Interviewer: Was it *Disgrace*?

Participant: Yes, it was *Disgrace*, I'm sorry. Yes, it was *Disgrace*. I thought that was the most recent one. It was *Disgrace*, yes. And I forget the physician's house where we did *Disgrace*—or sorry, *How to Stop Time* was the most recent one though, was it **[unintelligible 00:03:16]**. So, yeah, so I remember the facts correctly, just not the book name. It was *Disgrace*, so everyone sat around the table and we were discussing what we thought about the read. I think Dr. NAME and a few other physicians had a list of questions for us for certain topics of discussion, which sort of facilitated discussion a little bit more than it would have typically.

Interviewer: So, they were kind of like pre-prepared questions from them?

Participant: I think it was questions that were sort of either from them or just questions that people asked on book forums online, which were still really good questions I thought. But, it just sort of made the interaction easier. And, of course, after answering some of the small questions, then we jumped from topic to topic which is nice, and we each got a voice through the discussion.

Interviewer: Does anything stick out to you from the discussion, just in terms of what you guys talked about? Do you remember anything about what the topics were or anything that you've thought about since then, from that discussion?

Participant: Trying to remember from *Disgrace*—I think it was interesting because one of the book members was actually—this book takes place in South Africa and one of the members that attended the book club was from South Africa, and he had an interesting perspective on Apartheid and the culture of racism that was sort of cultivated there over generations, and how that was broken but still remained in some facets of society. So, I thought that his perspective in it was interesting and made me think of how that would relate to current times in the United States. So, I kind of related that to what's going on now in our culture, with our society, with recent movements that have sprung up. So, that was an interesting part of it. Another one for that book specifically was the, sort of the male-female relationship and sort of the roles that society has placed on the males and females over generations and how that's changing now a days, and what impact that's had on society, so.

Interviewer: So, do you interact with students from the book club outside of meetings?

Participant: Just the one student that's been there currently that's in my class.

Interviewer: And what about the second book from the most recent meeting? It was a little bit of a different read- a little less about social issues and more kind of fantastical. What did you get out of that discussion?

Participant: I think we were sort of unanimous that it was an easy read, when we sort of discussed overall about the book. What I got out of it, I think, for me it was—the book is titled *How to Stop Time*, so for me it was how do I sort of stop time currently, and the whole premise was that you need to live in the present moment. You know, as medical students, or anyone that has a busy life, we tend to get caught up in everything we're doing and looking towards our future- what's our next goal, what's our next deadline, and we sort of don't live in the present time. And these years go by and we find ourselves suddenly at some summit, only to come back down again and repeat it all over again, which I thought the main character of that book was doing the whole time, over hundreds of years. So, I just sort of saw—the most I got out of it was to sort of- you now, how do I as a medical student, as someone who will have a busy career, hopefully in the future, stop my own time and enjoy what I have in the present moment.

Interviewer: And what about with the attendings in the book club- have you seen them outside of the book club at all? Or interacted with them clinically or through classes?

Participant: The only person that I could think of—well Dr. NAME was a speaker at one of our panels for one of the courses I can't remember, but we didn't have an interaction she was just a speaker up there. And, Dr. NAME- I see her around, and when I see her are she says hello and I say hello. But, she hasn't taught us anything yet. But, she is one of the CLG leaders I think.

Interviewer: What do you think about seeing faculty members and attendings kind of outside of school, and in a more social setting or inside their home?

Participant: I think it's wonderful. I think it sort of takes away from, I don't know. I don't know how to describe it. It's sort of like that feeling when you go to class, and you see someone up there lecturing you, a physician, respectable, and you see yourself distant and very different from them. But, then when you see them outside of that setting, you kind of realize that we all have these similarities and it brings people closer I think, and it gives you a new perspective that makes you realize that we're not that different, and I don't know. It makes the relationship I think better. It may be a bit awkward at first perhaps, but in the long term I think it is very good.

Interviewer: When you think back, I know that you have only really read two books for the book club so far- when you think about the books you read through the book club, are there any moments that stuck with you or that you thought were relevant to your career as a physician, or your experiences with patients, or just kind of thinking forward about yourself as a doctor or patients.

Participant: From the books you mean? That we read so far?

Interviewer: Yeah. I know that they weren't really medical at all, but do you ever relate them to what's going on in medical school?

Participant: I think you can relate most of—most books that have some sort philosophical components to everything in life, and for sure, for instance, I think in *Disgrace* it touched a lot about the male-female relationship, like I discussed. As a male physician, with a female patient, you might want to be more open and more sort of aware of some of the problems that could face

them and not be judgmental. You know, as a future male physician, I think that that definitely helps me sort of see their perspective in life and to not be so quick to judge on their life choices perhaps. And, you know in terms of *How to Stop Time*, I'm not sure, but you know.

Interviewer: Yeah, sure. Sometimes the book club will go through cycles of reading more medically-oriented books, and sometimes we do more fun books, more literature, so you definitely don't have to relate everything to medicine.

Participant: And I think it helps overall, in terms of every interaction we have, whether that's with colleagues or patients, I think matters, and the more you read the more you sort of get new perspectives on different cultures, different backgrounds, different schools of thought. Then, I think that this definitely helps overall, even though you might not be able to specifically pin point something in particular.

Interviewer: So, why do you go to the book club? What is the benefit for you?

Participant: I think this is all a benefit. For one, I need something personally to sort of take me away from just a lot of the science and a lot of the facts that we read throughout medical school. And we can always get bogged down in these details and forget that there's other stuff that we should be involved in outside of medical school. This for me- I love reading so it's one of those ways to sort of make it a part of medical school while being an enjoyable hobby, versus sort of that something—it's something that's low pressure, it's low commitment so I can go if I can, I can read it if I can. But, I still try and make an effort to because I'll see people that I enjoy having a conversation with, discussion with, and you know if—I wish more students were involved in it because then we could sort of push each other to be accountable to read the book and attend these meetings.

Interviewer: Do you think there's an element of self-care to the book club?

Participant: Definitely. I think that taking those one hour, half and a half, two hours just for the meeting itself is very helpful in sort of getting away from it all and just having a conversation about something that is non-medically—well, I meant non-medically related in terms of you know, just factual based science. It's definitely beneficial for me because I love reading, so this is sort of what I want to do in my free time and I try now to make more of an effort to do this and I enjoy reading the book that whatever we chose for book club, by the busy schedules.

Interviewer: Do you feel like it makes you read more?

Participant: Yes, for sure. Like I said, it expands my horizon in more ways than medical school would otherwise.

Interviewer: Do you feel like the books that you read as part of the book club are books that you would choose for yourself?

Participant: I'm personally still stuck on a lot of the classics, but I do actually, because one of my goals in life is sort of to read more of the new books that are coming out, so definitely

Disgrace, which is not that new technically but *How to Stop Time*- I think that this is really helping sort of branch out and discover new authors, instead of being stuck in the classical period, 200 years ago. So yeah, I think that if someone were to guide me I would definitely pick up some of these books.

Interviewer: And have you had to read anything or been to exposed to literature at all as a part of medical school or your formal education?

Participant: I don't think so. I mean there are some meetings that I've attended, perhaps like some seminars by physicians that mention an author here and there, but not particularly no. I don't think it's been pushed on us.

Interviewer: Okay, well that is actually all the questions I have for you.

STUDENT 14

Interviewer: Just to begin, could you tell me a little bit about how you got involved in the book club?

Participant: So, I have been getting e-mails about book club, I think, throughout my four years, well five years of medical school because I took a year out to do some research. But, I never really went until I got back from my research year. So, technically I was a fourth-year medical student and I think, what's her name, NAME—was NAME the one running it then? I can't remember her name. But, she was the MD/JD student and so she was still sending out e-mails about book club and so I finally went. I didn't go that many times, I think I only went three times.

Interviewer: Okay. So, do you remember your first meeting that you ever went to with the book club?

Participant: I remember the first meeting. It was at Dr. NAME's—is it NAME or NAME? I can never remember.

Interviewer: Yeah, I don't know I've been saying it as NAME for so long it's awkward for me to ask.

Participant: I'll say NAME then. Yeah, it was at Dr. NAME's house and—or was it Dr. NAME's—it's one of their houses. And we were discussing—we discussed this book. I can't remember its name—I can't remember the name of the book, but it was completely unimpressive. It was not a great read and I kind of thought other people might have enjoyed it, so I started by saying despite all these great reviews everywhere I didn't really like the book, and then everyone seemed to agree. It seemed like nobody really liked the book. So, that was a little bit funny. And then the second one I think we read *Americanah*, which I don't know if you read but it's phenomenal, and of course everyone loved the book. And we had a really great conversation, I remember that second book club.

Interviewer: Can you describe a little bit—well first, do you remember anything about that first book? Like what it was about?

Participant: It was about—it was kind of sad. It was about this, I think it was a young man who had a young teenager or something who shot himself or tried to kill himself. But, instead of dying he just had a bad—he had to live with a bad wound. Do you know this book?

Interviewer: Yeah, *Wolf in White Van*.

Participant: Yes. Yes, that's the one. Yeah, that was the first one.

Interviewer: Could you describe just a little bit what the meeting was like, how it was set up? Either the first meeting or the second meeting, just kind of like how the discussion went?

Participant: Yeah, the first one I don't remember actually as well, and it was shorter as well, I remember that. The second one—I can describe the second one, that's the *Americanah* book club. So, who was present? It was me, it was Faze who was a year behind me. It was—Dr. NAME was there. Sorry, it's been a while, I don't remember the names of people, but there were maybe like five or six people there.

Interviewer: Okay, so like two faculty members and the rest students?

Participant: Yeah, I'd say—I think it was two faculty members. Or I guess, who's the lady, I guess she's faculty as well, she runs some of the other courses, she's like Serbian or something. She's American but she has like a Serbian last name. Do you know who I'm talking about?

Interviewer: Yeah, I do.

Participant: So, anyway, yeah. So, it might have been like six people there. So, *Americanah* is a really interesting book, I don't know if you've read it.

Interviewer: I haven't read it.

Participant: It's basically about this Nigerian, who is studying in America, and I guess living in America. Basically, it's kind of like- she talks about her relationships with her previous lover in Nigeria versus her American lovers. And also, about basically, how she interacted with white Americans, but also with black Americans, and other immigrants. She makes observations about how different immigrant communities, or even how white people and black people interact with each other, and they're very smart observations. And how she as a black, but not you know a black person from Africa, how she interacted with other black people. So, it was interesting. It was just a really interested read. She talks about her own mental health, she actually talks about being depressed for a while, so I think that came up during the book club. And, yeah. So, I can't really remember exactly what we talked about, but I do remember that mental health came up. I don't remember the exact conversation, but I remember Faze bringing that up. And basically, he felt that the way she described being depressed was actually a very—it was very eye-opening to him, just the description of her depression.

Interviewer: So, who led the discussion? Who did you think—I know that this is a long time for you, so feel free to tell me "I don't really remember," that's totally acceptable.

Participant: Yeah, I couldn't really tell you who led the discussion. But, from my general sense it wasn't anyone in particular always leading the discussion.

Interviewer: Okay. So, do you feel like this book *Americanah* had an impact on you? And if so, what kind of impact?

Participant: So, so do you mean like in my personal life or in my professional life or in my medical school life?

Interviewer: Either, or both. Yeah, either or both.

Participant: Yes, I think it's probably one of my favorite books I've read because just her, her observations were—I mean I, as an immigrant myself, felt that she was making the same observations that I had made. So, I was agreeing with her a lot. So, in that sense it did make an impact because kind of like, I was reading someone who was saying things that I had often seen or felt in similar ways. So, in that sense it did. I think book clubs in general, or I think books like these in general, give us insight into other people's lives, you know, and I think that helps with—from a medical perspective, I think empathy is really important, and I think that when you read certain books, they help you understand you other people's lives and that helps develops empathy in us as medical professionals. I'm in oncology right now, so that's particularly important because I develop really close relationships with my patients and their families because they're going through very difficult times. So, I think that I continue reading and books like these help, despite the fact that medical training is tough and it's long hours, but it's important not to lose empathy and I think literature helps, even non-medical- especially books like these.

Interviewer: So, especially non-medical books?

Participant: Yeah, I think so. I mean I've read all the classic books that everyone reads, as far as medical. You know, *House of God* or Abraham Verghese or so forth. But, I actually enjoy reading other things more because even though they don't directly address medical things necessarily, but I think those books also help just kind of understanding other human beings, which again, I think develops empathy.

Interviewer: So, when you're thinking about the book club, what do you think the benefit of it was for you?

Participant: So—

Interviewer: So, we talked about the development of empathy as a kind of part of being a better physician; what about you personally? Do you feel like there was an element of self-care that was a part of the book club?

Participant: Yeah, I think—I think there is an element of self-care. So, I should add that—so, I spent a year between my third and fourth year working at the NIH, and so I was part of a small research—there were about like—

Interviewer: Oh, this was like the NIH research fellowship? I've heard about this.

Participant: Yeah, you might have heard of it. So, it's basically medical students from all over the country. And so, I actually ran a book club over there at the NIH for one year, and then I came back. And then I did my residency in Cleveland, and I ran a book club in residency for three years. And so, book clubs were important for me because you really get to know the other, you know, the other people who come to book club, especially those who you don't regularly interact with outside the hospital or outside class. You really get to learn about these people; you get to learn about how they think about things. When there's like controversial things that are brought up, although I don't remember anything from my UF book club, but sometimes you'll read—like in residency we read a lot of books that people really either hated or liked and so it's interesting when you hear how people approach things because everyone in my residency, they're good people and in the hospital everybody would do what's best for the patients, but when we discussed certain ethical situations people still had vastly different opinions, and it would be interesting to see how they thought through things and how they thought about things. So, from that—so, you learn about your co-residents, your friends, your co-fellows, and so forth. And you also actually develop close friendships with those who are book club regulars, and so it kind of helps in that manner as well.

Interviewer: Do you feel like it helps to break down kind of the hierarchy between students and residents and faculty members to have that kind of informal setting?

Participant: So, I've never been in a book club with faculty except for the book club at UF. So, I can't—so in residency it was just a residency book club and at the NIH it was just us. But, I can say that I didn't really know Dr. NAME that well before, even though she leads the—I don't know what she does right now, but she used to lead the course with the physical exams and so forth. So, I didn't know her that well prior to my first book club meeting, so I did get to know her a little bit better. I mean I'm not sure that in the non—I'm not sure that there's like much of a hierarchy, but I guess it depends on the institution. Definitely, it might have been more difficult if there was a hierarchy, but I felt like—I felt very comfortable having these discussions with faculty present. I will say that in residency book club a lot of times, as they do, book clubs devolve into people talking about their work or work experiences, and sometimes talking about their interactions with attendings they may have really disliked or sometimes really liked, so that of course would not happen if there were faculty members there.

Interviewer: Do you feel like the book clubs make you read more? Would you be reading as much if you weren't a part of a book club?

Participant: No, I pretty much read all the time, so the book clubs don't make me read more. The book clubs do make me read things that I might not have read- like the first book that we talked about.

Interviewer: And what do you think about that? Is that a good thing?

Participant: I think it's a good thing because I would not have picked up that book otherwise and I think it would have been nice if there was somebody in that book club who had liked the book, so I would have understood why they liked the book, because the writing was not good, the plot wasn't that great. But, yeah. I think it's always good to—it's good for me because I think I know what I like and so I end up reading just certain genres, and just certain things over and over. So, I don't—I know I don't really like sci-fi, so I never read sci-fi unless I have to do it for a book club. So, just things like that.

Interviewer: And so, we talked about your involvement with book clubs at different stages in your medical trainings- have you ever been exposed to literature as part of your formal medical training? So, as part of your curriculum or something that you're required to do?

Participant: I think in residency we had a course where we had to read like a- it was a short story or something, but it was just for one—it wasn't really- I don't think it was really in the curriculum. I guess, nothing major I'd say. Maybe besides that there was like a two-week course where you did a lot of different things, but a part of it was where you read a short story and then discuss it. I guess kind of like a mini book club. But, besides that- and for medical school I can't really remember much.

Interviewer: Okay, okay. Well, I really appreciate your time- that is all of the interview questions.

STUDENT 15

Interviewer: So, could you start off by telling me how you first got involved with the book club?

Participant: I think—so I heard about it because we got an e-mail about when we first started med school, and I thought I wanted to join but I didn't really get into it. Then, I heard about it from you and NAME I think, and you guys said you had been going so that was kind of what prompted me to go. And then Dr. NAME also. She's one of my roommates CLG leaders and she knew that I was interested and every time she saw me she'd be like "you should come!" And that's how I found out about it and then ended up going a year later I think.

Interviewer: Okay. Do you remember your first meeting?

Participant: Yes, it was—I don't remember when it was. But, the book was about the person who discovered insulin I think.

Interviewer: Yeah, I remember that reading.

Participant: Yeah, that was my first meeting.

File Name: Audio_08_18_2018_19_43_05NAME.mp3

Interviewer: What was it like? What did you think of it?

Participant: I thought it was good. I liked the fact that even though I hadn't finished the book I could still participate in the discussion and I could meet people that—something I've found in med school is that you're very isolated and you only see your people that are like in your class, so I really liked the opportunity to meet people in other years and I also meet professors because then I met Dr. NAME and I was able to get her to help me with an AMA event I was hosting and she came and spoke at that I don't think I would have felt comfortable to meet her and feel comfortable talking to her if I hadn't met her in that situation. So, that first meeting was very productive for me.

Interviewer: What did you think of the book?

Participant: I liked the book. It was—I didn't finish it, because it was kind of long. But, I enjoyed it because it was about science, but it was not just straight up science. It was like historical fiction almost. I liked it; I probably would not have found it on my own. So, it was nice being introduced to that through somebody else doing the research and finding the book

Interviewer: And did you feel comfortable participating in the discussion? Do you remember what the discussion kind of talked about?

Participant: I think I was a little quiet at first, just because since I hadn't finished the book I felt like I didn't know the end of the book, so I didn't feel comfortable initially, but I definitely participated once I got—once we got in the discussion, and never felt uncomfortable or like anyone was judging me or anything. So, I enjoyed the discussion. I don't remember exactly what we talked about, but I think we talked about—I do remember how we talked about research and how research has changed because in the book, the guy who discovered insulin- his research methods were pretty bad and so it was interesting to sort of contrast that to how research is regulated and done now a days to back then, and to realized that even though it was pretty unregulated we got some good things, like insulin, out of it.

Interviewer: Did you—how many other books have you read since that meeting?

Participant: So, I read that book and then I read two other books. I actually haven't attended another meeting, but I read the books for the meeting and I'm currently reading the book for the meeting in January. So, I guess I've read three books total and I'm currently working on my fourth for the book club.

Interviewer: How's the book for January? I bought it and haven't really started it- didn't look like my thing.

Participant: It's interesting. I mean I'm only twenty pages in, so I feel like I can't give a—I don't hate it, but I don't know if I love it yet. I feel like it's an easy read.

Interviewer: Do you remember any of the books that you read for the book club having any impact on you or sticking with you at all?

Participant: Well, the second book we read was actually my recommendation which was, why am I blanking on the name, *Middlesex* by Jeffrey Eugenides, so that's like one of my favorite books so I feel like that had already made an impression and an impact on me so that's why I recommended it. Then, the third book that we read was *American Marriage*, which was about a guy, an African-American guy, who had been convicted- wrongly accused of rape, and then put in jail and how that affected his marriage. I really enjoyed reading that book just because I felt like it was very pertinent to the times and even though it was a work of fiction like seeing the impact of what that's like and getting that just from a different perspective and being able to read that and the book was really well written. I enjoyed it, so. I enjoyed reading the book, so I wouldn't say it had a profound impact on me, but I definitely enjoyed kind of being more motivated to read fiction in my spare time.

Interviewer: And have you found that any of the books that you've read as part of the book club have had an impact on your thoughts about being a doctor or your interactions with patients? Have you thought of any of those books clinically? It sounds like they haven't been super clinical books.

Participant: Well, I think *Middlesex* has always had an impact on me because it's very much from a patient perspective, and how the patient was treated by her doctors and how traumatizing of an experience that was, so I think that's something I kind of think about when I'm interacting with patients who have conditions that maybe aren't fully understood or are maybe difficult for the patient to deal with. And then I think the diabetes insulin book was really interesting because thinking about how research is done now and how research was done back then and kind of the impact research has long-term, because I feel like when you're doing research you're kind of like ugh, not really doing anything, what's the impact going to be. And then, being able to see that in the historical context of saving people's lives and seeing the impact what insulin has done for us in the long term. It was good, I think, in helping you kind of go through the mundane aspects of research and just remembering that there is this big picture.

Interviewer: That makes sense. Could you talk through with me what your interactions have been like with other students as part of the book club?

Participant: So, they've been good. I don't think there were actually that many students at the meeting that I went to. But—

Interviewer: It was just basically me and you. Was NAME there? Maybe one other person.

Participant: What?

Interviewer: I said I'm trying to remember that meeting- who was there. I think it was like me and you and—

Participant: And one other, yeah. I don't even remember who the other student was. My roommate, he's currently in the book club, he hasn't been to a meeting, but he and I have talked about the books so.

Interviewer: Oh cool. So, you guys talk about it even though—he reads them even though he hasn't actually gone to any of the meetings.

Participant: Yeah, so he's out of the country at the last meeting I think. But, he is planning on going to the next meeting upcoming meeting. So, we like talk about the book and he's read them but—

Interviewer: Okay cool, so even though he hasn't been able to go to the meetings you've still been able to have discussions about them. What about with faculty who attend the book club? What are your interactions like with them?

Participant: They were really good. I think they were really welcoming and very good about not making you feel like there's like this divide between faculty and students. It was really nice to being able to interact with them in that setting and Dr. NAME who's in it. And then my CLG leader who also occasionally goes to it, so we've like talked about it outside and they've been—Dr. NAME's been like really welcoming and kind of pushing me to like go, keep coming to meeting and stuff like that, so I've really enjoyed—

Interviewer: Who's your CLG leader?

Participant: Dr. NAME- I don't think she's gone to a lot of meetings but she was pretty excited when she found out that I was in the book club. She was like "I've been to a few," but so, like I said, I got to meet Dr. NAME and got to talk to her and use her for coming and speaking to a different event. So, it's been a great resource.

Interviewer: So, can you talk to me a little bit about what the benefit of the book club is to you and why you attend?

Participant: I really—I mean I used to read a lot. I really enjoy reading and I read a lot before med school, so it is good to kind of force myself to do it because I feel like very often, I have a bookshelf full of books in my room and I'm like "I'm going to read 30 minutes every day," and then I never do because by the time I'm done studying all I want to do is just zone out in front of the tv. So, I think forcing myself to read for 30 minutes every day because I have a deadline is nice, because I feel like I feel so much less stressed when I actually read for pleasure. Like, I've noticed a difference in how I feel during certain blocks when I've been reading. It's just—I just feel so much more, I don't know. It's just nice to have an escape because like when you're watching tv it's easy to just kind of zone out, think about well I need to do this, and I'm stressed about this, so being able to just completely detach is nice.

Interviewer: So, do you feel like there are any other reasons that reading fiction during medical school is beneficial for students?

Participant: I mean, I think books—like making you more well-rounded, and then understanding fiction helps you understand other points of view and other cultures and other people's perspectives, because even though it's fiction the author has perspective, the author has opinions, and you can hear about different people's experiences. So, I think *The American Marriage* is a good example of that, like understanding what the experience is like for somebody like that. *Middlesex* is a great example and I know that other med school's actually read *Middlesex* as part of their curriculum. So, even though it's a work of fiction, I think it's useful in understanding the impact in what the doctor-patient relationship is like and how that can impact the patient in dealing with their diagnosis and how it can impact their family and stuff like that. And also, I think you want to be able to have something to small talk about with patients or other people, and not just be so focused on science. So, being able to have something in common with people that you're seeing on a day-to-day basis- like with your patients, being able to talk about that is always nice.

Interviewer: So, having something like outside of medicine you can talk about.

Participant: Yeah, exactly.

Interviewer: Do you feel like the books that we've read as part of the book club are books that you would choose for yourself?

Participant: I don't know. I don't think so, maybe. I feel like it's been so long since I bought a book or looked into buying a book, that I honestly don't really know just because I have so many books that I bought pre med school that I keep meaning to read, and since I obviously don't read as much now, I haven't really looked into that. So, I'm not sure actually.

Interviewer: Have you ever been exposed to literature as part of your formal medical training? Like as part of actual school or as part of an actual assignment for school?

Participant: In like med school? I don't think so. So, I did a post-bac before I came to med school, which was Georgetown's first year medical school, and they actually required us to read *Middlesex*, that was actually part of our endocrinology course. So, I was exposed to that there, but not at UF.

Interviewer: What was that experience of reading that book as part of that program like?

Participant: So, I actually already read it before, so I reread it. But, it was actually really interesting because obviously the patient has like an endocrinological disorder and I think that you're so focused on understanding the enzymes and understanding the pathways and what this enzyme does to this and what it means when you're missing this, so understanding that in the context of a patient and seeing it's so much more than just you're missing this one enzyme therefore you don't make testosterone—I don't remember what he is—it was like 5-alpha reductase deficiency or something like that. So, understanding that there's such a huge impact and that having this disorder is not only affecting your endocrine function, but it's affecting your psychological function, your reproductive function, it's affecting your family life, it's affecting

your mental health. And seeing how everything plays together was really important and understanding the bigger picture of what each disease means.

Interviewer: And did you guys discuss it as part of that course?

Participant: Yeah, we did. We had small group discussions and then we had to write a reflection I think about the book- that was at the end of the course. So, we were supposed to read it before the course started and then write the reflection at the end.

Interviewer: How do you feel like that was different than or similar to the experience of being a part of this book club? What was similar about it, what was different, and what was the benefit of each?

Participant: So, I think the similarities were the discussion was just kind of open-ended, was just kind of like what did you think about the book? What were your feelings? The differences were that it was kind of given to us in the context of a med school class, so we were supposed to think of the patient perspective specifically and the science behind what the patient was dealing with and how that affected the rest of their patient experience. So, it was a little more focused on you're going to be a doctor one day, this is important for you to understand, as opposed to a more open-ended "what did you think about this book."

Interviewer: And did you feel like in that setting, I don't know I'm just thinking out loud, as part of like an optional book club you know everyone is there because they love to read, did you feel like when it was assigned as part of the curriculum it was different because some people weren't as enthusiastic about reading fiction? Or do you feel like was everybody kind of—

Participant: Well, I think everybody at least made an effort to kind of have a discussion and I think it definitely forced people to read that book and think of that perspective when they wouldn't have usually, because I'm sure most of the people in the class were like "why am I reading this book, I'm not in an English course," or whatever. And I know a lot of people did complain about it because it seems like a lot to have to read a 200-page book when you're also studying and everything. But, I think it was useful because everyone was pretty good about contributing to the discussion.

Interviewer: Okay, so maybe like at first people were a little resistant but everybody kind of got into it.

Participant: Yeah.

Interviewer: Cool. Okay, well that's all the questions I have for you. I really appreciate your time.

STUDENT 16
Interviewer: our first question is: how did you get involved in the book club?

File Name: Audio_08_18_2018_19_43_05NAME.mp3

Participant: I think it was just e-mails, and I was interested and then joined up when I was in medical school, if I remember correctly. I think it was just something- kind of an e-mail thing and I like to read so I got involved.

Interviewer: Yeah. Do you remember your first meeting?

Participant: I don't—one of the first one's I'm remembering was at your house, but I don't know that it was my first meeting.

Interviewer: I remember you coming to my house too but I—

Participant: But I don't know if that was my first meeting or if it was a subsequent meeting. I believe I also attended one at NAME's house, and I don't know which one was first. So, I guess maybe I remember the first meeting.

Interviewer: What was it like?

Participant: Well, I think it was a good opportunity as a medical—well I think book clubs are good in generally to get people together to read as a common interest, but then kind of providing like kind of a support system, but almost more like a social event that brings people together through a common interest of reading, so I think it was an opportunity, as a medical student at least, to meet some faculty in a space that was outside of class or clinical care- that kind of a thing. So, that was I think helpful.

Interviewer: I think that's what we all liked about it, yeah. Do you remember any of the books having an impact on you? I know it's hard to even remember which ones you've read, let alone which ones you read for the club.

Participant: Right, because I read a lot of these books, but I don't remember which ones I read for the club. I don't know that I can remember that, I'm sorry if that's unhelpful.

Interviewer: Yeah- did you read *Americanah*? That was one at my house.

Participant: No, well I had not read that book and that was when I was in fellowship **[crosstalk 00:02:11]**. So, these were the ones—I mean I graduated here. These are the only ones that I was even in medical school.

Interviewer: *The Room.* I think that one was called *Room*.

Participant: Yeah, it's called *Room* and I do remember—I mean that one was an impactful book. I mean I can still remember that story because that was intense. I actually think that I helped—me and Sasha helped you pick out books because we were like the student liaisons for it at the very end of my—

Interviewer: Did we go over brunch?

Participant: We did.

Interviewer: Oh, yeah! It's all coming back.

Participant: We went to that place "Sisters" in Haile Village, and I want to say I had something to do in picking out *Room* and I believe—or *Devil in the White City*, one of the two. And I believe I read *The Boy Who Harnessed the Wind* too. I don't remember—and I believe I've read *State of Wonder* as well, but I'm not remembering those books as I much as I remember *Room* and I remember *Devil in the White City*.

Interviewer: *State of Wonder* is one about the Amazon, and there's that fertility medication, that OBGYN—

Participant: I did, I did read that. I don't know that I went to the meeting because I want to say I was on—I was in a mission trip in Nicaragua, when we read it.

Interviewer: Oh, when we were reading it. Yeah. Do you remember anything about the brunch that we had and just like thinking about the books and stuff, I just never think about that?

Participant: Yeah, I think that was fun. I think, again, it was a nice opportunity to have contact and kind of like mentorship in a different way than the typical mentorship about your career path. I mean I think it helps to preach the whole "well-rounded" and "wellness" thing when you're meeting with faculty members and things that are—doing these kinds of things and while the book club may not be for everybody, but people who like to read generally it is something that helps them to feel good and to have balance in their life, you know.

Interviewer: Cool, yeah. I agree. Do you—I know it's hard because you don't remember exactly what books you read for the book club, but do you remember any of the books having a particular impact on your interactions with patients or clinical care?

Participant: I don't think so. The ones that I read for the book club that I'm seeing down here- no, I don't think so. I mean, I read a lot so I think in general there are books that I read that impact my clinical care probably in a roundabout way, because reading is thought provoking, so a lot of things you read are human experiences, whether it's fiction or a non-fiction human story—I think reading about human stories helps you to be more empathetic or to have a more open-mind about things you think about. So, I don't know if I can—this is going back quite a few years, so I don't know if I can remember specifically a book that was specifically book club that I read that impacted my patient-care, but I think in general reading does probably impact my interactions with people- being more aware of human stories.

Interviewer: Yeah, yeah. Well, I'll ask again even though you don't remember well. Are there some books that might have impacted your personal life more?

Participant: Probably, again. I think in general reading does impact my personal life, because again it's something that I do that's a kind of activity for me. I enjoy reading.

Interviewer: Just taking the time to do it is enjoyable, no matter what the content is, you're saying.

Participant: Right. So, I think it helps me be a more well-rounded person and to feel a little more at ease and it's kind of a way to escape sometimes. So, I would say yes, but I don't remember any specifics.

Interviewer: So, you were a pretty big reader, it sounds like, before you joined the book club.

Participant: Yeah, and I have been since. I'm always reading. I'm always reading something.

Interviewer: So, it didn't really make you more of a reader, because you already kind of were.

Participant: Yeah, I'm a reader. Always have been, always will be. I grew up reading lots of books.

Interviewer: -going to the library.

Participant: Yeah, always. I'm always reading, so.

Interviewer: What were your interactions like with members of the book club?

Participant: Yeah, so I mean I think I mostly—I interacted with you a lot—

Interviewer: We kind of already knew each other, but I guess you already kind of knew all the faculty somehow.

Participant: So, I think it was just again, restating things I've said already, but kind of a more casual interaction, with a common interest. So, I think it does break down some of the med student-attending kind of dynamics, then makes people more equal in the sense that you're meeting up about a common interest, everyone has something to bring to the table at a more equal level, when you're meeting in a social environment and talking about a book that everybody read and so I think that all the interactions I had were positive. I also—you know, Sasha was in the book club and so was NAME- I'm trying to remember the other students. I can actually remember now that you bring up what that book was about, *State of Wonder*, NAME and I were both reading that book when we were in Nicaragua in our mission trip, so we chatted about it and stuff.

Interviewer: I don't even see NAME on here. We came up with a list of everyone we could remember, I don't know if this jogs your memory at all, but I don't see NAME on there. What was her last name?

Participant: Oh, NAME was in it? I don't remember NAME being in the book club either.

Interviewer: Yeah. Was she in your year? NAME, was she in your year?

Participant: No, NAME was the year above me.

Interviewer: Okay.

Participant: That looks like most of the people, so there are only a handful of people that I remember.

Interviewer: Okay, I'll have to get that contact information for NAME from you because we can ask her too.

Participant: Yeah. I think—I don't know if she ever came to a meeting or if she was just planning to because she was a reader and read some of the books.

Interviewer: So, people just—we found out later that a lot of people read all the books, but they never came, which was kind of neat to think there's this kind of cloud of people who are benefitting from it even though they didn't come.

Participant: Right, right. And I can't remember if she ever came or if she was just intending to come and then never made it. Sometimes there's like a complex, if you're reading the book and you're intending to come, but then—

Interviewer: You're stressed because you have an exam—

Participant: Or something, yeah.

Interviewer: Well, you sort of already answered this, but I'll ask you again in case it sort of jogs any other answers. What was the benefit of the book club for you? Why did you attend? Is there an element of self-care?

Participant: Yeah, I think it's definitely an element of self-care and I think being in a book club also holds you accountable. Not everyone in the book club reads the book, I think it depends on what kind of book club. I feel like this was a book club more that people probably weren't going to come to if they didn't read the book.

Interviewer: They did! Over the years, they sometimes did, which was always fine.

Participant: Did they? I'm part of a book club now—there's like a core group that always reads the book, then there's some that come, and they sometimes read the book and sometimes don't, you know. But, yeah, no reading is definitely self-care for me and being a part of a book club does hold you accountable to reading the book and thinking about the book because then you go and talk about it, which I think is good to get your mind thinking. And I think also in medicine we're so like, you know, there's kind of a sort of regimen in the way we think. Creativity does come into it, but I think not quite as often as we may like. You know, we're thinking "oh this medication class or that medication class or what is this pattern that is forming or what is my differential diagnosis and what is the next steps." Whereas I think reading and analyzing and thinking about a book in a different way is kind of a different skill and different part of your

brain that you're using, so I think that that's also kind of interesting—you know a benefit and refreshing thing to exercise and not let that become rusty.

Interviewer: Good point, good point. Let's see, anything else. Do you think it made you read more, or you were already reading a lot—

Participant: Probably in med school it did. Because, I'm trying to remember if I was always reading a book regularly in med school, and I presume that I was, but I can't remember. I know in residency I was always reading a book for pleasure even if it would take me forever and since residency I'm always reading something, sometimes I'm reading two books. So, I think I was probably always reading a book in medical school, I could be wrong about that though, because we read so much other stuff in medical school that I don't know if I always kept up with that, but I probably did. It might have encouraged me to read a book quicker, even if I had other things going on because of kind of the deadline of "oh, we're going to be talking about the book, I want to finish it so that something doesn't get given away," or something. I don't know.

Interviewer: It's sounds like you were the kind of person who was going to finish the book if you were going to come, which a lot of people are. And were there—are the books that the book club read books that you would have chosen for yourself?

Participant: Sometimes yes, sometimes no.

Interviewer: So, you met some books you might not have come across. Were you ever exposed to literature as part of your formal medical training? Do you remember ever being assigned anything?

Participant: So, I know there was a, what was it called, but it was an elective and not everyone took it.

Interviewer: Oh- Humanities—you mean with NAME.

Participant: I never did it but—

Interviewer: Medical Humanities? That's not what it was called.

Participant: Yeah, I forget what it was called. What was it?

Interviewer: Narrative Medicine!

Participant: Narrative Medicine- that's exactly what it was called. So, I never did that, but then I did do, I think this was during fourth year there was some sort of undergrad course that had the flavor of Narrative Medicine, and medical students would volunteer, or apply and you got selected, helped NAME to run the course. And you were assigned some of the classes and that had literature reading that was of the medical flavor, but like the medical Narrative Medicine kind of stuff. So, I did read some of that stuff because I had to read it to prepare to be able to

teach it. So, I never took the Narrative Medicine course, but then I helped teach- I can't remember what that course was called.

Interviewer: Something that was for the junior Honors students?

Participant: Yes, that's exactly what it was. And I was one of the medical students that helped to teach that. So, I did read, and so that was almost a curriculum in and of itself because I had to do the curriculum to be able to teach the curriculum. And then I did have, in Palliative Care Fellowship, there was one book that we did, that she does with her fellows every year. And then I also took an ethics course during—it was like a graduate level ethics, medical ethics course that they offered to the Palliative Fellows. It was optional, but they pay for it if you did it. It was at Einstein College, in the medical school or whatever, who are the people that did it. So, I did take that and that had lots of assigned readings that were kind of of the medical flavor in some way and then we would talk about—actually some of these on here, like *When the Spirit Catches You and You Fall Down*, I read that for that ethics course. So, yeah. So, yes, I have had assigned readings but not in my medical education. In my medical training, but not formally in medical school.

Interviewer: Yeah, interesting. I bet Palliative Care is one of the only—yeah, I don't know if other fellows—

Participant: I mean granted it was only one book, and she does it, I guess, with the fellows every year, and it's a fairly brief—I'm trying to remember, it's a—I'm trying to remember, I can remember the book, but I can't remember the name of it.

Interviewer: Oh, it wasn't *Spirit Catches You*, that was for the ethics—

Participant: No, that one was for the ethics course, which I also read during that year. But, I read a lot of stuff for the ethics class, more than just that book. We had a couple, two or three books, during the class and then lots of short readings that we had. It was a lot of reading and a lot writing. It was a lot of prep work, but it was worth it.

Interviewer: Were there medical students in the class or was it all—

Participant: So, the class was, I don't know if there were any medical students. It was a wide variety of people. So, some of the people were getting their graduate degree in like medical ethics or bioethics, and then people were taking it just for a certificate, which is what I was taking it for. I was taking it for a certificate, not for the—because I wasn't doing other course work to get a graduate level degree. So, there was a wide variety. So, there were people within the medical field, there were lawyers in the class, there's people who wanted to be medical ethicists, there was nurses, there was a few other doctors, there was a wide variety. And it was actually very interesting because a lot of the course was discussion. And the teachers, one was a lawyer, one was a physician, one like literature was her thing, and then the other person was a bio ethicist. So, there was a wide variety and they would teach different portions of the course. Very fascinating. So, I did a lot of formal reading for that.

Interviewer: How would you say that was different than the book club?

Participant: Well, that was actually a class. I had assignments, granted it was pass/fail for me because I was just a certificate, but I didn't do—well, I guess I had some assigned writings and while it wasn't like on this specific reading, you were supposed to use the readings in the writing assignments, and then also it was discussion. So, there was an assignment—you know, lots of reading assignments for each week, and if you didn't read them it was much harder to participate in the class, so. So, it was different because it was actual like assignments, rather than just kind of like "read this book over the next month." It was like, read all these things and be prepared for this class at this time, you know.

Interviewer: Anything else that you recall about the book club, now that we've been kind of going a little back in time here?

Participant: Not that I can think of.

Interviewer: Okay, thank you very much.

File Name: Audio_08_18_2018_19_43_05NAME.mp3
