# Supplementary material for: Self-care perspective taking and empathy in a student-faculty book club in the United States
Source: J Educ Eval Health Prof. 2020 Jul 31;17:22. doi: 10.3352/jeehp.2020.17.22 (PMC7577880; doi:10.3352/jeehp.2020.17.22)
Supplement: Supplementary file 2 — Supplement 1. The books selected for the book club [file jeehp-17-22-suppl1.docx]

**Supplement 1. The books selected for the book club**

10/06/2019 The Refugees, Viet Than Nguyen

08/2019 Where the Crawdads Sing, Delia Ownes

04/07/2019 Frankenstein, Mary Shelley

02/17/2019 Virgil Wander, Leif Enger

01/09/2019 How to Stop Time, Matt Haig

10/14/2018 Disgrace, J. M Coetzee

08/19/2018 An American Marriage, Tayari jones

06/03/2018 Middlesex, Jeffrey Eugenides

04/15/2018 Breakthrough: Elizabeth Hughes, the discovering of insulin, Arthur Ainsberg and Thea Cooper

01/2018 Hunger, Roxane Gay

10/2017 Between the World and Me, Ta-Nehisi coates

08/2017 The Vvegetarian, Han Kang

06/11/2017 Exit west, Mohsin Hamid

04/20/2017 Land Remembered, Patrick D. Smith

12/2016 My name is Lucy Barton, Elizabeth Strout

10/2016 Fortune Smiles, Adam Johnson

08/2016 Imagine Me Gone, Adam Haslett

06/2016 Tsar of Love and Techno, Anthony Marra

03/2016 Lila, Marilynne Robinson

01/2016 Fates and Furies, Lauren Goff

11/2015 Lolita, Vladimir Nabokov

08/2015 Buried Giant, Kazuo Ishiguro

05/2015 Girl on the train, Paula Hawkins

03/2015 Wolf in White Van, John Darnielle

01/2015 Americanah, Chimamanda Ngozi Adichie

10/2013 A Study In Scarlet, Sherlock Holmes

09/2013 Ender’s Game, Orson Scott Card

07/2013 The 100 Year Old Man Who Climbed Out the Window and Disappeared, Jonas Jonasson

05/2013 Death comes to Pemberley, P. D. James

03/17/2013 The Shadow of the Wind,Carlos Ruiz Zafón

01/13/2013 This is How You Lose Her, Junot Diaz

11/14/2012 Love in the Time of Cholera, Gabriel Garcia Marquez

09/02/2012 Charltan,Pope Brock

07/16/2012 Dreams of Joy, Lisa See

05/12/2012 Animal, Vegetable, Miracle, Barbara Kingsolver

03/2012 State of Wonder, Ann Patchett

11/2011 Room, Emma Donoghue

08/2011 Devil in the White City, Erik Larson

08/2010 The Boy Who Harnessed the Wind, Bryan Mealer and William Kamkwamba
